# Competitive cyclization of ethyl trifluoroacetoacetate and methyl ketones with 1,3-diamino-2-propanol into hydrogenated oxazolo- and pyrimido-condensed pyridones

**DOI:** 10.3762/bjoc.21.209

**Published:** 2025-12-17

**Authors:** Svetlana O Kushch, Marina V Goryaeva, Yanina V Burgart, Marina A Ezhikova, Mikhail I Kodess, Pavel A Slepukhin, Alexandrina S Volobueva, Vladimir V Zarubaev, Victor I Saloutin

**Affiliations:** 1 Postovsky Institute of Organic Synthesis of the Ural Branch of the Russian Academy of Sciences, S. Kovalevskoy St. 22/20, 620066 Ekaterinburg, Russian Federation; 2 Saint-Petersburg Pasteur Institute, Mira St. 14, 197101 Saint-Petersburg, Russian Federation

**Keywords:** condensed pyridones, 1,3-diamino-2-propanol, ethyl 4,4,4-trifluoroacetoacetate, methyl ketones, three-component cyclization

## Abstract

The use of 1,3-diamino-2-propanol with competitive *N*- and *O*-nucleophilic centers in a three-component cyclization with ethyl 4,4,4-trifluoroacetoacetate and methyl ketones enables the synthesis to be carried out for octahydropyrido[1,2-*a*]pyrimidin-6-ones and hexahydrooxazolo[3,2-*a*]pyridin-5-ones, the preferential formation of which depends on the substituent in the methyl ketone component. Dual acid–base catalysis of the reactions with alkyl methyl ketones increases the regioselectivity in the synthesis of octahydropyrido[1,2-*a*]pyrimidinones. The cyclization with acetophenone is characterized by the regiospecific generation of these bicycles. The presence of three chiral centers in the synthesized bicycles, depending on the alkyl substituent, causes the formation of two to four diastereomers, the structure of which has been determined with ^1^H, ^19^F, ^13^C, 2D ^1^H-^13^C HSQC/HMBC, ^1^H-^1^H COSY/NOESY NMR and X-ray diffraction analysis.

## Introduction

The modern strategy of organic synthesis is aimed at conforming to the "green chemistry" principles based on PASE (pot, atom, step, economic) methods. These principles serve as the foundation for multicomponent processes that allow various structurally complex molecules to be obtained in one stage using commercially available reagents [1–3].

Their ambident properties make 3-oxo esters convenient reagents for the use in multicomponent syntheses, with the Hantzsch [4–5] and Biginelli [6–8] reactions being the best known. In the transformations, 3-oxo esters are cyclized with aldehydes and ammonia (monoamines) or urea to yield a certain type of products only: pyridines and pyrimidines; and formation of the products proceeds with the involvement of an acyl fragment and the *meso*-position of an oxo ester component [9]. Although the introduction of 2-aminoazoles into the Biginelli reaction allows azolo[1,5-*a*]pyrimidines [10–16] to be synthesized, in general, the range of products obtained in these syntheses is predictable and limited.

Ethyl trifluoroacetoacetate and its polyfluoroalkyl-containing analogues are also used widely as starting substrates in multicomponent syntheses [17–19]; at the same time, they often show extraordinary reactivity. For example, the introduction of polyfluoroalkyl-3-oxo esters into the aforementioned Hantzsch and Biginelli reactions leads to the hydrated heterocycles, which can provide insight into the mechanistic aspects of these transformations.

Our team has discovered a new multicomponent synthesis that is based on autocatalyzed reactions of ethyl trifluoroacetoacetate and other polyfluoroalkyl-3-oxo esters with α-methylenecarbonyl compounds and amines [9]. This approach is appropriate only for 3-polyfluoroalkyl-3-oxo esters containing an activated carbonyl group capable of adding α-methylene ketones. It is characterized by the cyclization at the 1,3-dicarbonyl fragment of 3-oxo ester and offers a possibility of using a variety of nucleophilic agents to form hydrogenated diastereomeric hetero- and carbocycles. To date, we have proposed new protocols for the synthesis of 2-pyridones [20], imidazo[1,2-*a*]pyridones and pyrido[1,2-*a*]pyrimidinones [21–24], pyrido[2,1-*b*]oxazinones and oxazolo[3,2-*a*]pyridones [25] using ammonia, 1,2- and 1,3-diamines or 1,2- and 1,3-amino alcohols, respectively. The introduction of cycloketones has resulted in the same types of heterocyclic systems, yet they are now carboannelated, which are synthetic analogues of alkaloids [26]. Moreover, it has been shown that acetaldehyde is capable of reacting in a similar manner with ethyl trifluoroacetoacetate and ethylenediamine [27] as well as with 2-(aminomethyl)aniline and 1,3-diaminopropane [28]. In addition, we have found another route for the three-component cyclization of 3-polyfluoroalkyl-3-oxo esters with α-methylene ketones involving mono- and dialkylamines, leading to 5-polyfluoroalkylaminocyclohexen-2-ones [29]. In general, the developed approach allows pyridone derivatives to be generated in various environments of condensed carbo- and heterocyclic structures. Among the synthesized pyridone derivatives we have found analgesic [20,26], antibacterial [20] and antifungal [20] agents, which show practical promise for the three-component approach we have proposed.

**Figure 1:**
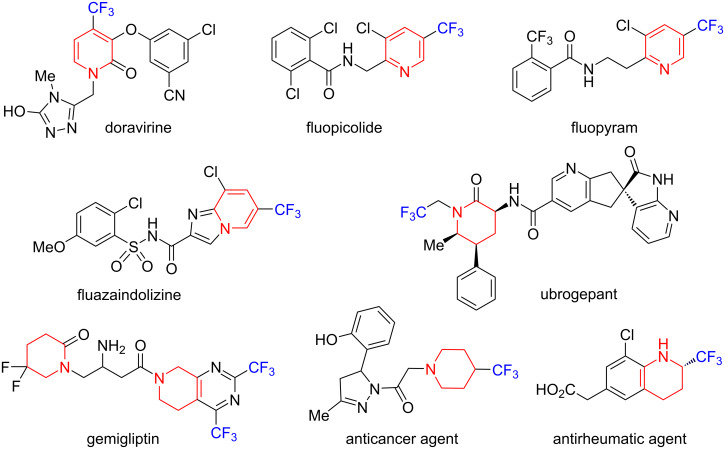
Structures of bioactive molecules with trifluoromethylpyridine and piperidine frameworks.

The importance of trifluoromethyl-containing pyridine and piperidine systems is well known for both medical and agrochemical applications [30]. Thus, many medicinal agents have such fragments, for example, the non-nucleoside reverse transcriptase inhibitor doravirine [31], calcitonin gene-related peptide (CGRP) antagonist ubrogepant [32], dipeptidyl peptidase-4 (DPP-4) inhibitor gemigliptin [33]. The trifluoromethyl pyridine framework is also widely used in the development of plant protection products [34], including the fungicides fluopicolide [35], fluopyram [36], and the nematicide fluazaindolisine [37] (Figure 1). The trifluoromethyl piperidine backbone is part of the structure of anticancer [38] and antirheumatic [39] agents.

The fluorine-containing pyridines and their heteroannelated derivatives are known to exhibit a high biological potential, on the one hand, owing to the fact that the pyridine framework is widespread among natural compounds [40–41], and, on the other hand, because of the unique properties of fluorine [42–45].

In this work, 1,3-diaminopropan-2-ol (**3**) has for the first time been introduced into the reaction of ethyl trifluoroacetoacetate (**1**) with methyl ketones **2**. This reagent has alternative *N*- and *O*-reactive centers and thus can react as an *N*,*N*- or *N*,*O*-dinucleophile, generating in a single synthesis trifluoromethyl octahydropyrido[1,2-*a*]pyrimidin-6-ones or hexahydrooxazolo[3,2-*a*]pyridin-5-ones, or both that contain an additional functional group, the presence of which should affect their diastereomeric structure.

We have found only one work describing the use of 1,3-diaminopropan-2-ol to form heterocycles, where it is reported to react with chloroacetaldehyde and carbon dioxide as an *N*,*N*-dinucleophile only, giving hexahydro[1,3]oxazolo[3,4-*a*]pyrimidin-6-one [46]. Here, we have made research efforts to explore the possibility of using 1,3-diaminopropan-2-ol for the synthesis of pyrido[1,2-*a*]pyrimidine and oxazolo[3,2-*a*]pyridine. Our research interest is caused by the potential biological activity of the compounds, since there are antileishmanial [47], antibacterial [48], antimalarial agents [49] as well as COX-2 [50] and FGFR [51] inhibitors found among them. Moreover, various drugs (antipsychotic risperidone [52] and pirenperone [53], antiallergic ramastine [54]) have been obtained on the basis of pyrido[1,2-*a*]pyrimidine; and compositions of antitumor antibiotics of the kigamycin family include the oxazolo[3,2-*a*]pyridine skeleton [55–56].

To obtain oxazolopyridones containing trifluoromethyl substituents, the syntheses have been proposed, which are based on the intramolecular cyclization of 1-phenyl-2-(4-(trifluoromethyl)piperidin-1-yl)ethane-1,2-dione [57], or 4-(4-phenyl-2-(trifluoromethyl)oxazolidin-2-yl)butanoic acid under acidic conditions [58], and condensation of 6,6,6-trifluoro-5-oxohexanoic acid with (*S*)-(+)-phenylglycine [59]. The data on the multicomponent synthesis of fluoroalkyl-containing pyrido[1,2-*a*]pyrimidines are limited to our studies only [21–24]; however, there are various one- [60] and two-component [61–65] approaches to obtaining trifluoromethyl-containing pyrido[1,2-*a*]pyrimidines available in literature.

## Results and Discussion

**Scheme 1:**
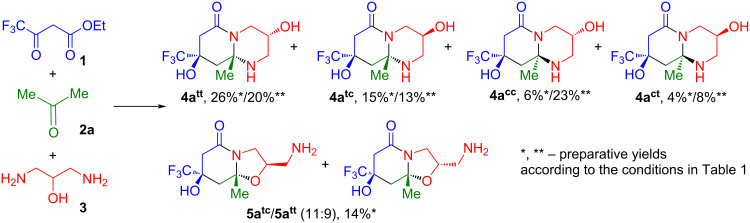
The reaction of ethyl trifluoroacetoacetate (**1**), acetone (**2a**) and 1,3- diaminopropan-2-ol (**3**).

We began our research by searching for optimal conditions for the reaction of ethyl trifluoroacetoacetate (**1**) and acetone (**2a**) with 1,3-diaminopropan-2-ol (**3**) (Scheme 1). The selection of conditions is necessary, because alternative octahydropyrido[1,2-*a*]pyrimidin-6-one **4** and hexahydrooxazolo[3,2-*a*]pyridin-5-one **5** can be formed due to the presence of three nucleophilic centers in 1,3-diaminopropan-2-ol (**3**), and because there is a need to increase diastereoselectivity of the cyclization process. Table 1 presents the optimization steps for the reaction conditions at various solvents and temperature.

**Table 1:** Optimization of the reaction conditions for ethyl trifluoroacetoacetate (**1**), acetone (**2a**) and 1,3-diaminopropan-2-ol (**3**).

entry	conditions^a^	*Т*, °С	time	composition of the reaction mixture [%]^b^							

				**1** ^c^	**4a** **^tt^**	**4a** **^tc^**	**4a** **^cc^**	**4a** **^ct^**	**5a** **^tc^**	**5a** **^tt^**	others^d^

1	1,4-dioxane	25	4 d	2	27	17	7	10	**10**	11	16
2	MeCN	25	4 d	3	**33**	8	6	10	8	12	20
3	THF	25	4 d	12	25	17	5	5	4	6	26
4	EtOH	25	4 d	8	20	13	7	12	8	10	22
5*	1,4-dioxane	60	24 h	–	31	**20**	9	5	7	11	17
6	1,4-dioxane^e^	60	5 h	2	24	15	8	9	8	11	23
7	1,4-dioxane^f^	60	24 h	–	10	6	11	**13**	5	**13**	42
8	1,4-dioxane^g^	60	24 h	–	4	3	5	7	5	11	61
9	1,4-dioxane^h^	60	24 h	–	27	18	10	11	9	11	14
10**	1,4-dioxane^i^	60	24 h	–	22	17	**28**	10	4	6	13
11	DCE^e,j^	60	10 h	–	8	4	9	**14**	5	4	56

^a^Reactions were carried out with **1** (1 mmol), **2a** (1 mmol) and **3** (1 mmol) in 2 mL of the solvent. ^b^Determined by ^19^F NMR analysis of the mixture: **4a^tt^** (δ_F_ 79.52 ppm), **4a^tc^** (δ_F_ 79.61 ppm), **4a^cc^** (δ_F_ 79.91 ppm), **4a^ct^** (δ_F_ 80.02 ppm), **5a^tc^** (δ_F_ 79.66 ppm), **5a^tt^** (δ_F_ 79.74 ppm). ^c^Unreacted residue of ester **1**. ^d^Unidentified products. ^e^The reaction was performed in a closed vial in a CEM Discover microwave synthesizer, MW 50 W. ^f^1 mmol of AcOH was used. ^g^2 mmol of AcOH was used. ^h^1 mmol of Et_3_N was used. ^i^2 mmol of AcOH and Et_3_N were used. ^j^0.2 mmol of DMAP was used.

The reaction course was monitored by TLC and ^19^F NMR spectroscopy. In all the four solvents used, both types of products were formed: octahydropyrido[1,2-*a*]pyrimidin-6-one as four diastereomers **4a^tt^** (δ_F_ 79.52 ppm), **4a^tc^** (δ_F_ 79.61 ppm), **4a^cc^** (δ_F_ 79.91 ppm), **4a^ct^** (δ_F_ 80.02 ppm); and hexahydrooxazolo[3,2-*a*]pyridin-5-one as two diastereomers **5a^tc^** (δ_F_ 79.66 ppm) and **5a^tt^** (δ_F_ 79.74 ppm) (Table 1, entries 1–4). The synthesis in 1,4-dioxane resulted in the highest conversion and the least amount of by-products, but the reaction proceeded slowly over four days. To speed up the process, the synthesis in dioxane was carried out by heating at 60 °C (Table 1, entry 5) in a microwave (MW) reactor (Table 1, entry 6). It turned out that after 24 hours of heating the complete conversion of oxo ester **1** occurred and the overall yield increased to 83% (Table 1, entry 5), whereas the use of MW reduced the reaction time down to five hours (Table 1, entry 6), but the contents of by-products were observed to increase to 23%.

Under all conditions used, the predominant formation of the *trans,trans*-diastereomeric form of octahydropyrido[1,2-*a*]pyrimidin-6-one **4a^tt^** was observed (with the maximum amount of 33% in polar MeCN). Heating in dioxane affected the diastereomeric composition of the products, increasing the content of the *trans,cis*-form **4a^tc^** to 20%.

The use of 1,4-dioxane with acetic acid catalysis at different molar ratios (Table 1, entries 7, 8) led to the formation of a large fraction of unidentified by-products (42–61%), whereas the base catalysis with triethylamine in 1,4-dioxane (Table 1, entry 9) did not affect the reaction course significantly. The use of conditions with catalysis by a combination of Bronsted acid and base (acetic acid, Et_3_N) contributed to a shift in the reaction selectivity towards octahydropyridopyrimidinones **4** (overall yield 71%) (Table 1, entry 10), with the formation of the *cis,cis*-bicycle **4a^cc^** as the predominant product (28%), while the proportion of hexahydrooxazolopyridones **5** was minimal (10%) compared to all other conditions.

The highest content of hexahydrooxazolo[3,2-*a*]pyridin-5-one **5a** was observed at room temperature in 1,4-dioxane (diastereomer **5a^tc^**, 10%) and 1,4-dioxane with acetic acid at 60 °C (diastereomer **5a^tt^**, 13%). The attempts were made to increase their proportion further by using the conditions (DCE, 4-DMAP (20%), MW, 60 °C) (Table 1, entry 11); those conditions have proved effective for the synthesis of octahydrocyclopenta[*b*]oxazolo[3,2-*a*]pyridin-5-ones in the reactions of 3-oxo ester **1** and cycloketones with amino alcohols [26]. However, these attempts turned out to be unsuccessful because of the increased occurrence of side processes (56%); however, an increase in the proportion of the *cis,trans*-octahydropyridopyrimidinone **4a^ct^** to 14% was observed.

We succeeded in isolating all six products **4** and **5** from the reaction of ester **1**, acetone (**2a**), amine **3** in dioxane (Scheme 1). Octahydropyrido[1,2-*a*]pyrimidinones **4a^ct^** and **4a^tt^**, which were formed in larger quantities, were the first to be isolated: the bicycle **4a^ct^** precipitated from the reaction, which allowed it to be easily isolated and purified by crystallization from MeCN. Diastereomer **4a^tt^** was obtained from the filtrate also by crystallization from MeCN. Column chromatography allowed us to isolate octahydropyrido[1,2-*a*]pyrimidinones **4a^tc^**, **4a^cc^** in an individual form, and hexahydrooxazolo[3,2-*a*]pyridones **5a^tc^** and **5a^tt^** in a mixture at a ratio of 11:9.

**Scheme 2:**
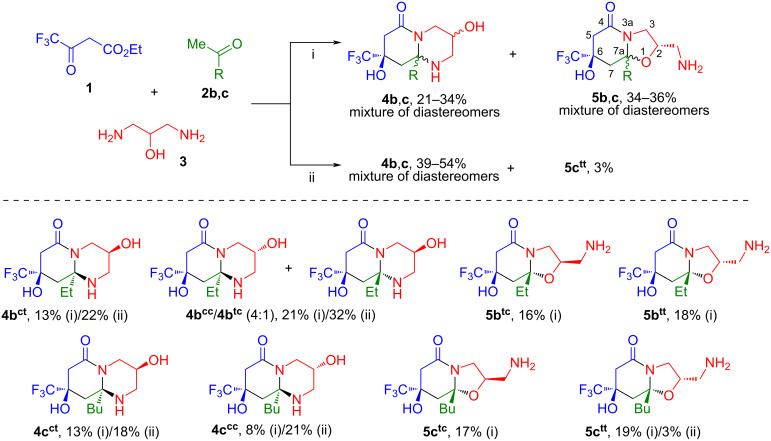
Three-component reaction of ethyl trifluoroacetoacetate (**1**), alkyl methyl ketones **2b**,**c** and 1,3-diaminopropan-2-ol (**3**). Reaction conditions: i. 1,4-dioxane, 60 °C; ii. 1,4-dioxane, AcOH, Et_3_N, 60 °C.

To carry out similar reactions of ethyl trifluoroacetoacetate (**1**) and 1,3-diaminopropan-2-ol (**3**) with methyl ketones **2b–d**, the two most productive conditions were selected: heating at 60 °C in 1,4-dioxane, either without catalysis or using triethylamine–acetic acid as a bifunctional catalyst (Scheme 2).

The reactions with alkyl methyl ketones **2b**,**c** in 1,4-dioxane without catalysis led to the formation of a mixture of octahydropyrido[1,2-*a*]pyrimidinones **4b**,**c** and hexahydrooxazolo[3,2-*a*]pyridones **5b**,**c**, but with a predominance of the latter and an another diastereomeric composition of the products. In the reaction with 2-butanone (**2b**), three diastereomers of **4b** and two diastereomers of **5b** were formed. The bicycles **4b^ct^** (δ_F_ 80.09 ppm), **5b^tc^** (δ_F_ 79.65 ppm), **5b^tt^** (δ_F_ 79.73 ppm) were isolated in an individual form, and the octahydropyrido[1,2-*a*]pyrimidinones **4b^cс^** (δ_F_ 80.00 ppm) and **4b^tс^** (δ_F_ 79.50 ppm) were obtained in a mixture at a ratio of 4:1 (Scheme 2). The transformations with 2-hexanone (**2c**) led to the formation of two diastereomers of **4c^ct^** (δ_F_ 80.09 ppm) and **4c^cc^** (δ_F_ 80.00 ppm), two diastereomers of **5c^tc^** (δ_F_ 79.65 ppm) and **5c^tt^** (δ_F_ 79.74 ppm). All products were individually isolated by column chromatography.

Carrying out these syntheses in 1,4-dioxane in the presence of acetic acid and triethylamine increased the selectivity for the formation of octahydropyrido[1,2-*a*]pyrimidinones **4** in the reactions with alkyl methyl ketones **2b**,**c** (Scheme 2), similar to the transformations with acetone **2a** (Scheme 1). The conversion and the preparative yields of products **4b**,**c** and **5b**,**c** are given in Table 2.

**Table 2:** The conversion and the preparative yields of products **4b**,**c** and **5b**,**c**.

products	δ_F,_ ppm	1,4-dioxane, 60 °C		1,4-dioxane, AcOH, Et_3_N, 60 °C	

		^19^F NMR data, %	yield, %	^19^F NMR data, %	yield, %

R = Et					

**4b** **^cc^**	80.00	21	21 (4:1)	**36**	32 (4:1)
**4b** **^tc^**	79.50	6		9	
**4b** **^ct^**	80.09	17	13	**26**	22
**5b** **^tc^**	79.65	**21**	16	6	–
**5b** **^tt^**	79.73	**22**	18	9	–

R = Bu					

**4c** **^cc^**	80.00	14	8	**25**	21
**4c** **^ct^**	80.09	18	13	**21**	18
**5c** **^tc^**	79.65	**23**	17	4	–
**5c** **^tt^**	79.74	**25**	19	7	3

**Scheme 3:**
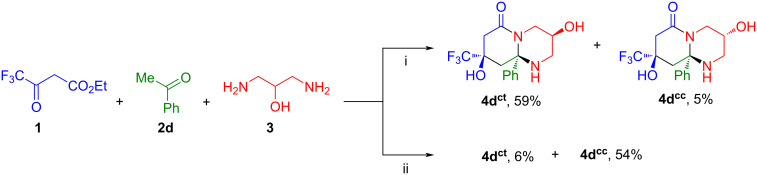
Three-component reaction of ethyl trifluoroacetoacetate (**1**), acetophenone (**2d**) and 1,3-diaminopropan-2-ol (**3**). Reaction conditions: i. 1,4-dioxane, 60 °C; ii. 1,4-dioxane, AcOH, Et_3_N, 60 °C.

The regioselectivity is the main feature of the reaction of ester **1** and diaminopropanol (**3**) with acetophenone (**2d**), because in this case only octahydropyrido[1,2-*a*]pyrimidinone is obtained in the form of *cis,trans*- and *cis,cis*-diastereomers **4d^ct^** (δ_F_ 80.03 ppm) and **4d^cc^** (δ_F_ 79.94 ppm). Carrying out the reaction under AcOH/Et_3_N catalysis allowed the ratio of isomers to be changed, increasing the amount of diastereomer **4d^cc^**, which was minor when heated in 1,4-dioxane (Scheme 3, Table 3).

**Table 3:** Conversion and preparative yields of products **4d**.

products	δ_F,_ ppm	1,4-dioxane, 60 °C		1,4-dioxane, AcOH, Et_3_N, 60 °C	

		^19^F NMR data, %	yield, %	^19^F NMR data, %	yield, %

**4d** **^ct^**	80.03	**68**	59	13	6
**4d** **^cc^**	79.94	14	5	**67**	54

**Scheme 4:**
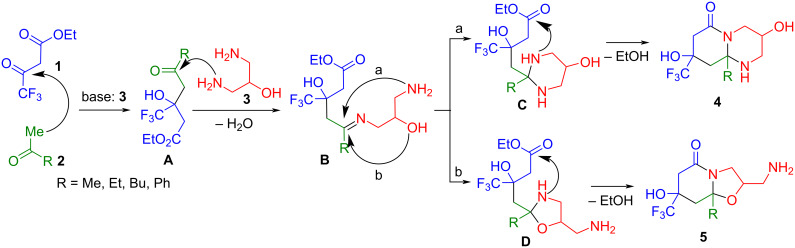
The proposed mechanism of three-component cyclization of 3-oxo ester **1**, methyl ketones **2a–d** and 1,3-diaminopropan-2-ol (**3**).

Previously, we proposed and experimentally confirmed an aldol mechanism underlying the three-component cyclization of polyfluoroalkyl-3-oxo esters with α-methylene ketones and amines in piperidone derivatives. According to this mechanism, the key intermediate is aldol **A**, which is formed from 3-oxo ester **1** and methyl ketone **2** under the catalysis of amine **3** [24–25,29]. Therefore, we suggest that the formation of octahydropyrido[1,2-*a*]pyrimidinones **4** and hexahydrooxazolo[3,2-*a*]pyridones **5** proceeds via the initial formation of aldol **A**, which then reacts at the acyl moiety with the amino group of diamino alcohol **3** to generate a three-component intermediate **B** (Scheme 4). The latter undergoes intramolecular cyclization involving the C=N bond in two equally probable directions: by adding a free amino group to form a hexahydropyrimidine ring of intermediate **C** (path a), or by adding an OH group to generate an oxazolidine ring of intermediate **D** (path b). The subsequent intramolecular cyclization of intermediates **C** and **D** involving a secondary NH group and an ester substituent yields the bicycles **4** and **5**, respectively (Scheme 4).

In the reactions with acetophenone (**2d**), only path a is realized leading to octahydropyrido[1,2-*a*]pyrimidinones **4d**, whereas in the syntheses with alkyl methyl ketones **2a–c**, both paths proceed. At the same time, in the reactions with acetone (**2a**), path a predominates; and in the reactions with 2-butanone (**2b**) and 2-hexanone (**2c**), the ratio of the resulting bicycles **4b**,**c** and **5b**,**c** is approximately the same, with a slight predominance of **5b**,**c** that are formed by path b (Scheme 4). Apparently, in the intermediate **B**, which was obtained from acetophenone (**2d**), the phenyl substituent exerts a strong positive mesomeric effect on the imine reaction center. This makes the center softer, which favors the interaction with the amino group to generate the hexahydropyrimidine intermediate **C**. The methyl, ethyl and butyl substituents of intermediate **B** have a positive inductive effect, which increases with the lengthening of the alkyl chain. As a result, the cyclization becomes competitive in both directions, but with a predominance of path b as the alkyl chain lengthens [66].

The use of acetic acid and triethylamine results in the dominance of path a in all of the reactions. Most likely, the acid catalyzes the imine center in the intermediate **B**, which becomes softer for attack by the amino group.

It is worth noting that the diastereoselectivity of octahydropyrido[1,2-*a*]pyrimidinones formation **4a–c** in the reactions with alkyl methyl ketones **2a–c** is also affected by the length of the alkyl chain, with the number of diastereomers decreasing as the alkyl chain lengthens. Thus, in cyclization with acetone (**2a**), four diastereomers **4a** are formed, with 2-butanone (**2b**) – three isomers **4b**, and with 2-hexanone (**2c**) – two ones **4c**. Also, two isomers **4d** are formed in the reaction with acetophenone (**2d**). This is consistent with our earlier data [24], which showed an increase in the diastereoselectivity in synthesis of octahydropyrido[1,2-*a*]pyrimidinones in the three-component reaction of ethyl trifluoroacetoacetate and 1,3-diaminopropane with methyl ketones with an increase in the volume of substituent in the ketone component.

Unlike the octahydropyrido[1,2-*a*]pyrimidinones **4a–c**, the hexahydrooxazolo[3,2-*a*]pyridones **5a–c** are formed as two diastereomers regardless of the conditions, which may be due to the greater conformational rigidity of the five-membered oxazolidine cycle compared to the six-membered hexahydropyrimidine one.

The structure of octahydropyrido[1,2-*a*]pyrimidinones **4a–d** and hexahydrooxazolo[3,2-*a*]pyridones **5a–c** was determined by IR, ^1^H, ^19^F, ^13^C NMR spectroscopy, two-dimensional NMR experiments, and X-ray diffraction analysis (XRD).

The IR spectra of octahydropyrido[1,2-*a*]pyrimidinones **4a–d** are characterized by reduced vibrational frequencies of the carbonyl function (ν 1633–1593 cm^−1^) and N–H, O–H groups (ν 3435–3126 cm^−1^), which indicates their participation in the hydrogen bond formation [67]. On the other hand, the IR spectra of hexahydrooxazolo[3,2-*a*]pyridones **5a–c** contain intense absorption bands of the C=O function at ν 1652–1628 cm^−1^ and NH_2_ groups at ν 3376–3272 cm^−1^.

**Figure 2:**
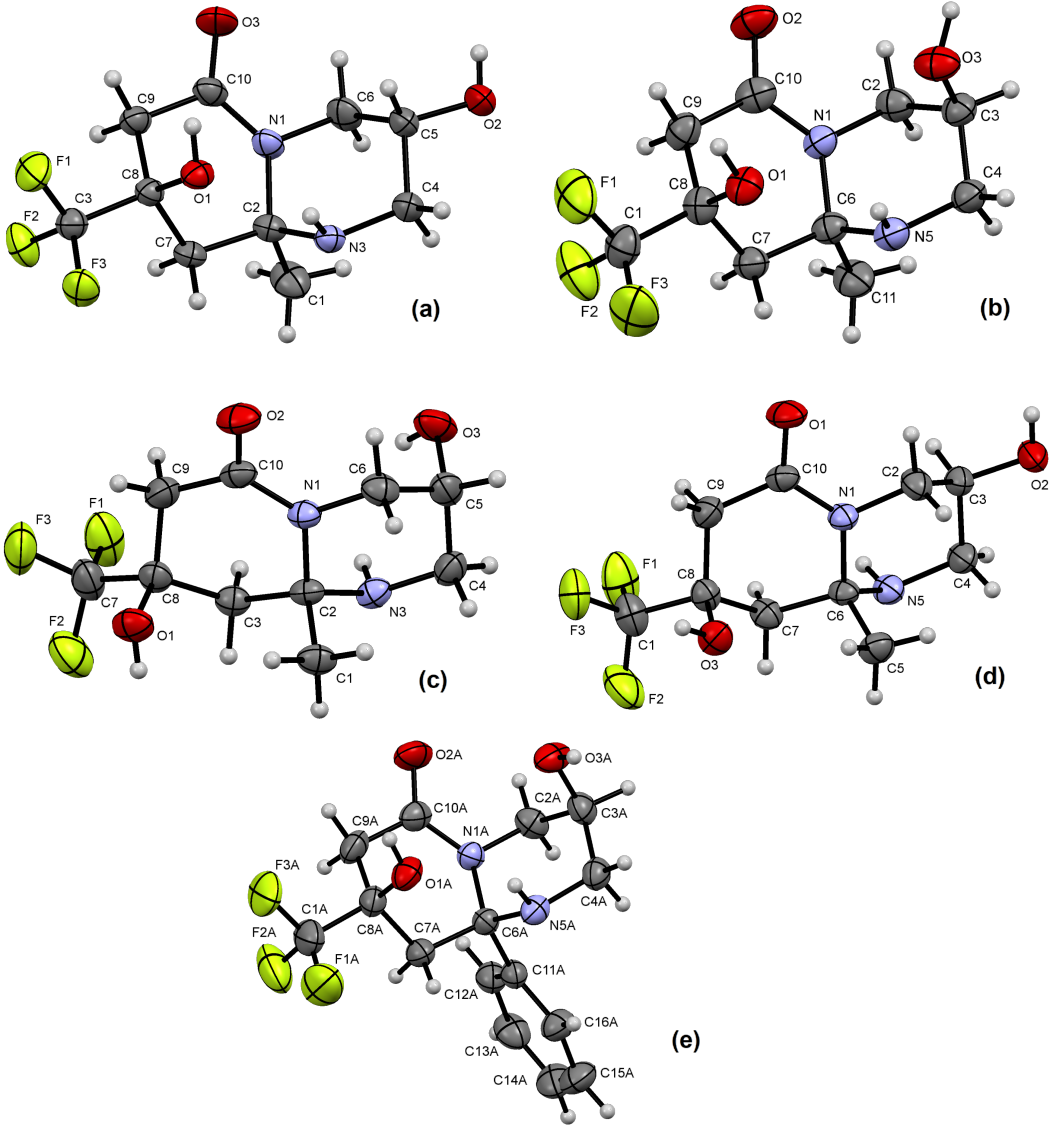
ORTEP view of compounds **4а^сc^** (a, CCDC: 2479553), **4а^ct^** (b, CCDC: 2479554), **4а^tt^** (c, CCDC: 2479555), **4а^tc^** (d, CCDC: 2479556), and **4d^ct^** (e, CCDC: 2479557) showing with the thermal ellipsoids at 50% probability.

**Figure 3:**
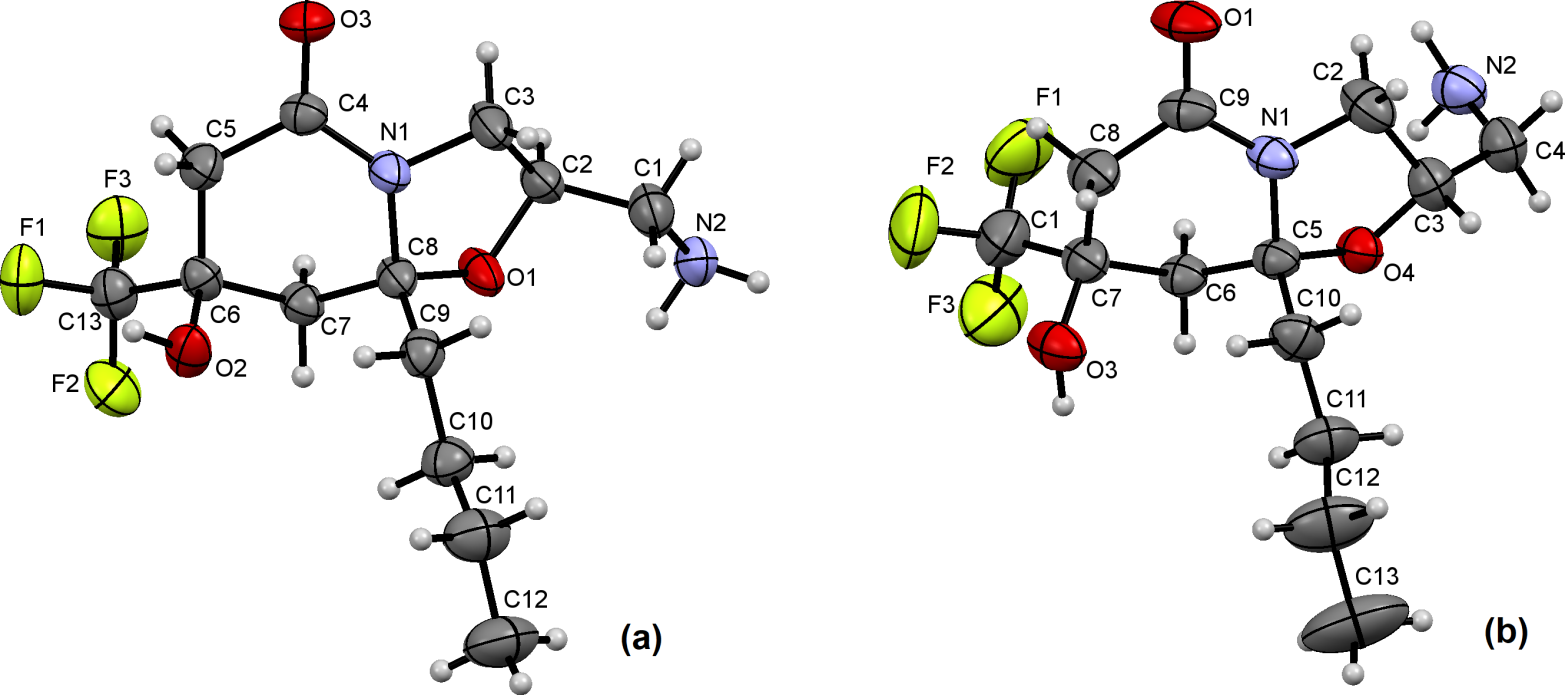
ORTEP view of compound **5c^tc^** (a, CCDC: 2479558), **5c^tt^** (b, CCDC: 2479559) showing with the thermal ellipsoids at 50% probability.

The structures of the obtained heterocycles **4** and **5** contain three stereocenters, so one could expect the formation of up to 8 stereoisomers in each case. Analysis of the ^19^F NMR spectra showed that from two to four diastereomers of octahydropyridopyrimidinones **4** and two diastereomers of hexahydrooxazolopyridones **5** were formed in the transformations under study. The stereoconfiguration of octahydropyridopyrimidinones **4а^cc^**, **4а^ct^**, **4а^tt^**, **4а^tc^**, **4d^ct^** and hexahydrooxazolopyridones **5c^tc^**, **5c^tt^** was determined using XRD and it served as the basis for assigning the configuration of other diastereomers (Figure 2 and Figure 3). Analysis of the XRD results shows that the formation of bicyclic compounds are formed with two types of ring fusion: *trans*- and *cis*-type, similar to the structure of decalin [68].

In all studied structures **4а^cc^**, **4а^ct^**, **4а^tt^**, **4а^tc^**, **4d^ct^**, **5c^tc^**, **5c^tt^** (Figure 2 and Figure 3), the piperidone ring has a half-chair conformation in which the pseudo-equatorially located trifluoromethyl group is a conformational anchor. The five atoms of the six-membered piperidone ring are coplanar, and the trifluoromethyl-substituted carbon atom С(8) is significantly deviated by more than 0.5 Å above or below the ring plane. Accordingly, the adjacent hydroxy substituent has a pseudo-axial position. The hexahydropyrimidine ring in bicycles **4а^cc^**, **4а^ct^**, **4а^tt^**, **4а^tc^**, **4d^ct^** has a chair conformation, and the oxazolidine ring in heterocycles **5c^tc^**, **5c^tt^** has an envelope conformation, with a deviation of the oxygen atoms O(1) by 0.5 Å for **5c^tc^** and nitrogen atoms N(1) by 0.4 Å for **5c^tt^**.

In *trans*-junction, bicycles **4a^tt^**, **4a^tc^** (Figure 2c,d) have a flattened fixed skeleton with a dihedral angle of 24.8–27.3° (see Table S9, in Supporting Information File 1), in which the substituent R at the bridgehead carbon atom can occupy only an axial position with respect to both cycles. On the other hand, the CF_3_ group is a conformational anchor and always occupies a pseudo-equatorial position, which is why it leads to the *trans*-configuration of the piperidone cycle. In the *cis*-junction, the conformation of octahydropyridopyrimidinones **4a^cc^**, **4a^ct^**, and **4d^ct^** (Figure 2a,b,e) is more curved, with a larger dihedral angle of 47.8–53.9°, where both the substituent R at the bridgehead carbon atom and the CF_3_ group are located in pseudo-equatorial positions, and that determines the *cis*-configuration of the piperidone ring. At the same time, in relation to the hexahydropyrimidine ring, the bridgehead substituent R occupies a pseudo-axial position. The relative configuration of the hexahydropyrimidine ring in compounds **4** is determined by the pseudo-axial (*trans*) or equatorial (*cis*) position of the OH group.

The crystal packing of single crystals **4a^tt^**, **4a^tc^**, **4a^cc^**, **4a^ct^**, **4d^ct^** is ordered by intermolecular hydrogen bonds linking O–H groups and heteroatoms O/N of neighboring molecules (see Table S8 in Supporting Information File 1); N–H groups of the hexahydropyrimidine ring and a carbonyl fragment of the neighboring molecule also participate in the pattern of **4a^tt^** crystal packing. In addition, in the *cis*-configuration of the pseudo-axially located NH and OH groups in the structures **4a^cc^**, **4a^ct^** and **4d^ct^**, a strong intramolecular N–H···O hydrogen bond is observed.

The crystal packing of hexahydrooxazolopyridone **5c^tt^** is ordered by intermolecular hydrogen bonds of the NH_2_ group with the hydroxy and carbonyl functions of two other molecules (Figure 3b). The OH substituent of the piperidone ring is in the *trans*-configuration relative to O(4) atom. In the oxazolidine ring, the CH_2_NH_2_ group occupies a pseudo-equatorial position and is in the *trans*-position toward the Bu group. The single crystal structure of the bicycle **5c^tc^** is ordered by intermolecular hydrogen bonds between the hydroxy group and the amino group as well as between the amino group and fluorine of the CF_3_ substituent of the neighboring molecule (Figure 3a). The hydroxy substituent of the piperidone ring is in *cis*-configuration relative to O(4) atom. In the oxazolidine ring, the CH_2_NH_2_ group occupies a pseudo-equatorial position and is placed in the *cis*-position toward the Bu group.

The stereochemical features derived from the XRD data on single crystal **4a–d** and **5a–c** are in excellent agreement with the results of 1D and 2D NMR studies, enabling us to extend these structural assignments to other synthesized bicycles. The complete assignment of signals in ^1^H and ^13^C NMR spectra was performed using two-dimensional ^1^H-^1^H COSY, NOESY and ^1^H-^13^C HSQC, HMBC experiments. The relative configuration was established based on the analysis of spin–spin coupling constants ^3^*J*_H,H_ and ^4^*J*_H,H_ as well as on a set of NOE-correlations.

Equatorial or pseudo-equatorial protons of the methylene groups in compounds **4** and **5** were identified by the long-range coupling constants ^4^*J*_H,H_ = 2.6–3.6 Hz, which is typical for the W-arrangement of atoms. The *trans*-configuration of the piperidone ring is confirmed by NOE correlations between C(8)OH and the protons of the R substituent located in the 1,3-diaxial positions. The *cis*-configuration of the piperidone ring is characterized by NOE correlations between the protons of the pseudo-equatorial substituent R and the axial proton at C(9). The *cis*-configuration of the pyrimidine ring is proven by the cross-peaks in the NOESY spectrum of the protons of axial substituent R and the equatorial C(3)OH with the same H(2)_ax_ proton, whereas in the *trans*-configuration of the hexahydropyrimidine ring, a cross-peak (OH_ax_, H(2)_eq_) appears in the spectrum.

In the ^1^H NMR spectra of octahydropyrido[1,2-*a*]pyrimidinones **4a^tt^**, **4a–d^ct^**, which have the *trans*-configuration of the hexahydropyrimidine ring, the signal of the H(3) proton has the form of an unresolved or poorly resolved multiplet at δ_H_ 3.27–3.53 ppm with the constant ^3^*J*_H,H_ ≈ 2.2 Hz, which indicates its equatorial position. Whereas for compounds **4a^tc^**, **4a–d^cc^**, which have the *cis*-configuration of the hexahydropyrimidine ring, the multiplet of the H(3) signal at δ_H_ 3.21–3.32 ppm contains two constants ^3^*J*_Hax,Hax_ = 10.4–10.8 Hz with the neighboring methylene protons, which indicates its axial position.

**Figure 4:**
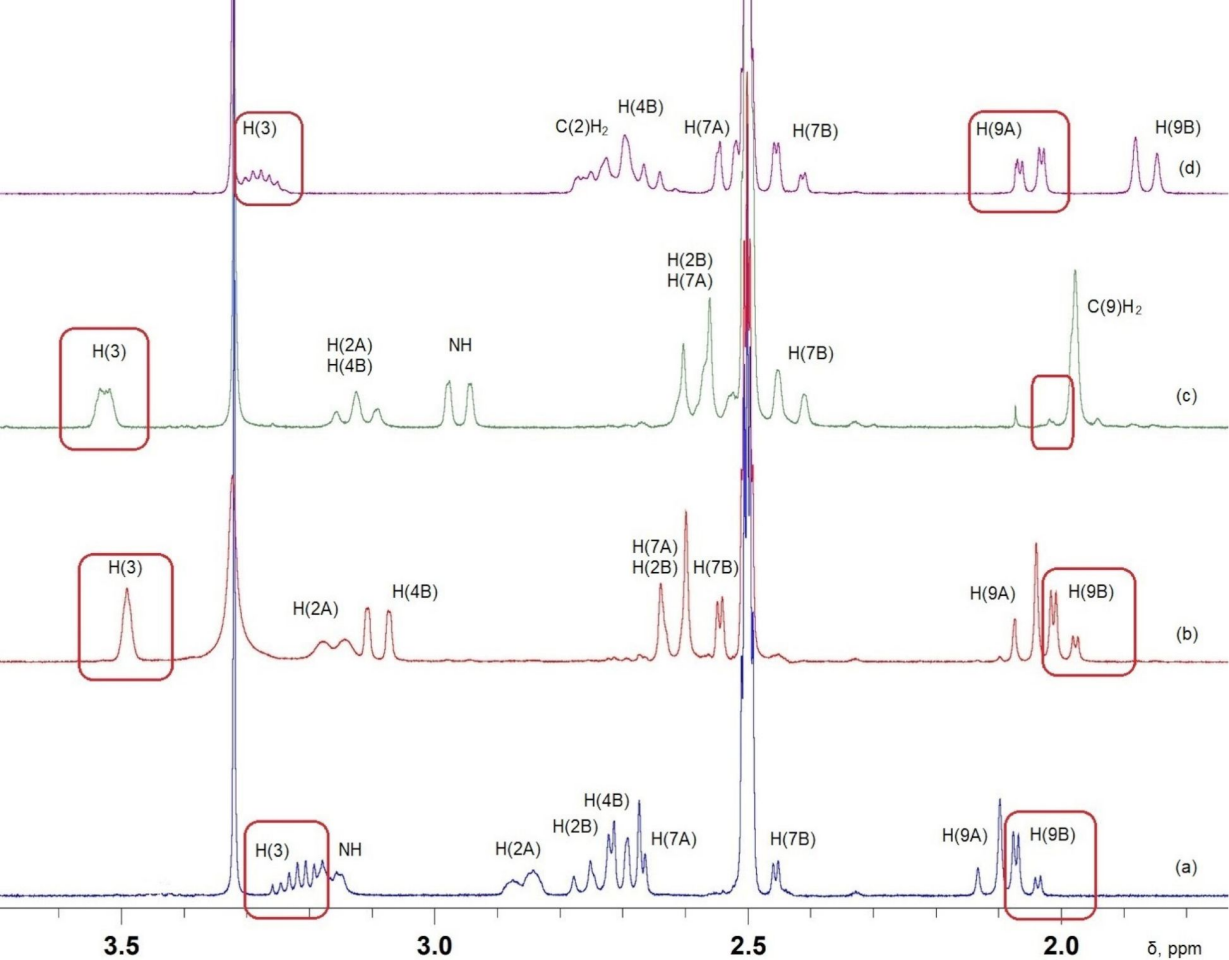
The fragments of the ^1^H NMR spectra (400 MHz, DMSO-*d*_6_) of diastereomers **4a^cc^** (a), **4а^ct^** (b), **4а^tt^** (c), **4a^tc^** (d).

Figure 4 exhibits the fragments of the ^1^H NMR spectra of diastereomers **4a^cc^**, **4a^ct^**, **4a^tt^** and **4a^tc^** clearly demonstrating the main differences.

An interesting feature was found in the ^1^H NMR spectra of diastereomers **4a–c**. In the compounds with the *trans-*configuration of the piperidone ring, the normal upfield-downfield relation of axial and equatorial shifts of methylene protons at C(9) is observed, i.e., the value of Δ_9ea_ = δ_eq_ – δ_ax_ is positive. With the *cis*-configuration of the piperidone ring, the chemical shifts of axial and equatorial shifts of methylene protons at C(9) are inverted and Δ_9ea_ < 0 (see Table 4, Figure 4).

**Table 4:** The difference in chemical shifts Δ_ea_ = δ_eq_ – δ_ax_ of diastereotopic protons at C(9) for compounds **4a–d**.

**No**	R	Δ_9ea_	**No**	R	Δ_9ea_	**No**	R	Δ_9ea_	**No**	R	Δ_9ea_

**4a** **^cc^**	Me	–0.06	**4a** **^tc^**	Me	+0.18	**4b** **^tc^**	Et	+0.30	**4d** **^cc^**	Ph	+0.25
**4a** **^ct^**	Me	–0.06	**4b** **^ct^**	Et	–0.12	**4c** **^cc^**	Bu	–0.12	**4d** **^ct^**	Ph	+0.29
**4a** **^tt^**	Me	+0.02	**4b** **^cc^**	Et	–0.12	**4c** **^ct^**	Bu	–0.12			

In the ^19^F NMR spectra, the CF_3_ group of diastereomeric octahydropyrido[1,2-*a*]pyrimidinones **4a^tt^** and **4a^tc^**, **4b^tc^** with the *trans*-configuration of the substituents in the piperidone ring shows upfield shifts (δ_F_ 79.50–79.61 ppm) compared with the *cis*-analogues **4a–d^cc^**, **4a–d^ct^** (δ_F_ 79.92–80.09 ppm).

**Figure 5:**
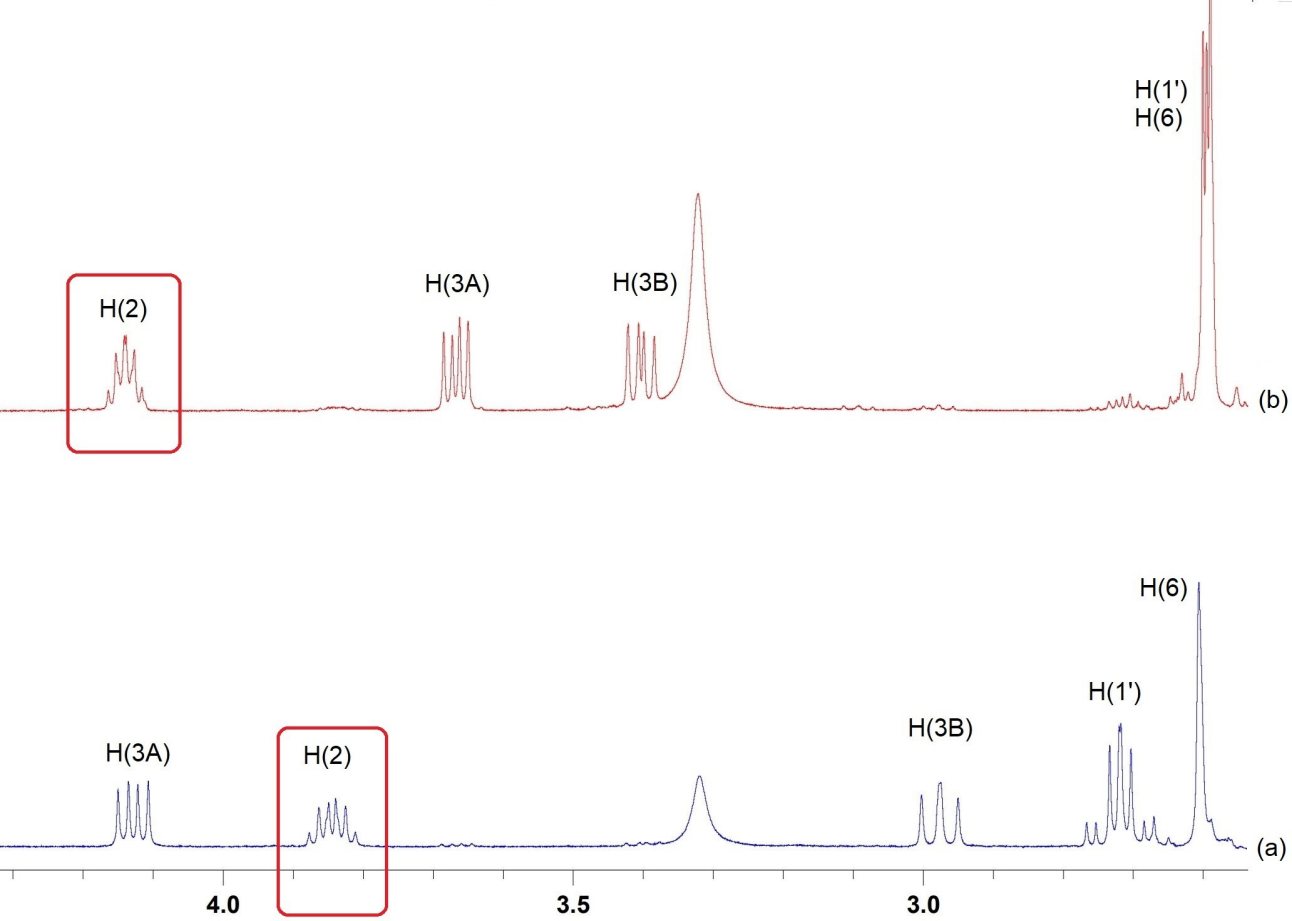
Fragments of ^1^H NMR spectra (400 MHz, DMSO-*d_6_*) of hexahydrooxazolo[3,2-*a*]pyridin-5-ones **5c^tc^** (a) and **5c^tt^** (b).

The configurations of diastereomers **5c^tc^** and **5c^tt^** were established using XRD (Figure 3). Figure 5 shows a fragment of the ^1^H NMR spectra of hexahydrooxazolo[3,2-*a*]pyridones **5c^tc^** (a) and **5c^tt^** (b) demonstrating the differences in the chemical shifts of the H(2) proton, which is located at the stereocenter, as well as other protons of the oxazolidine ring, which are affected by the change in configuration.

In the 2D NOESY spectra of compounds **5c^tc^** and **5c^tt^** (R = Bu) and the other hexahydrooxazolopyridones **5a**,**b** (R = Me, Et), similar correlations are observed, which allows us to determine their stereoconfiguration. For stereochemical assignments the key cross-peaks are: (OH, H(8A)), (H(8A), R) for the *trans-*configuration of the piperidone ring, (H(2), R) for the *trans-*configuration of the oxazolidine ring, and (H(1'), R) for the *cis*-configuration of the oxazolidine ring.

In the ^19^F NMR spectra, the signals of the CF_3_ group are recorded for hexahydrooxazolo[3,2-*a*]pyridones **5a–c^tc^** at δ_F_ 79.65–79.66 ppm and for **5a–c^tt^** at δ_F_ 79.73–79.74 ppm.

In this work, we investigated the biological properties of the synthesized compounds. We focused primarily on their potential antiviral activity, since a moderate anti-influenza effect of the related octahydropyrido[1,2-*a*]pyrimidinones was previously revealed [24]. The series of bicycles **4a^tt^**, **4a**–**d^ct^**, **4a^tc^**, **4a^cc^**, **5c^tc^** were tested against influenza A/Puerto Rico/8/34 (H1N1) virus on the MDCK cell line using ribavirin as a reference (Table 5).

**Table 5:** Antiviral activity of heterocycles **4** and **5** against influenza virus A/Puerto Rico/8/34 (H1N1) in MDCK cells.

entry	No	influenza A virus (MDCK cells)		

		CC_50_, μM	IC_50_, μM	SI

1	**4a** **^ct^**	>111	372	0
2	**4a** **^tt^**	>1118	86	**13**
3	**4a** **^tc^**	>1118	935	1
4	**4a** **^cc^**	>1118	>1118	1
5	**4b** **^ct^**	>1062	223	5
6	**4c** **^ct^**	>966	454	2
7	**4d** **^ct^**	>908	759	1
8	**5c** **^tc^**	>966	>966	1
ribavirin		>2130	36 ± 6	59

Of all the tested compounds, octahydropyridopyrimidinone **4a^tt^** (IC_50_ 86 μM, SI = 13) showed moderate antiviral activity. However, the value of selectivity index (SI) did not exceed that of reference compound ribavirin (Table 5, entry 2). In addition, this entire series of compounds is distinguished by the absence of cytotoxicity towards MDCK cells.

## Conclusion

To sum up, we have, for the first time, demonstrated the possibility of 1,3-diaminopropan-2-ol being involved in cyclization as an ambident *N,N-* and *N,O*-nucleophile. As a result, the three-component reaction of 1,3-diaminopropan-2-ol with ethyl trifluoroacetoacetate and methyl ketones leads to two types of heterocycles: octahydropyrido[1,2-*a*]pyrimidin-6-ones and hexahydrooxazolo[3,2-*a*]pyridin-5-ones. The cyclization direction is influenced by the substituent in the methyl ketone component, since, with acetone, the octahydropyrido[1,2-*a*]pyrimidinone formation predominates, and with the elongation of the alkyl substituent in methyl ketone both processes become competitive. However, the use of bifunctional AcOH/Et_3_N catalysis leads to an increase in the regioselectivity of octahydropyrido[1,2-*a*]pyrimidinones formation. In contrast, the cyclization with acetophenone occurs regiospecifically, yielding only octahydropyrido[1,2-*a*]pyrimidinones.

The presence of three chiral centers in the synthesized bicycles leads to the formation of hexahydrooxazolo[3,2-*a*]pyridones as two diastereomers, and octahydropyrido[1,2-*a*]pyrimidinones are formed as a mixture of two to four diastereomers. Diastereoselectivity correlates with the size of the alkyl substituent in the methyl ketone component. The tendency to increase the number of diastereomers of octahydropyrido[1,2-*a*]pyrimidinones is caused by the appearance of an additional asymmetric center in the starting 1,3-diaminopropan-2-ol, as opposed to the reactions with 1,3-diaminopropane [24]. The diastereoselectivity of cyclization with acetophenone depends on the reaction conditions: in the absence of a catalyst, the *cis,trans*-isomer is preferentially formed, whereas dual acid-base catalysis favors the formation of the other *cis,cis*-diastereomer.

The obtained hydrogenated oxazolo- and pyrimido-condensed pyridones are of interest for biological testing, which is confirmed by the discovery of an antiviral agent with moderate activity against the influenza virus A/Puerto Rico/8/34 (H1N1) among them.

## Supporting Information

File 1 General synthetic procedures, characterization data, XRD analysis data and copies of ^1^H, ^19^F, ^13^C NMR spectra, IR spectra, HRMS spectra of all synthesized compounds.

## Data Availability

All data that supports the findings of this study is available in the published article and/or the supporting information of this article.
