# Supplementary material for: Competitive cyclization of ethyl trifluoroacetoacetate and methyl ketones with 1,3-diamino-2-propanol into hydrogenated oxazolo- and pyrimido-condensed pyridones
Source: Beilstein J Org Chem. 2025 Dec 17;21:2716–29. doi: 10.3762/bjoc.21.209 (PMC12719880; doi:10.3762/bjoc.21.209)
Supplement: File 1 — General synthetic procedures, characterization data, XRD analysis data and copies of 1H, 19F, 13C NMR spectra, IR spectra, HRMS spectra of all synthesized compounds. [file Beilstein_J_Org_Chem-21-2716-s001.pdf]

BEILSTEIN JOURNAL OF ORGANIC CHEMISTRY

Supporting Information

for

Competitive cyclization of ethyl trifluoroacetoacetate and methyl ketones with 1,3-diamino-2-propanol into hydrogenated oxazolo- and pyrimido-condensed pyridones

Svetlana O. Kushch, Marina V. Goryaeva, Yanina V. Burgart, Marina A. Ezhikova, Mikhail I. Kodess, Pavel A. Slepukhin, Alexandrina S. Volobueva, Vladimir V. Zarubaev and Victor I. Saloutin

Beilstein J. Org. Chem. **2025,** *21,* 2716–2729. doi:10.3762/bjoc.21.209

General synthetic procedures, characterization data, XRD analysis data and copies of ^{1}H, ^{19}F, ^{13}C NMR spectra, IR spectra, HRMS spectra of all synthesized compounds

License and Terms: This is a supporting information file under the terms of the Creative Commons Attribution License (https://creativecommons.org/licenses/by/4.0). Please note that the reuse, redistribution and reproduction in particular requires that the author(s) and source are credited and that individual graphics may be subject to special legal provisions.
The license is subject to the *Beilstein Journal of Organic Chemistry* terms and conditions: (https://www.beilstein-journals.org/bjoc/terms)

Table of contents

Detailed experimental procedures and spectroscopic data for all synthesized compounds………………………………………………………………………...….. S2

X-ray analysis…………………………………………………………………….……... S11

Cytotoxicity and anti-viral activity evaluation…………………………………….. S20

References………………………………………………………………………… S21

Copies of ^{1}H, ^{19}F and ^{13}C NMR spectra ………………………………………….. S22

Copies of IR spectra …………………………………………………….………… S44

Copies of HRMS spectra…………………..……………………….……….. S52

Experimental

General remarks: Melting points were measured in open capillaries with a Stuart SMP3 melting-point apparatus. The IR spectra were recorded on a Perkin-Elmer Spectrum Two FT-IR spectrometer with diamond ATR accessory in the range of 400–4000 cm^{-1}. The ^{1}H, ^{19}F NMR spectra were registered on a Bruker DRX-400 spectrometer (400 or 376 MHz, respectively). The ^{13}C NMR spectra were recorded on a Bruker AVANCE III 500 spectrometer (126 MHz). The NMR spectra for all compounds were registered using the solvent DMSO-d_6. The internal standard was $SiMe_4$ for ^{1}H NMR spectra and C_6F_6 for ^{19}F. The ^{13}C chemical shifts were calibrated using the solvent signal DMSO-d_6 (δ_C 39.5 ppm). For all compounds the signals in the ^{1}H and ^{13}C spectra were assigned based on 2D ^{1}H-^{1}H COSY, ^{1}H-^{1}H NOESY, ^{1}H-^{13}C HSQC and HMBC experiments. High-resolution mass spectra (HRMS) were recorded on a Bruker maXis impact mass spectrometer (ESI). Column chromatography was performed on silica gel 60 (0.063–0.200 mm) (Macherey-Nagel). Ethyl 4,4,4-trifluoroacetoacetate (**1**), acetone (**2a**), 2-butanone (**2b**), 2-hexanone (**2c**), acetophenone (**2d**), 1,3-diaminopropan-2-ol (**3**) are commercially available (purchased from Alfa Aesar, Acros Organics, and Sigma-Aldrich).

General procedure for the synthesis of 4, 5

Procedure A: A 30-mL screw cap vial was charged with a mixture of ethyl trifluoroacetoacetate (**1**, 0.92 g, 5 mmol) and methyl ketone **2a–d** (5 mmol) in 1,4-dioxane (5 mL). Diaminopropanol **3** (0.45 g, 5 mmol) was then added, and the mixture was stirred at 60 °C for 24–48 h. The progress of reaction was monitored by TLC and ^{19}F NMR. If a solid precipitated (for **4a**,**b**,**d**^{ct}), it was filtered off and recrystallized from MeCN. Product **4a**^{tt} precipitated from the cold filtrate, was filtered and recrystallized from MeCN. If no precipitate was formed (for **4a**,**b**^{tc}, **4a–d**^{cc}, **4c**^{ct}, **5a–c**^{tc}, **5a–c**^{tt}), the reaction mixture was concentrated on a rotary evaporator, the residue was purified by column chromatography using gradient elution [eluent AcOEt, MeCN, MeCN:EtOH (4:1, 7:3, 1:1)].

Procedure B: A 30-mL screw cap vial was charged with a mixture of ethyl trifluoroacetoacetate (**1**, 0.92 g, 5 mmol) and methyl ketone **2a–d** (5 mmol) in 1,4-dioxane (5 mL). Diaminopropanol **3** (0.45 g, 5 mmol), acetic acid (0.6 g, 10 mmol) and triethylamine (1.01 g, 10 mmol) were then added, and the mixture was stirred at 60 °C for 24–48 h. The progress of reaction was monitored by TLC and ^{19}F NMR. The products **4a–c**^{ct}, **4a**^{tt}, **4a**,**b**^{tc}, **4a–c**^{cc}, **5c**^{tt} obtained from reactions with methyl ketones **2a–c** were isolated similarly to the procedure A. In the case of a reaction with acetophenone **2d** the

reaction mixture was concentrated on a rotary evaporator, the residue was washed with hot MeCN and recrystallized from EtOH to give product **4d**^{cc}. The filtrates were then combined and concentrated on a rotary evaporator. The residue was purified by column chromatography using gradient elution (eluent AcOEt, MeCN) to give product **4d**^{ct}.

(3R,8R*,9aR*)-3,8-Dihydroxy-9a-methyl-8-(trifluoromethyl)octahydro-6H-pyrido[1,2-a]pyrimidin-6-one (**4a**^{cc})*

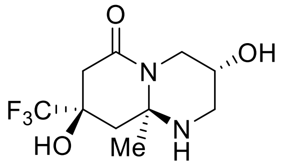

Yield 6% (0.085 g, A), 23% (0.31 g, B); white solid; m.p. 220–223°C (eluent MeCN). ^{1}H NMR δ: 1.49 (s, 3H, Me), 2.06 (dd, J = 14.1, 3.5 Hz, 1H, H-9B), 2.11 (d, J = 14.1 Hz, 1H, H-9A), 2.48 (dd, J = 16.2, 3.5 Hz, 1H, H-7B, overlapped with DMSO), 2.67–2.77 (m, 3H, H-7A, H-4A, H-2B), 2.86 (dm, J = 13.2 Hz, 1H, H-2A), 3.17 (dd, J = 10.7, 3.5 Hz, 1H, NH), 3.21 (tq, J = 10.7, 5.0 Hz, 1H, H-3), 4.55 (ddd, J = 12.6, 5.4, 1.8 Hz, 1H, H-4A), 5.08 (d, J = 5.0 Hz, 1H, HO-C^3), 7.08 (s, 1H, HO-C^8) ppm. ^{13}C NMR δ: 24.2 (Me), 37.7 (C-7), 37.9 (C-9), 42.6 (C-4), 46.8 (C-2), 64.3 (C-3), 69.3 (C-9a), 70.4 (q, J = 28.8 Hz, C-8), 125.0 (q, J = 285.2 Hz, CF_3), 163.9 (C-6) ppm. ^{19}F NMR δ: 79.91 (s, CF_3) ppm. IR v: 3429, 3278 (N–H, O–H), 2969–2872 (C–H), 1632 (C=O), 1452–1417 (C–N), 1170–1080 (C–F) см$^{-1}$. HRMS (ESI): *m/z* calcd. for $C_{10}H_{16}F_3N_2O_3$ [M + H]$^+$ 269.1108; found 269.1109.

(3S,8R*,9aR*)-3,8-Dihydroxy-9a-methyl-8-(trifluoromethyl)octahydro-6H-pyrido[1,2-a]pyrimidin-6-one (**4a**^{ct})*

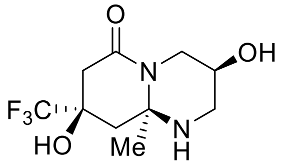

Yield 4% (0.055 g, A), 8% (0.11 g, B); white solid; m.p. 215°C (from MeCN). ^{1}H NMR δ: 1.46 (s, 3H, Me), 2.00 (dd, J = 14.0, 3.4 Hz, 1H, H-9B), 2.06 (d, J = 14.0 Hz, 1H, H-9A), 2.53 (dd, J = 16.6, 3.4 Hz, 1H, H-7B, overlapped with DMSO), 2.59–2.64 (m, 2H, H-7A, H-2B), 3.09 (dm, J = 13.8 Hz, 1H, H-4B), 3.16 (br.d, J = 14.2 Hz, 1H, H-2A), 3.49 (br.qw, J = 2.2 Hz, 1H, H-3), 4.51 (br.s, 1H, HO-C^3), 4.54 (dt, J = 13.8, 2.4 Hz, 1H, H-4A), 6.91 (br.s, 1H, HO-C^8) ppm. ^{13}C NMR δ: 24.2 (Me), 37.6 (C-7), 39.0 (C-9), 42.0 (C-4), 45.9 (C-2), 62.4 (C-3), 69.3 (C-9a), 70.3 (q, J = 28.6 Hz, C-8), 125.2 (q, J = 285.4 Hz, CF_3), 164.3 (C-6) ppm. ^{19}F NMR δ: 80.02 (s, CF_3) ppm. IR v: 3299, 3190 (N–H, O–H), 2994–2865

(C–H), 1625 (C=O), 1475–1412 (C–N), 1188–1064 (C–F) см$^{-1}$. HRMS (ESI): *m/z* calcd. for $C_{10}H_{16}F_3N_2O_3$ $[M + H]^+$ 269.1108; found 269.1108.

(3S,8R*,9aS*)-3,8-Dihydroxy-9a-methyl-8-(trifluoromethyl)octahydro-6H-pyrido[1,2-a]pyrimidin-6-one (**4a^{tt}**)*

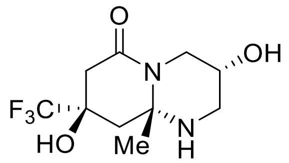

Yield 26% (0.349 g, A), 20% (0.269 g, B); white solid; m.p. 205–209°C (from MeCN). ^{1}H NMR *δ*: 1.56 (s, 3H, Me), 1.98 (AB-system, Δ_{AB} = 0.02, $^2J_{AB}$ = 14.0 Hz, $^4J_{9A,7B}$ = 2.6 Hz, 2H, H-9), 2.43 (dd, *J* = 16.8, 2.6 Hz, 1H, H-7B), 2.55 (dm, *J* = 14.4 Hz, 1H, H-2B), 2.58 (d, *J* = 16.8 Hz, 1H, H-7A), 2.59 (br.d, *J* = 13.7 Hz, 1H, NH), 2.96 (dd, *J* = 14.0, 2.0 Hz, 1H, H-4B), 3.13 (ddd, *J* = 14.3, 12.3, 2.2 Hz, 1H, H-2A), 3.53 (dqw, *J* = 6.4, 2.2 Hz, 1H, H-3), 4.42 (dt, *J* = 14.0, 2.4 Hz, 1H, H-4A), 4.70 (d, *J* = 6.4 Hz, 1H, HO-C^3), 6.34 (s, 1H, HO-C^8) ppm. ^{13}C NMR *δ*: 22.4 (Me), 37.1 (C-7), 39.1 (C-9), 41.6 (C-4), 44.2 (C-2), 62.8 (C-3), 69.4 (C-9a), 70.2 (q, *J* = 28.1 Hz, C-8), 125.5 (q, *J* = 286.5 Hz, CF_3), 164.0 (C-6) ppm. ^{19}F NMR *δ*: 79.52 (s, CF_3) ppm. IR ν: 3332, 3287 (N–H, O–H), 3023–2883 (C–H), 1593 (C=O), 1461–1411 (C–N), 1168–1080 (C–F) см$^{-1}$. HRMS (ESI): *m/z* calcd. for $C_{10}H_{16}F_3N_2O_3$ $[M + H]^+$ 269.1108; found 269.1110.

(3R,8R*,9aS*)-3,8-Dihydroxy-9a-methyl-8-(trifluoromethyl)octahydro-6H-pyrido[1,2-a]pyrimidin-6-one (**4a^{tc}**)*

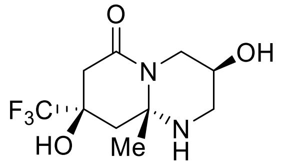

Yield 15% (0.202 g, A), 13% (0.175 g, B); white solid; m.p. 189–190°C (eluent MeCN). ^{1}H NMR *δ*: 1.55 (s, 3H, Me), 1.87 (d, *J* = 13.8 Hz, 1H, H-9B), 2.05 (dd, *J* = 13.8, 3.2 Hz, 1H, H-9A), 2.44 (dd, *J* = 16.9, 3.2 Hz, 1H, H-7B), 2.49–2.55 (m, 2H, H-7A, H-4B, overlapped with DMSO), 2.62–2.77 (m, 3H, H-2, NH), 3.28 (tqw, *J* = 10.4, 5.0 Hz, 1H, H-3), 4.54 (ddd, *J* = 12.8, 5.4, 1.7 Hz, 1H, H-4A), 5.03 (d, *J* = 5.0 Hz, 1H, HO-C^3), 6.35 (s, 1H, HO-C^8) ppm. ^{13}C NMR *δ*: 22.7 (Me), 36.9 (C-7), 38.9 (C-9), 42.0 (C-4), 45.4 (C-2), 64.8 (C-3), 68.9 (C-9a), 70.2 (q, *J* = 28.0 Hz, C-8), 125.5 (q, *J* = 286.4 Hz, CF_3), 163.0 (C-6) ppm. ^{19}F NMR *δ*: 79.61 (s, CF_3) ppm. IR ν: 3315, 3286 (N–H, O–H), 3003–2906 (C–H), 1623 (C=O), 1466–1412 (C–N), 1172–1069 (C–F) см$^{-1}$. HRMS (ESI): *m/z* calcd. for $C_{10}H_{16}F_3N_2O_3$ $[M + H]^+$ 269.1108; found 269.1109.

(3S,8R*,9aR*)-9a-Ethyl-3,8-dihydroxy-8-(trifluoromethyl)octahydro-6H-pyrido[1,2-a]pyrimidin-6-one (**4b^{ct}**)*

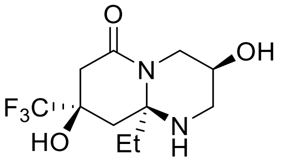

Yield 13% (0.184 g, A), 22% (0.315 g, B); white solid; m.p. 231–234°C (from MeCN). ^{1}H NMR *δ*: 0.75 (t, *J* = 7.3 Hz, 3H, H-2'), 1.75 (dq, *J* = 14.6, 7.3 Hz, 1H, H-1'B), 1.85 (dd, *J* = 14.2, 3.2 Hz, 1H, H-9B), 1.94 (dq, *J* = 14.6, 7.3 Hz, 1H, H-1'A), 1.96 (d, *J* = 14.2 Hz, 1H, H-9A), 2.44–2.51 (br.s, 1H, NH, overlapped with DMSO), 2.53 (dd, *J* = 16.5, 3.4 Hz, 1H, H-7B), 2.57 (d, *J* = 16.5 Hz, 1H, H-7A), 2.59 (dt, *J* = 14.4, 2.0 Hz, 1H, H-2B), 2.94 (dd, *J* = 14.0, 1.7 Hz, 1H, H-4B), 3.12 (br.d, *J* = 14.0 Hz, 1H, H-2A), 3.48 (m, 1H, H-3), 4.53 (dt, *J* = 14.0, 2.3 Hz, 1H, H-4A), 4.55 (br.s, 1H, OH-C^3), 6.89 (br.s, 1H, OH-C^8) ppm. ^{13}C NMR *δ*: 7.6 (C-2'), 26.6 (C-1$^{'t}$), 35.8 (C-9), 37.4 (C-7), 41.7 (C-4), 45.4 (C-2), 62.1 (C-3), 70.3 (q, *J* = 28.5 Hz, C-8), 71.8 (C-9a), 125.3 (q, *J* = 285.7 Hz, CF_3), 164.8 (C-6) ppm. ^{19}F NMR *δ*: 80.09 (s, CF_3) ppm. IR ν: 3298, 3208 (N–H, O–H), 2970–2916 (C–H), 1627 (C=O), 1476–1413 (C–N), 1183–1079 (C–F) cм$^{-1}$. HRMS (ESI): *m/z* calcd. for $C_{11}H_{18}F_3N_2O_3$ [M + H]$^+$ 283.1264; found 283.1266.

(3R,8R*,9aR*)-9a-Ethyl-3,8-dihydroxy-8-(trifluoromethyl)octahydro-6H-pyrido[1,2-a]pyrimidin-6-one (mixture of diastereomers **4b^{cc}** : **4b^{tc}** in the ratio 4 : 1)*

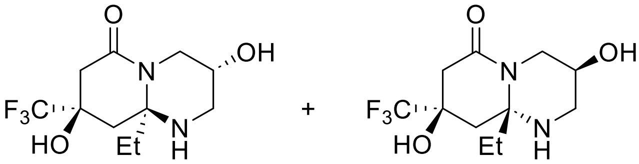

4b^{cc} : **4b^{tc}** in the ratio 4 : 1

Yield 21% (0.298 g, A), 32% (0.452 g, B); white solid; m.p. 219–222°C (eluent AcOEt). ^{1}H NMR *δ*: 0.75 (t, *J* = 7.3 Hz, 2.4H, H-2' **4b^{cc}**), 0.81 (t, *J* = 7.3 Hz, 0.6H, H-2' **4b^{tc}**), 1.54 (d, *J* = 14.3 Hz, 0.2H, H-9B **4b^{tc}**), 1.70 (dq, *J* = 14.6, 7.3 Hz, 0.8H, H-1'B **4b^{cc}**), 1.83–1.91 (m, 0.2H, H-1'B **4b^{tc}**), 1.89 (dd, *J* = 14.2, 3.6 Hz, 0.8H, H-9B **4b^{cc}**), 2.01 (d, 0.8H, *J* = 14.2 Hz, H-9A **4b^{cc}**), 2.04 (dq, *J* = 14.6, 7.3 Hz, 0.8H, H-1'A **4b^{cc}**), 2.17 (dd, *J* = 14.3, 2.9 Hz, 0.2H, H-9A **4b^{tc}**), 2.24 (dq, *J* = 14.5, 7.3 Hz, 0.2H, H-1'A **4b^{tc}**), 2.45–2.61 (m, 2.6H, H-4B, H-7B **4b^{cc}**, H-7 **4b^{tc}**, H-2B **4b^{tc}**, NH **4b^{tc}**, part. overlapped with DMSO), 2.65 (d, *J* = 16.1, 0.8H, H-7A **4b^{cc}**), 2.68–2.73 (m, 1H, H-2B **4b^{cc}**, H-2A **4b^{tc}**), 2.85 (br.d, *J* = 13.0 Hz, 0.8H, H-2A **4b^{cc}**), 3.11 (m, 0.8H, NH **4b^{cc}**), 3.21 (tq, *J* = 10.6, 5.1 Hz, 0.8H, H-3 **4b^{cc}**), 3.27–3.34 (m, 0.2H, H-3 **4b^{tc}**, part. overlapped with H_2O), 4.52–4.56 (m, 0.2H, H-4A **4b^{tc}**), 4.56 (ddd, *J* = 12.6, 5.2, 1.4 Hz, 0.8H, H-4A **4b^{cc}**), 5.00 (d, *J* = 4.9 Hz, 0.2H, HO-C^3 **4b^{tc}**), 5.06

(d, J = 5.1 Hz, 0.8H, HO-C^3 **4b^{cc}**), 6.30 (s, 0.2H, HO-C^8 **4b^{tc}**), 7.04 (s, 0.8H, HO-C^8 **4b^{cc}**). ^{13}C NMR δ: 7.4 (C-2', **4b^{cc}**), 8.4 (C-2', **4b^{tc}**), 23.6 (C-1', **4b^{tc}**), 26.6 (C-1', **4b^{cc}**), 33.1 (C-9, **4b^{tc}**), 34.7 (C-9, **4b^{cc}**), 37.1 (C-7, **4b^{tc}**), 37.5 (C-7, **4b^{cc}**), 41.9 (C-4, **4b^{tc}**), 42.3 (C-4, **4b^{cc}**), 45.0 (C-2, **4b^{tc}**), 46.4 (C-2, **4b^{cc}**), 63.8 (C-3, **4b^{cc}**), 64.4 (C-3, **4b^{tc}**), 70.1 (q, J = 28.1 Hz, C-8, **4b^{tc}**), 70.4 (q, J = 28.8 Hz, C-8, **4b^{cc}**), 71.6 (C-9a, **4b^{tc}**), 71.8 (C-9a, **4b^{cc}**), 125.1 (q, J = 285.3 Hz, CF_3, **4b^{cc}**), 125.6 (q, J = 286.4 Hz, CF_3, **4b^{tc}**), 163.5 (C-6, **4b^{tc}**), 164.3 (C-6, **4b^{cc}**) ppm. ^{19}F NMR δ: 79.50 (s, 0.6F, CF_3, **4b^{tc}**), 80.00 (s, 2.4F, CF_3, **4b^{cc}**) ppm. IR ν: 3435, 3278 (N–H, O–H), 2981–2894 (C–H), 1633 (C=O), 1450–1416 (C–N), 1180–1095 (C–F) см$^{-1}$. HRMS (ESI): *m/z* calcd. for $C_{11}H_{18}F_3N_2O_3$ $[M + H]^+$ 283.1264; found 283.1267.

(3R,8R*,9aR*)-9a-Butyl-3,8-dihydroxy-8-(trifluoromethyl)octahydro-6H-pyrido[1,2-a]pyrimidin-6-one (**4c^{cc}**)*

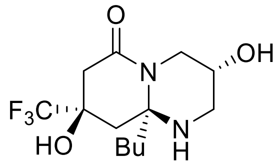

Yield 8% (0.125 g, A), 21% (0.326 g, B); white solid; m.p. 184–186°C (eluent AcOEt). ^{1}H NMR δ: 0.89 (t, J = 7.2 Hz, 3H, H-4'), 0.96–1.05 (m, 1H, H-2'B), 1.19–1.27 (m, 1H, H-2'A), 1.31 (sex, J = 7.2 Hz, 2H, H-3'), 1.66 (ddd, J = 14.0, 12.3, 3.8 Hz, 1H, H-1'B), 1.92 (dd, J = 14.1, 3.5 Hz, 1H, H-9B), 2.01 (ddd, J = 14.0, 11.9, 4.9 Hz, 1H, H-1'A), 2.04 (d, J = 14.1 Hz, 1H, H-9A), 2.48 (dd, J = 16.1, 3.5 Hz, 1H, H-7B), 2.54 (dd, J = 12.6, 11.0 Hz, 1H, H-4B), 2.65 (d, J = 16.1 Hz, 1H, H-7A), 2.71 (dt, J = 13.1, 10.8 Hz 1H, H-2B), 2.85 (dm, J = 13.1 Hz, 1H, H-2A), 3.11 (dd, J = 10.8, 3.6 Hz, 1H, NH), 3.21 (tq, J = 10.8, 5.1 Hz, 1H, H-3), 4.56 (ddd, J = 12.6, 5.3, 1.5 Hz, 1H, H-4A), 5.06 (d, J = 5.1 Hz, 1H, HO-C^3), 7.04 (s, 1H, HO-C^8) ppm. ^{13}C NMR δ: 13.9 (C-4'), 22.1 (C-3'), 24.9 (C-2'), 33.7 (C-1'), 35.2 (C-9), 37.5 (C-7), 42.4 (C-4), 46.4 (C-2), 63.8 (C-3), 70.4 (q, J = 28.8 Hz, C-8), 71.4 (C-9a), 125.0 (q, J = 285.3 Hz, CF_3), 164.2 (C-6) ppm. ^{19}F NMR δ: 80.00 (s, CF_3) ppm. IR ν: 3309, 3267, 3189 (N–H, O–H), 2964–2873 (C–H), 1626 (C=O), 1475–1412 (C–N), 1171–1084 (C–F) см$^{-1}$. HRMS (ESI): *m/z* calcd. for $C_{13}H_{22}F_3N_2O_3$ $[M + H]^+$ 311.1577; found 311.1578.

(3S,8R*,9aR*)-9a-Butyl-3,8-dihydroxy-8-(trifluoromethyl)octahydro-6H-pyrido[1,2-a]pyrimidin-6-one (**4c**^{ct})*

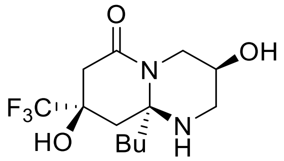

Yield 13% (0.202 g, A), 18% (0.28 g, B); white solid; m.p. 174–177°C (eluent MeCN). ^{1}H NMR *δ*: 0.88 (t, *J* = 7.2 Hz, 3H, H-4'), 0.96–1.05 (m, 1H, H-2'B), 1.19–1.27 (m, 2H, H-2'A), 1.31 (sex, *J* = 7.2 Hz, 2H, H-3'), 1.71 (ddd, *J* = 13.9, 12.3, 3.9 Hz, 1H, H-1'B), 1.87 (dd, *J* = 14.1, 3.3 Hz, 1H, H-9B), 1.90 (ddd, *J* = 13.9, 11.8, 4.7 Hz, 1H, H-1'A), 2.00 (d, *J* = 14.1 Hz, 1H, H-9A), 2.52 (dd, *J* = 16.3, 3.3 Hz, 1H, H-7B, overlapped with DMSO), 2.57 (d, *J* = 16.3 Hz, 1H, H-7A), 2.59 (dq, *J* = 14.1, 2.5 Hz, 1H, H-2B), 2.96 (dd, *J* = 13.9, 1.4 Hz, 1H, H-4B), 3.13 (ddd, *J* = 14.1, 12.0, 2.1 Hz, 1H, H-2A), 3.25 (dd, *J* = 11.9, 3.2 Hz, 1H, NH), 3.49 (br.sex, *J* = 2.5 Hz, 1H, H-3), 4.51 (d, *J* = 2.5 Hz, 1H, HO-C^{3}), 4.54 (dt, *J* = 13.9, 2.4 Hz, 1H, H-4A), 6.90 (s, 1H, HO-C^{8}) ppm. ^{13}C NMR *δ*: 14.0 (C-4'), 22.2 (C-3'), 25.2 (C-2'), 33.8 (C-1'), 36.4 (C-9), 37.5 (C-7), 41.8 (C-4), 45.4 (C-2), 62.1 (C-3), 70.3 (q, *J* = 28.3 Hz, C-8), 71.5 (C-9a), 125.3 (q, *J* = 285.6 Hz, CF_3), 164.7 (C-6) ppm. ^{19}F NMR *δ*: 80.09 (s, CF_3) ppm. IR ν: 3302, 3126 (N–H, O–H), 2938–2852 (C–H), 1629 (C=O), 1485–1423 (C–N), 1192–1079 (C–F) см$^{-1}$. HRMS (ESI): *m/z* calcd. for $C_{13}H_{22}F_3N_2O_3$ [M + H]$^{+}$ 311.1577; found 311.1579.

(3R,8R*,9aR*)-3,8-Dihydroxy-9a-phenyl-8-(trifluoromethyl)octahydro-6H-pyrido[1,2-a]pyrimidin-6-one (**4d**^{cc})*

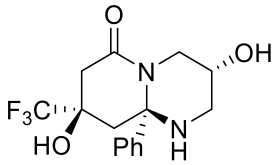

Yield 5% (0.083 g, A), 54% (0.892 g, B); white solid; m.p. 253–255°C (eluent AcOEt (A); from EtOH (B)). ^{1}H NMR *δ*: 1.91 (d, *J* = 14.2 Hz, 1H, H-9B), 2.16 (dd, *J* = 14.2, 3.6 Hz, 1H, H-9A), 2.25 (dt, *J* = 12.4, 11.0 Hz, 1H, H-4B), 2.38 (dt, *J* = 12.8, 11.0 Hz, 1H, H-2B), 2.63 (dd, *J* = 16.1, 3.6 Hz, 1H, H-7B), 2.93 (d, *J* = 12.8 Hz, 1H, H-2A), 2.97 (d, *J* = 16.1 Hz, 1H, H-7A), 3.32 (tq, *J* = 10.4, 5.1 Hz, 1H, H-3, overlapped with H_2O), 3.71 (dd, *J* = 11.7, 3.8 Hz, 1H, NH), 4.55 (ddd, *J* = 12.4, 5.1, 2.0 Hz, 1H, H-4A), 4.96 (d, *J* = 5.1 Hz, 1H, HO-C^{3}), 7.20 (s, 1H, HO-C^{8}), 7.33 (tt, *J* = 7.2, 1.5 Hz, 1H, H*p*), 7.42–7.50 (m, 4H, H*o*, H*m*) ppm. ^{13}C NMR *δ*: 37.6 (C-6), 41.2 (C-9), 44.4 (C-4), 47.6 (C-2), 63.9 (C-3), 70.9 (q, *J* = 29.0 Hz, C-8), 75.2 (C-9a), 124.8 (q, *J* = 285.5 Hz, CF_3), 126.2 (C*o*), 127.5 (C*p*), 129.1 (C*m*), 142.7 (C*i*), 165.2 (C-6) ppm. ^{19}F NMR *δ*: 79.94 (s, CF_3) ppm. IR ν: 3335, 3280 (N–

H, O–H), 2950 (C–H), 1612 (C=O), 1462–1414 (C–N), 1170–1119 (C–F) см$^{-1}$. HRMS (ESI): *m/z* calcd. for $C_{15}H_{18}F_3N_2O_3$ [M + H]$^+$ 331.1264; found 331.1266.

(3S,8R*,9aR*)-3,8-Dihydroxy-9a-phenyl-8-(trifluoromethyl)octahydro-6H-pyrido[1,2-a]pyrimidin-6-one (**4d^{ct}**)*

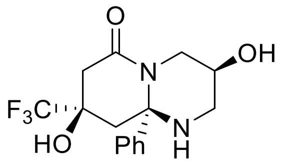

Yield 59% (0.975 g, A), 6% (0.102 g, B); white solid; m.p. 244–246°C (from MeCN (A), eluent MeCN (B)). ^{1}H NMR *δ*: 1.83 (d, *J* = 14.1 Hz, 1H, H-9B), 2.12 (dd, *J* = 14.1, 3.6 Hz, 1H, H-9A), 2.64 (dd, *J* = 13.5, 1.6 Hz, 1H, H-4B), 2.66–2.71 (m, 2H, H-7B, H-2B), 2.75 (ddd, *J* = 14.2, 12.0, 1.9 Hz, 1H, H-2A), 2.85 (d, *J* = 16.5 Hz, 1H, H-7A), 3.35 (m, 1H, H-3, part. overlapped with H_2O), 3.91 (dd, *J* = 12.0, 3.6 Hz, 1H, NH), 4.62 (dt, *J* = 13.5, 2.4 Hz, 1H, H-4A), 4.70 (br.d, *J* = 2.8 Hz, 1H, HO-C^3), 7.11 (s, 1H, HO-C^8), 7.32 (tt, *J* = 7.1, 1.6 Hz, 1H, H*p*), 7.41–7.47 (m, 4H, H*o*, H*m*) ppm. ^{13}C NMR *δ*: 37.6 (C-7), 42.3 (C-9), 43.6 (C-4), 46.7 (C-2), 62.1 (C-3), 70.7 (q, *J* = 28.6 Hz, C-8), 75.2 (C-9a), 125.0 (q, *J* = 286.0 Hz, CF_3), 126.1 (C*o*), 127.4 (C*p*), 128.9 (C*m*), 142.9 (C*i*), 165.8 (C-6) ppm. ^{19}F NMR *δ*: 80.03 (s, CF_3) ppm. IR ν: 3332, 3136 (N–H, O–H), 2964–2933 (C–H), 1630 (C=O), 1471–1411 (C–N), 1162–1080 (C–F) см$^{-1}$. HRMS (ESI): *m/z* calcd. for $C_{15}H_{18}F_3N_2O_3$ [M + H]$^+$ 331.1264; found 331.1265.

*2-(Aminomethyl)-7-hydroxy-8a-methyl-7-(trifluoromethyl)hexahydro-5H-oxazolo[3,2-a]pyridin-5-one (mixture of diastereomers **5a^{tc}** : **5a^{tt}** in the ratio 11 : 9)*

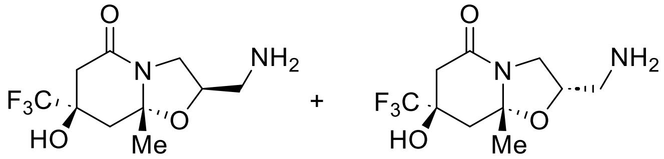

5a^{tc} : **5a^{tt}** in the ratio 11 : 9

Yield 14% (0.188 g, A); white solid; m.p. 155–157°C (eluent MeCN : EtOH (1:1)). ^{1}H NMR *δ*: 1.30–1.49 (br.s, 2H, NH_2), 1.51 (s, 3H, Me), 1.87 (br.d, *J* = 13.4 Hz, 1H, H-8B), 2.24 (d, *J* = 13.5 Hz, 0.55H, H-8A **5a^{tc}**), 2.32 (d, *J* = 13.5 Hz, 0.45H, H-8A **5a^{tt}**), 2.53–2.65 (m, 2.9H, H-1' **5a^{tt}**), H-6), 2.71 (ABX system, Δ_{AB} = 0.03 ppm, J_{AB} = 13.1, J_{BX} = 5.6, J_{AX} = 5.1 Hz, 1.1H, H-1' **5a^{tc}**), 3.08 (dd, *J* = 11.3, 9.4 Hz, 0.55H, H-3B **5a^{tc}**), 3.40 (dd, *J* = 11.3, 7.6 Hz, 0.45H, H-3B **5a^{tt}**), 3.70 (dd, *J* = 11.3, 6.0 Hz, 0.45H, H-3A **5a^{tt}**), 3.84 dq, 0.55H, H-2 **5a^{tc}**), 4.17–4.23 (m, 1H, H-3A **5a^{tc}**, H-2 **5a^{tt}**), 6.52 (s, 1H, OH). ^{13}C NMR *δ*: 25.0 (Me, **5a^{tt}**), 27.6 (Me, **5a^{tc}**), 37.2 (C-6, **5a^{tt}**), 37.5 (C-6, **5a^{tc}**), 38.8 (C-8, **5a^{tt}**), 39.6 (C-8, **5a^{tc}**),

44.2 (C-1', **5a**^{tc}), 44.3 (C-3, **5a**^{tt}), 44.5 (C-1', **5a**^{tt}), 44.7 (C-3, **5a**^{tc}), 69.9 (q, J = 28.6 Hz, C-7, **5a**^{tc}), 70.6 (q, J = 28.6 Hz, C-7, **5a**^{tt}), 75.3 (C-2, **5a**^{tt}), 76.8 (C-2, **5a**^{tc}), 91.1 (C-8a, **5a**^{tc}), 91.3 (C-8a, **5a**^{tt}), 125.6 (q, J = 286.0 Hz, CF_3, **5a**^{tt}), 125.8 (q, J = 286.0 Hz, CF_3, **5a**^{tc}), 163.2 (C-5, **5a**^{tt}), 163.3 (C-5, **5a**^{tc}) ppm. ^{19}F NMR δ: 79.66 (s, 1.7F, CF_3, **5a**^{tc}), 79.74 (s, 1.3F, CF_3, **5a**^{tt}) ppm. IR ν: 3352, 3296 (NH_2), 2614 (O–H), 2985–2862 (C–H), 1628 (C=O), 1471–1444 (C–N), 1184–1125 (C–F) см$^{-1}$. HRMS (ESI): *m/z* calcd. for $C_{10}H_{16}F_3N_2O_3$ [M + H]$^+$ 269.1108; found 269.1107.

(2R,7R*,8aR*)-2-(Aminomethyl)-8a-ethyl-7-hydroxy-7-(trifluoromethyl)hexahydro-5H-oxazolo[3,2-a]pyridin-5-one (**5b**^{tc})*

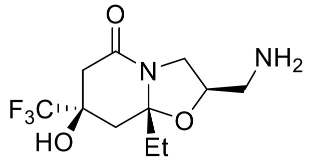

Yield 16% (0.228 g, A); white solid; m.p. 158–160°C (eluent MeCN : EtOH (4:1)). ^{1}H NMR δ: 0.88 (t, J = 7.4 Hz, 3H, H-2"), 1.43 (br.s, 2H, NH_2), 1.78 (d, J = 14.1 Hz, 1H, H-8B), 1.82 (q, J = 7.4 Hz, 2H, H-1"), 2.28 (d, J = 14.1 Hz, 1H, H-8A), 2.61 (AB-system, Δ_{AB} = 0.02 ppm, J_{AB} = 17.3 Hz, 2H, H-6), 2.69 (dd, J = 13.1, 5.6 Hz, 1H, H-1'B), 2.74 (dd, J = 13.1, 5.3 Hz, 1H, H-1'A), 2.98 (dd, J = 11.2, 9.7 Hz, 1H, H-3B), 3.84 (dq, J = 9.7, 5.6 Hz, 1H, H-2), 4.13 (dd, J = 11.2, 5.9 Hz, 1H, H-3A), 6.53 (s, 1H, OH) ppm. ^{13}C NMR δ: 7.6 (C-2"), 31.2 (C-1"), 38.07 and 38.09 (C-6 and C-8), 43.7 (C-1'), 45.9 (C-3), 69.8 (q, J = 28.6 Hz, C-7), 76.4 (C-2), 93.5 (C-8a), 126.0 (q, J = 286.1 Hz, CF_3), 163.9 (C-5) ppm. ^{19}F NMR δ: 79.65 (s, CF_3) ppm. IR ν: 3354, 3296 (NH_2), 2976–2877 (C–H), 2583 (O–H), 1656 (C=O), 1432–1420 (C–N), 1185–1105 (C–F) см$^{-1}$. HRMS (ESI): *m/z* calcd. for $C_{11}H_{18}F_3N_2O_3$ [M + H]$^+$ 283.1264; found 283.1266.

(2S,7R*,8aR*)-2-(Aminomethyl)-8a-ethyl-7-hydroxy-7-(trifluoromethyl)hexahydro-5H-oxazolo[3,2-a]pyridin-5-one (**5b**^{tt})*

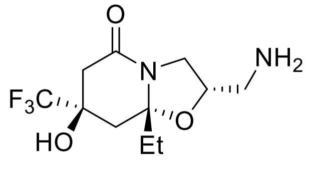

Yield 18% (0.255 g, A); white solid; m.p. 158–160°C (eluent MeCN : EtOH (4:1)). ^{1}H NMR δ: 0.88 (t, J = 7.2 Hz, 3H, H-2"), 1.59 (br.s, 2H, NH_2), 1.67 (d, J = 13.7 Hz, 1H, H-8B), 1.77–1.91 (m, 2H, H-1"), 2.41 (d, J = 13.7 Hz, 1H, H-8A), 2.55–2.63 (m, 4H, H-1', H-6), 3.40 (dd, J = 11.3, 7.4 Hz, 1H, H-3B), 3.67 (dd, J = 11.3, 6.1 Hz, 1H, H-3A), 4.14 (dq, J = 7.4, 5.6 Hz, 1H, H-2), 6.49 (s, 1H, OH) ppm. ^{13}C NMR δ: 8.1 (C-2"), 28.7 (C-1"), 35.6 (C-

8), 37.6 (C-6), 44.5 (C-1'), 44.9 (C-3), 70.6 (q, *J* = 28.5 Hz, C-7), 75.6 (C-2), 93.6 (C-8a), 125.8 (q, *J* = 286.1 Hz, CF_3), 163.4 (C-5) ppm. ^{19}F NMR *δ*: 79.73 (s, CF_3) ppm. IR ν: 3373, 3303 (NH_2), 2978–2865 (C–H), 2590 (O–H), 1658 (C=O), 1449–1416 (C–N), 1192–1131 (C–F) см$^{-1}$. HRMS (ESI): *m/z* calcd. for $C_{11}H_{18}F_3N_2O_3$ [M + H]$^+$ 283.1264; found 283.1266.

(2S,7R*,8aR*)-2-(Aminomethyl)-8a-butyl-7-hydroxy-7-(trifluoromethyl)hexahydro-5H-oxazolo[3,2-a]pyridin-5-one (**5c^{tc}**)*

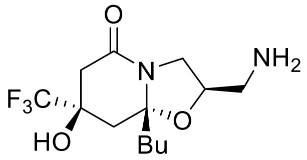

Yield 17% (0.264 g, A); white solid; m.p. 107–110°C (eluent MeCN). ^{1}H NMR *δ*: 0.88 (t, *J* = 7.1 Hz, 3H, H-4"), 1.22–1.32 (m, 3H, H-2"B, H-3"), 1.34–1.49 (m, 3H, H-2"A, NH_2), 1.74–1.85 (m, 3H, H-1", H-8B), 2.27 (d, *J* = 14.0 Hz, 1H, H-8A), 2.59 (d, *J* =17.1 Hz, 1H, H-6B), 2.63 (d, *J* = 17.1 Hz, 1H, H-6A), 2.69 (dd, *J* = 13.1, 5.6 Hz, 1H, H-1'B), 2.74 (dd, *J* = 13.1, 5.2 Hz, 1H, H-1'A), 2.98 (dd, *J* = 11.2, 9.7 Hz, 1H, H-3B), 3.84 (dq, *J* = 9.7, 5.5 Hz, 1H, H-2), 4.12 (dd, *J* = 11.2, 6.0 Hz, 1H, H-3A), 6.52 (s, 1H, OH) ppm. ^{13}C NMR *δ*: 13.8 (C-4"), 22.1 (C-3"), 25.0 (C-2"), 38.0 (C-1"), 38.1 (C-6), 38.5 (C-8), 43.7 (C-1'), 45.9 (C-3), 69.8 (q, *J* = 28.5 Hz, C-7), 76.4 (C-2), 93.3 (C-8a), 126.0 (q, *J* = 285.9 Hz, CF_3), 163.9 (C-5) ppm. ^{19}F NMR *δ*: 79.65 (s, CF_3) ppm. IR ν: 3376, 3272, (NH_2), 2989–2868 (C–H), 2601 (O–H), 1652 (C=O), 1466–1398 (C–N), 1159–1124 (C–F) см$^{-1}$. HRMS (ESI): *m/z* calcd. for $C_{13}H_{22}F_3N_2O_3$ [M + H]$^+$ 311.1577; found 311.1575.

(2R,7R*,8aR*)-2-(Aminomethyl)-8a-butyl-7-hydroxy-7-(trifluoromethyl)hexahydro-5H-oxazolo[3,2-a]pyridin-5-one (**5c^{tt}**)*

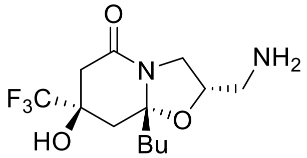

Yield 19% (0.295 g, A), 3% (0.047 g, B); white solid; m.p. 159–162°C (eluent MeCN : EtOH (7:3)). ^{1}H NMR *δ*: 0.88 (t, *J* = 7.1 Hz, 3H, H-4"), 1.23–1.39 (m, 4H, H-2", H-3"), 1.42 (br.s, 2H, NH_2), 1.68 (d, *J* = 13.6, 1.0 Hz, 1H, H-8B), 1.73–1.78 (m, 1H, H-1"B), 1.85–1.91 (m, 1H, H-1"A), 2.41 (d, *J* = 13.6 Hz, 1H, H-8A), 2.55–2.63 (m, 4H, H-1', H-6), 3.41 (dd, *J* = 11.3, 7.4 Hz, 1H, H-3B), 3.66 (dd, *J* = 11.3, 6.1 Hz, 1H, H-3A), 4.14 (dq, *J* = 7.4, 5.5 Hz, 1H, H-2), 6.49 (s, 1H, OH) ppm. ^{13}C NMR *δ*: 13.8 (C-4"), 22.1 (C-3"), 25.5 (C-2"), 35.7 (C-1"), 36.3 (C-8), 37.6 (C-6), 44.5 (C-1'), 44.9 (C-3), 70.6 (q, *J* = 28.7 Hz, C-7), 75.6

(C-2), 93.3 (C-8a), 125.8 (q, J = 286.1 Hz, CF_3), 163.4 (C-5) ppm. ^{19}F NMR δ: 79.74 (s, CF_3) ppm. IR ν: 3371, 3304 (NH_2), 2960–2871 (C–H), 2602 (O–H), 1652 (C=O), 1454–1423 (C–N), 1218–1136 (C–F) $см^{-1}$. HRMS (ESI): *m/z* calcd. for $C_{13}H_{22}F_3N_2O_3$ $[M + H]^+$ 311.1577; found 311.1575.

X-ray analysis

The XRD analyses for compounds **4a**^{cc}, **4a**^{ct}, **4a**^{tt}, **4a**^{tc}, **4d**^{ct}, **5c**^{tc} and **5c**^{tt} were carried out using equipment of the Center for Joint Use "Spectroscopy and Analysis of Organic Compounds" at the Postovsky Institute of Organic Synthesis of the Russian Academy of Sciences (Ural Branch). The experiment was accomplished on the automated X-ray diffractometer «Xcalibur 3» with CCD detector on standard procedure (MoK_α-irradiation, graphite monochromator, ω-scans with 1° step at T = 295(2) К). Empirical absorption correction was applied. The solution and refinement of the structures were accomplished with using Olex2 program package [1]. The structures were solved by the method of the intrinsic phases in the ShelXT program and refined by ShelXL by full-matrix least-squares method for non-hydrogen atoms [2,3]. The H-atoms at C–H bonds were placed in the calculated positions, H-atoms of the O–H and N–H bonds were solved by direct methods and were refined independently in isotropic approximation.

The atomic coordinates and other structural parameters for compounds **4a**^{cc}, **4a**^{ct}, **4a**^{tt}, **4a**^{tc}, **4d**^{ct}, **5c**^{tc} and **5c**^{tt} were deposited with the Cambridge Crystallographic Data Centre (CCDC 2479553 (**4a**^{cc}), CCDC 2479554 (**4a**^{ct}), CCDC 2479555 (**4a**^{tt}), CCDC 2479556 (**4a**^{tc}), CCDC 2479557 (**4d**^{ct}), CCDC 2479558 (**5c**^{tc}), CCDC 2479559 (**5c**^{tt})).

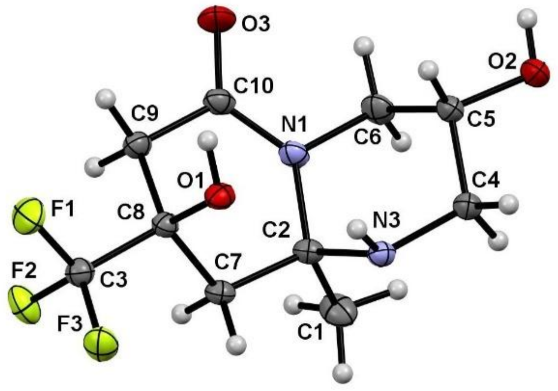

(**a**)

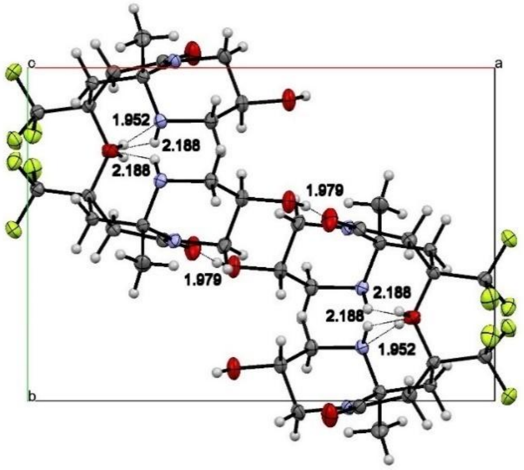

(**b**)

Figure S1: ORTEP view of compound **4a^{cc}** (**a**) and crystal packing (**b**) showing the thermal ellipsoids at 50% probability (CCDC 2479553).

**Table S1: Crystal data and structure refinement for 4a^{cc}**	
Empirical formula	$C_{10}H_{15}F_3N_2O_3$
Formula weight	268.24
Temperature/K	295(2)
Crystal system	monoclinic
Space group	$P2_1/c$
a/Å	13.4535(4)
b/Å	9.4450(3)
c/Å	9.2482(3)
α/°	90
β/°	100.968(3)
γ/°	90
Volume/Å^3	1153.69(6)
Z	4
ρ_{calc}g/cm^3	1.544
μ/mm^{-1}	0.144
F(000)	560.0
Crystal size/mm^3	0.46 × 0.34 × 0.19
Radiation	MoKα (λ = 0.71073)
2Θ range for data collection/°	5.302 to 62.064
Index ranges	-19 ≤ h ≤ 16, -12 ≤ k ≤ 11, -12 ≤ l ≤ 8
Reflections collected	5454
Independent reflections	3175 [R_{int} = 0.0139, R_{sigma} = 0.0284]
Data/restraints/parameters	3175/0/180
Goodness-of-fit on F^2	1.029
Final R indexes [I>=2σ (I)]	R_1 = 0.0402, wR_2 = 0.1002
Final R indexes [all data]	R_1 = 0.0581, wR_2 = 0.1120
Largest diff. peak/hole / e Å^{-3}	0.20/-0.21

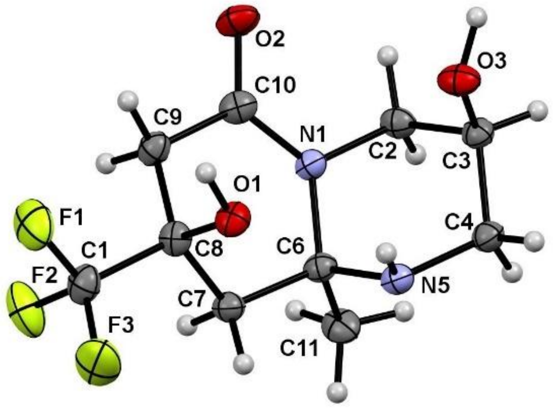

(**a**)

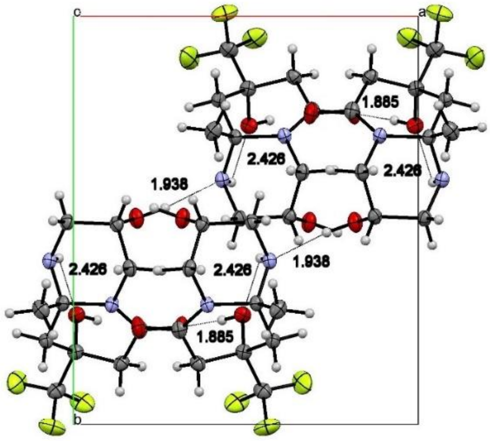

(**b**)

Figure S2: ORTEP view of compound **4a**$^{\mathbf{ct}}$ (**a**) and crystal packing (**b**) showing the thermal ellipsoids at 50% probability (CCDC 2479554).

**Table S2: Crystal data and structure refinement for 4a**$^{\mathbf{ct}}$	
Empirical formula	$C_{10}H_{15}F_3N_2O_3$
Formula weight	268.24
Temperature/K	295(2)
Crystal system	orthorhombic
Space group	$Pca2_1$
a/Å	10.4313(6)
b/Å	12.3501(15)
c/Å	8.8705(7)
α/°	90.00
β/°	90.00
γ/°	90.00
Volume/$Å^3$	1142.77(17)
Z	4
ρ_{calc}mg/mm^3	1.559
m/mm^{-1}	0.145
F(000)	560.0
Crystal size/mm^3	0.46 × 0.19 × 0.04
2Θ range for data collection	5.12 to 56.56°
Index ranges	-8 ≤ h ≤ 13, -11 ≤ k ≤ 16, -11 ≤ l ≤ 11
Reflections collected	4291
Independent reflections	2662[R_{int} = 0.0370]
Data/restraints/parameters	2662/1/176
Goodness-of-fit on F^2	1.018
Final R indexes [I>=2σ (I)]	R_1 = 0.0569, wR_2 = 0.1060
Final R indexes [all data]	R_1 = 0.1182, wR_2 = 0.1369
Largest diff. peak/hole / e $Å^{-3}$	0.22/-0.25
Flack parameter	-1.1(14)

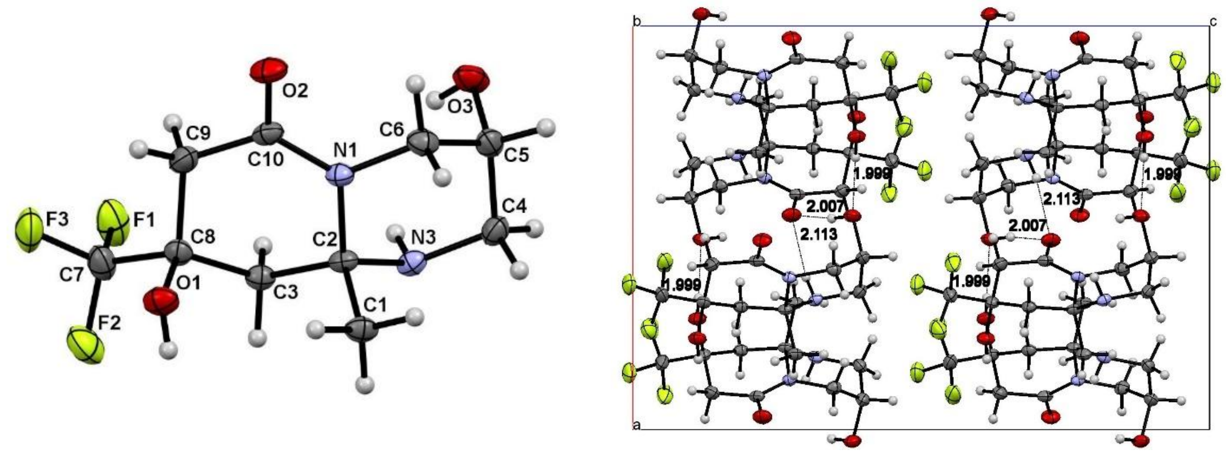

(**a**) (**b**)

Figure S3: ORTEP view of compound **4a**[tt] (**a**) and crystal packing (**b**) showing the thermal ellipsoids at 50% probability (CCDC 2479555).

**Table S3: Crystal data and structure refinement for 4a[tt]**	
Empirical formula	$C_{10}H_{15}F_3N_2O_3$
Formula weight	268.24
Temperature/K	295(2)
Crystal system	orthorhombic
Space group	Pbca
a/Å	12.5557(4)
b/Å	10.2140(5)
c/Å	17.9251(8)
α/°	90
β/°	90
γ/°	90
Volume/Å^3	2298.78(17)
Z	8
ρ_{calc}g/cm^3	1.550
μ/mm^{-1}	0.145
F(000)	1120.0
Crystal size/mm^3	0.47 × 0.18 × 0.07
Radiation	MoKα (λ = 0.71073)
2Θ range for data collection/°	4.544 to 61.116
Index ranges	-8 ≤ h ≤ 17, -8 ≤ k ≤ 14, -23 ≤ l ≤ 20
Reflections collected	6748
Independent reflections	3072 [R_{int} = 0.0332, R_{sigma} = 0.0518]
Data/restraints/parameters	3072/0/180
Goodness-of-fit on F^2	0.939
Final R indexes [I>=2σ (I)]	R_1 = 0.0498, wR_2 = 0.1239
Final R indexes [all data]	R_1 = 0.0990, wR_2 = 0.1580
Largest diff. peak/hole / e Å^{-3}	0.24/-0.18

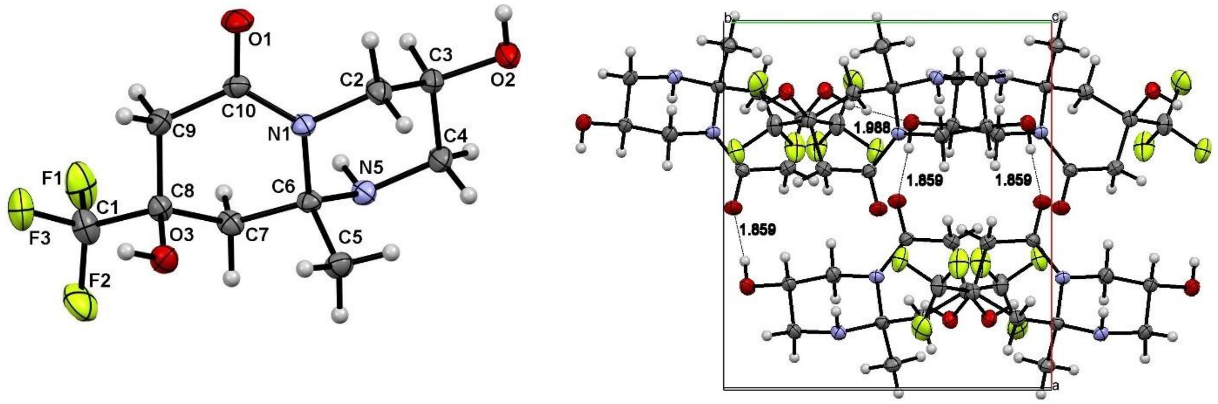

(**a**) (**b**)

Figure S4: ORTEP view of compound **4a^{tc}** (**a**) and crystal packing (**b**) showing the thermal ellipsoids at 50% probability (CCDC 2479556).

**Table S4: Crystal data and structure refinement for 4a^{tc}**	
Empirical formula	$C_{10}H_{15}F_3N_2O_3$
Formula weight	268.24
Temperature/K	295(2)
Crystal system	monoclinic
Space group	$P2_1/c$
a/Å	10.2698(4)
b/Å	9.1246(3)
c/Å	12.2297(5)
α/°	90
β/°	96.719(4)
γ/°	90
Volume/Å^3	1138.15(7)
Z	4
ρ_{calc}g/cm^3	1.565
μ/mm^{-1}	0.146
F(000)	560.0
Crystal size/mm^3	0.49 × 0.37 × 0.28
Radiation	MoKα (λ = 0.71073)
2Θ range for data collection/°	5.584 to 61.56
Index ranges	-13 ≤ h ≤ 13, -12 ≤ k ≤ 7, -17 ≤ l ≤ 10
Reflections collected	5278
Independent reflections	3090 [R_{int} = 0.0167, R_{sigma} = 0.0339]
Data/restraints/parameters	3090/0/180
Goodness-of-fit on F^2	1.012
Final R indexes [I>=2σ (I)]	R_1 = 0.0456, wR_2 = 0.1079
Final R indexes [all data]	R_1 = 0.0718, wR_2 = 0.1259
Largest diff. peak/hole / e Å^{-3}	0.22/-0.22

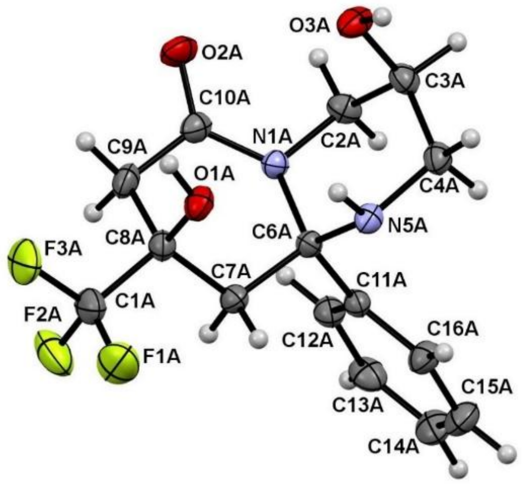

(**a**)

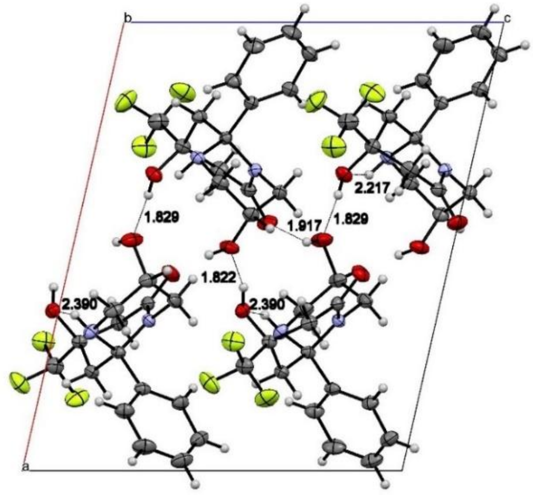

(**b**)

Figure S5: ORTEP view of compound **4d^ct^** (**a**) and crystal packing (**b**) showing the thermal ellipsoids at 50% probability (CCDC 2479557).

**Table S5: Crystal data and structure refinement for 4d^{ct}**	
Empirical formula	$C_{15}H_{17}F_3N_2O_3$
Formula weight	330.30
Temperature/K	295(2)
Crystal system	monoclinic
Space group	Pc
a/Å	14.5622(6)
b/Å	8.7117(3)
c/Å	12.0539(5)
α/°	90
β/°	102.816(4)
γ/°	90
Volume/Å^3	1491.08(10)
Z	4
ρ_{calc}g/cm^3	1.471
μ/mm^{-1}	0.127
F(000)	688.0
Crystal size/mm^3	0.47 × 0.26 × 0.15
Radiation	MoKα (λ = 0.71073)
2Θ range for data collection/°	4.676 to 59.122
Index ranges	-13 ≤ h ≤ 19, -12 ≤ k ≤ 5, -15 ≤ l ≤ 16
Reflections collected	6483
Independent reflections	4841 [R_{int} = 0.0261, R_{sigma} = 0.0555]
Data/restraints/parameters	4841/2/448
Goodness-of-fit on F^2	1.007
Final R indexes [I>=2σ (I)]	R_1 = 0.0449, wR_2 = 0.0788
Final R indexes [all data]	R_1 = 0.0811, wR_2 = 0.0967
Largest diff. peak/hole / e Å^{-3}	0.19/-0.19
Flack parameter	-0.8(7)

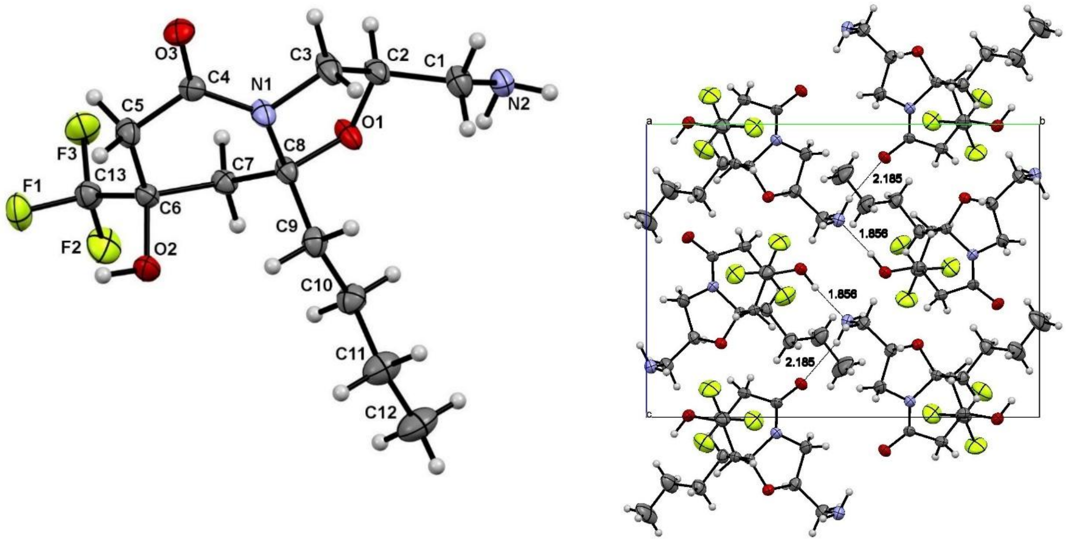

(**a**) (**b**)

Figure S6: ORTEP view of compound **5c**^{tc} (**a**) and crystal packing (**b**) showing the thermal ellipsoids at 50% probability (CCDC 2479558).

**Table S6: Crystal data and structure refinement for 5a^{tc}**	
Empirical formula	$C_{13}H_{21}F_3N_2O_3$
Formula weight	310.32
Temperature/K	295(2)
Crystal system	monoclinic
Space group	$P2_1/c$
a/Å	8.2915(3)
b/Å	16.8455(6)
c/Å	10.8081(4)
α/°	90
β/°	102.988(4)
γ/°	90
Volume/Å^3	1471.00(10)
Z	4
ρ_{calc}g/cm^3	1.401
μ/mm^{-1}	0.123
F(000)	656.0
Crystal size/mm^3	0.42 × 0.34 × 0.23
Radiation	MoKα (λ = 0.71073)
2Θ range for data collection/°	4.836 to 61.582
Index ranges	-6 ≤ h ≤ 11, -23 ≤ k ≤ 13, -14 ≤ l ≤ 10
Reflections collected	6513
Independent reflections	3917 [R_{int} = 0.0253, R_{sigma} = 0.0453]
Data/restraints/parameters	3917/0/207
Goodness-of-fit on F^2	1.031
Final R indexes [I>=2σ (I)]	R_1 = 0.0510, wR_2 = 0.1142
Final R indexes [all data]	R_1 = 0.0866, wR_2 = 0.1368
Largest diff. peak/hole / e Å^{-3}	0.22/-0.23

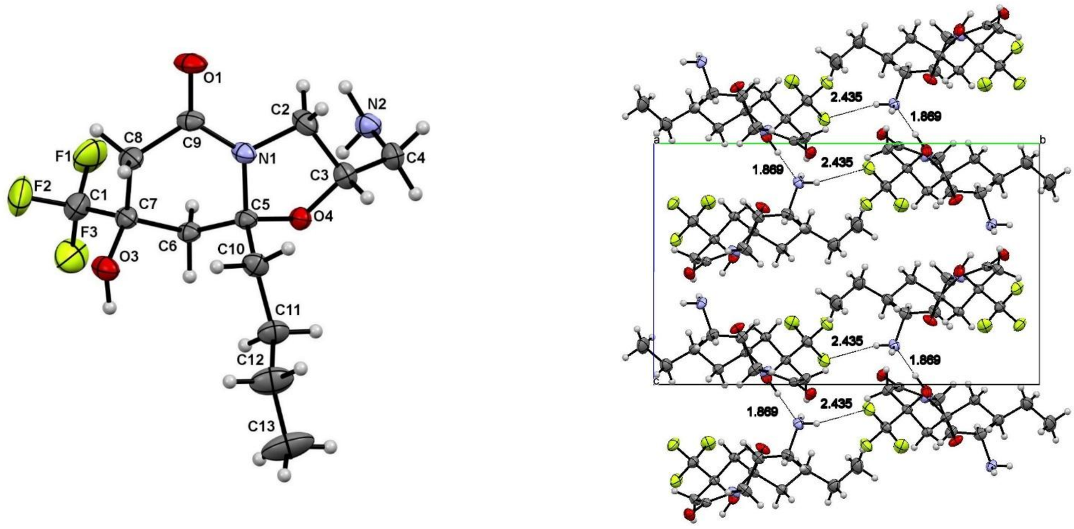

(**a**) (**b**)

Figure S7: ORTEP view of compound **5c^{tt}** (**a**) and crystal packing (**b**) showing the thermal ellipsoids at 50% probability (CCDC 2479559).

**Table S7: Crystal data and structure refinement for 5c^{tt}**	
Empirical formula	$C_{13}H_{21}F_3N_2O_3$
Formula weight	310.32
Temperature/K	295(2)
Crystal system	monoclinic
Space group	$P2_1/c$
a/Å	8.8586(4)
b/Å	15.4663(7)
c/Å	11.8821(5)
α/°	90
β/°	104.124(5)
γ/°	90
Volume/Å^3	1578.75(13)
Z	4
ρ_{calc}g/cm^3	1.306
μ/mm^{-1}	0.115
F(000)	656.0
Crystal size/mm^3	0.46 × 0.19 × 0.06
Radiation	MoKα (λ = 0.71073)
2Θ range for data collection/°	4.408 to 62.088
Index ranges	-11 ≤ h ≤ 12, -21 ≤ k ≤ 18, -9 ≤ l ≤ 16
Reflections collected	7070
Independent reflections	4287 [R_{int} = 0.0379, R_{sigma} = 0.0762]
Data/restraints/parameters	4287/0/208
Goodness-of-fit on F^2	1.005
Final R indexes [I>=2σ (I)]	R_1 = 0.0608, wR_2 = 0.1332
Final R indexes [all data]	R_1 = 0.1292, wR_2 = 0.1711
Largest diff. peak/hole / e Å^{-3}	0.24/-0.17

Table S8: Hydrogen bonds with H..A < r(A) + 2.000 Å and <DHA > 110°					
D–H	d(D–H)	d(H..A)	<DHA	d(D..A)	A
**4a**[cc]					
O1–H1	0.85(2)	1.95(2)	169(1)	2.788(2)	N3 [x, 1/2-y, z+1/2]
O2–H2	0.80(2)	1.98(2)	164(1)	2.762(2)	O3 [1-x, 1-y, 1-z]
N3–H3	0.865(15)	2.19(2)	139(1)	2.895(15)	O1
**4a**[ct]					
O1–H1	0.86(4)	1.89(4)	173(3)	2.739(5)	O2 [-x-3/2, y, z+1/2]
O3–H3	0.93(6)	1.94(6)	173(4)	2.863(5)	N5 [x-1/2, -y-1, z]
N5–H5	0.77(4)	2.42(4)	129(4)	2.974(5)	O1
**4a**[tt]					
O1–H1	0.79(3)	2.00(3)	170(2)	2.782(2)	O3 [x+1/2, y, 3/2-z]
O3–H3	0.88(3)	2.00(3)	168(2)	2.868(2)	O2 [1-x, y-1/2, 3/2-z]
N3–H3A	0.89(2)	2.11(2)	165(1)	2.983(2)	O2 [1-x, y-1/2, +3/2-z]
**4a**[tc]					
O2–H2	0.84(2)	1.86(2)	173(2)	2.693(2)	O1 [1-x, y-1/2, 1/2-z]
O3–H3A	0.84(2)	1.99(2)	166(2)	2.809(2)	O2 [x, y+1, z]
**4d**[ct]					
N5–H5	0.83(5)	2.39(5)	131(3)	2.998(5)	O1
O3–H3A	0.79(5)	1.91(5)	157(4)	2.651(5)	O2A
O1–H1	0.95(5)	1.83(5)	164(3)	2.755(5)	O3A
N5A–H5A	0.91(4)	2.22(4)	132(2)	2.912(4)	O1A
O3A–H3AB	0.83(5)	1.88(5)	169(3)	2.691(5)	O2 [x, -y, z-1/2]
O1A–H1A	0.80(4)	1.84(4)	176(3)	2.638(4)	O3 [x, 1-y, z-1/2]
**5c**[tc]					
O3–H3	0.93(3)	1.86(3)	174(2)	2.782(3)	N2 [1-x, y-1/2, 1/2-z]
N2–H2A	0.97(3)	2.18(3)	151(2)	3.064(3)	O1 [1-x, 2-y, 1-z]
N2–H2B	0.90(3)	2.59(3)	149(2)	3.392(3)	O3 [x, 3/2-y, z-1/2]
**5c**[tt]					
N2–H2A	0.88(2)	2.43(2)	138(2)	3.145(2)	F1 [1-x, y+1/2, 1/2-z]
N2–H2B	0.92(2)	2.31(2)	172(2)	3.227(2)	O3 [x, 1/2-y, z-1/2]
O2–H2C	0.93(3)	1.87(3)	174(2)	2.796(2)	N2 [x+1, 1/2-y, z+1/2]

**Table S9:** Dihedral angles of heterocycles in compounds **4a**,**d**.			
**Compound**	Pyridone ring	Pyrimidine ring	angles
**4a**[tt]	C2 C3 C8 C9 C10 N1	C4 C5 C6 N1 C2 N3	24.8(2)
**4a**[tc]	C6 C7 C8 C9 C10 N1	C2 C3 C4 N5 C6 N1	27.3(3)
**4a**[cc]	C2 C7 C8 C9 C10 N1	C4 C5 C6 N1 C2 N3	51.5(1)
**4a**[ct]	C6 C7 C8 C9 C10 N1	C2 C3 C4 N5 C6 N1	49.5(6)
**4d**[ct]	C6 C7 C8 C9 C10 N1<br>C6A C7A C8A C9A C10A N1A	C2 C3 C4 N5 C6 N1<br>C2A C3A C4A N5A C6A N1A	47.7(7)<br>53.9(5)

Cytotoxicity and anti-viral activity evaluation

Virus and cells. We used MDCK cells (ATCC CCL-34) from the collection of cell lines of the Saint Petersburg Pasteur Institute. Cells were cultured in 96-well culture plates in MEM medium with 10% fetal bovine serum («HyClone», USA), 40 U/ml gentamicin sulfate and 2.5 U/ml amphotericin B. Cell suspension with a concentration of 105 cells/ml was placed in the wells of the plates in volume of 100 μl and cultured until a complete monolayer formation for 24 h at 36 °C in the presence of 5% CO_2. The same medium without serum was used as a support medium for culturing cells with viruses.

We used influenza virus A/Puerto Rico/8/34 (H1N1) from the collection of the Saint Petersburg Pasteur Institute. The infectious titer of virus was determined by titration in 96-well plates with monolayers of MDCK cells. The results were evaluated visually according to the presence of the virus cytopathic action, the virus titer was calculated by the Spearman–Kerber method and represented in decimal logarithms of 50% tissue cytopathic doses in ml (lg TCD_{50}/ml).

Evaluation of cytotoxic properties of compounds. The assessment of toxicity of compounds was carried out based on evaluation of the cell viability using the reduction reaction of the tetrazolium dye MTT (3-(4,5-dimethyl-2-thiazolyl)-2,5-diphenyl-2*H*-tetrazolium bromide) by cells in culture. Its intensity shows the degree of cell viability as a result of dye reduction by mitochondrial and partially cytoplasmic dehydrogenases.

The test compounds in the concentration range of 3.7–300 μg/ml dissolved in the medium for cell cultivation were added to the plate wells in a volume of 200 μl and incubated for 48 h at 36 °C in atmosphere of 5% CO_2. At the end of the incubation period, the cells were washed with MEM medium, and 100 μl of a solution (0.5 mg/ml) of MTT in the cell medium was added to the plate wells. The cells were incubated at 36 °C at 5% CO_2 for 2 h and washed for 5 min with saline. The precipitate was dissolved in 100 μl of DMSO per well, and the optical density was measured using a Multiscan FC plate analyzer (Thermo Scientific) at a wavelength of 540 nm. Based on the obtained data, the 50% cytotoxic concentration (CC_{50}) was calculated, i.e., the concentration of the compound, which reduces the optical density in the wells by half compared to control cells without drugs. For this purpose, GraphPad Prism 6.01 software was used.

Evaluation of anti-viral activity of compounds. The compounds in appropriate concentrations were added to MDCK cells (0.1 ml per well). After 1 h of incubation, cells were infected with influenza virus A/Puerto Rico/8/34 (H1N1) (moi 0.01) and incubated

for 48 h at 36 °C and 5% CO_2. After that, cell viability was assessed by MTT test as described above. The cytoprotective activity of compounds was considered as their ability to increase the values of OD comparing to control wells (with virus only, no drugs). Based on the results obtained, the values of IC_{50}, i.e., concentration of compounds that result in 50% cells protection were calculated using GraphPad Prism 6.01 software. Values of IC_{50} obtained in μg/ml were then calculated into micromoles (μM). For each compound the value of selectivity index (SI) was calculated as ratio of CC_{50} to IC_{50}. Compounds with SI of 10 and higher were considered active. Ribavirin was used as a reference compound.

References

[1] Dolomanov, O. V.; Bourhis, L. J.; Gildea, R. J.; Howard, J. A. K.; Puschmann, H. *J. Appl. Crystallogr.* **2009**, *42*, 339–341. doi:10.1107/S0021889808042726

[2] Sheldrick, G. M. *Acta Crystallogr. C: Struct. Chem*. **2015**, *C71*, 3–8. doi:10.1107/S2053229614024218

[3] Sheldrick, G. M. *Acta Crystallogr. A: Found. Adv.* **2015**, *A71*, 3–8. doi:10.1107/S2053273314026370

Copies of ^{1}H, ^{19}F and ^{13}C NMR spectra

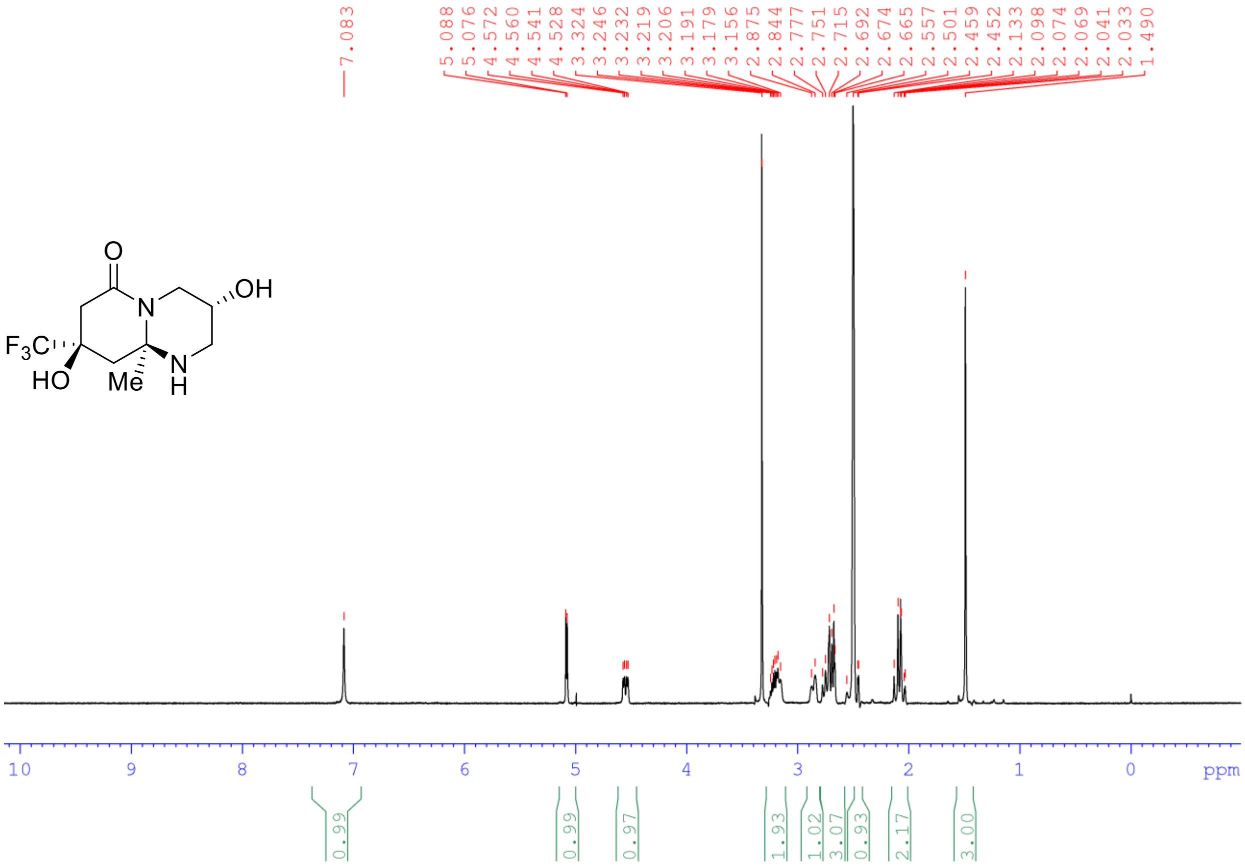

Figure S8: ^{1}H NMR (400 MHz, DMSO-d_6) spectrum of **4a**^{cc}.

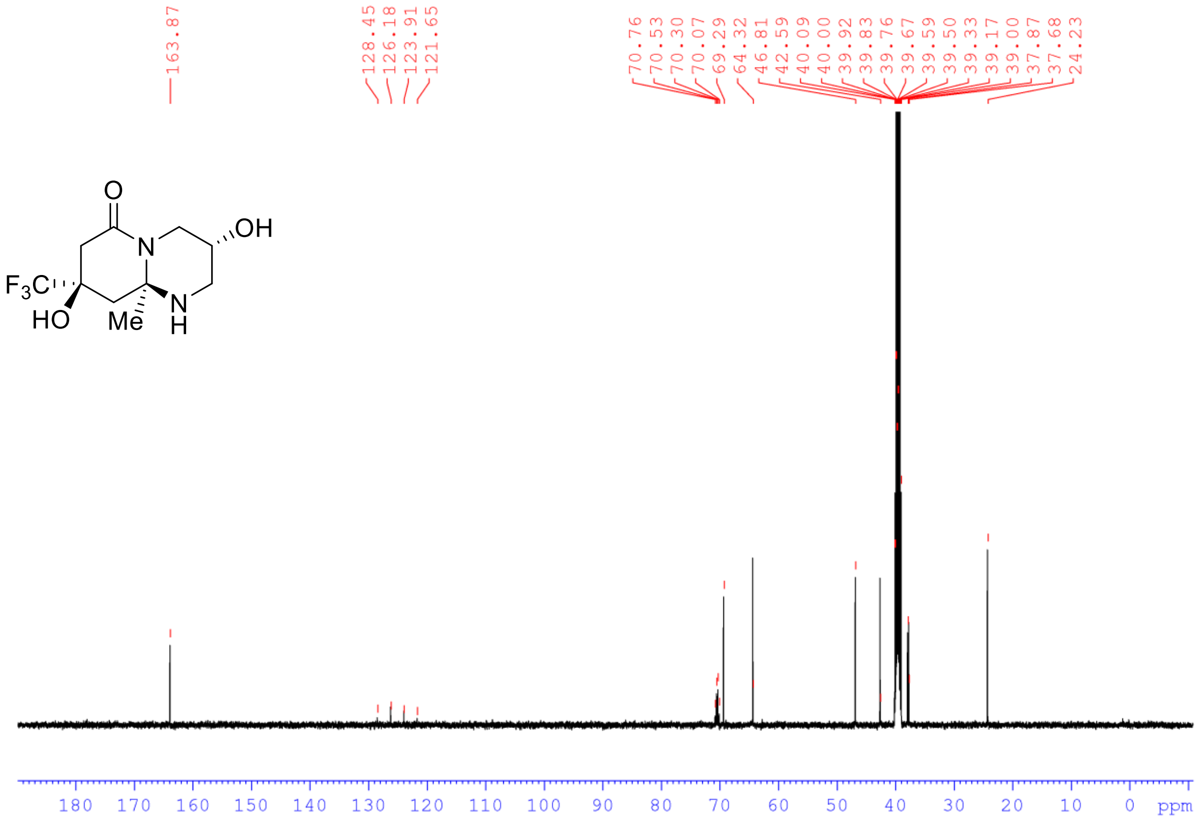

Figure S9: ^{13}C NMR (126 MHz, DMSO-d_6) spectrum of **4a**^{cc}.

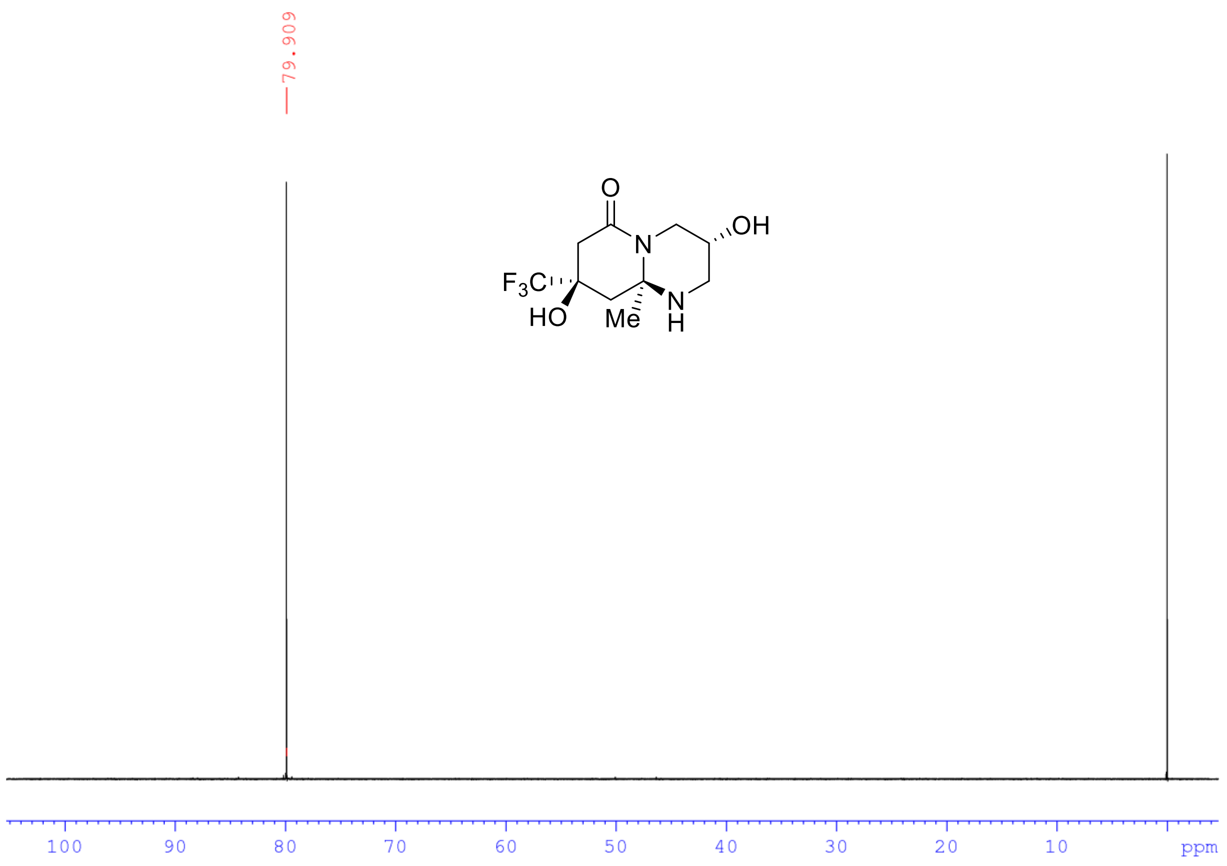

Figure S10: ^{19}F NMR (376 MHz, DMSO-d_6) spectrum of **4a^{cc}**.

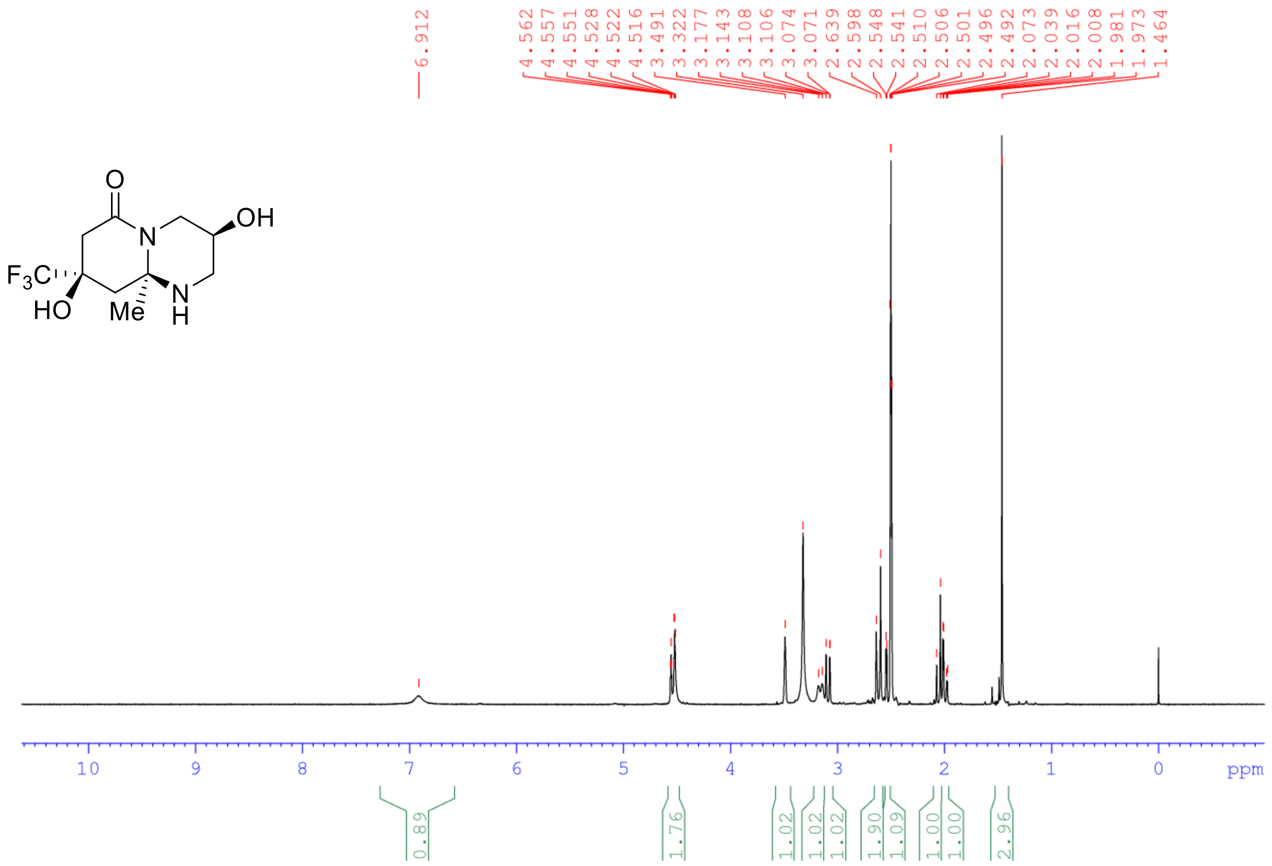

Figure S11: ^{1}H NMR (400 MHz, DMSO-d_6) spectrum of **4a^{ct}**.

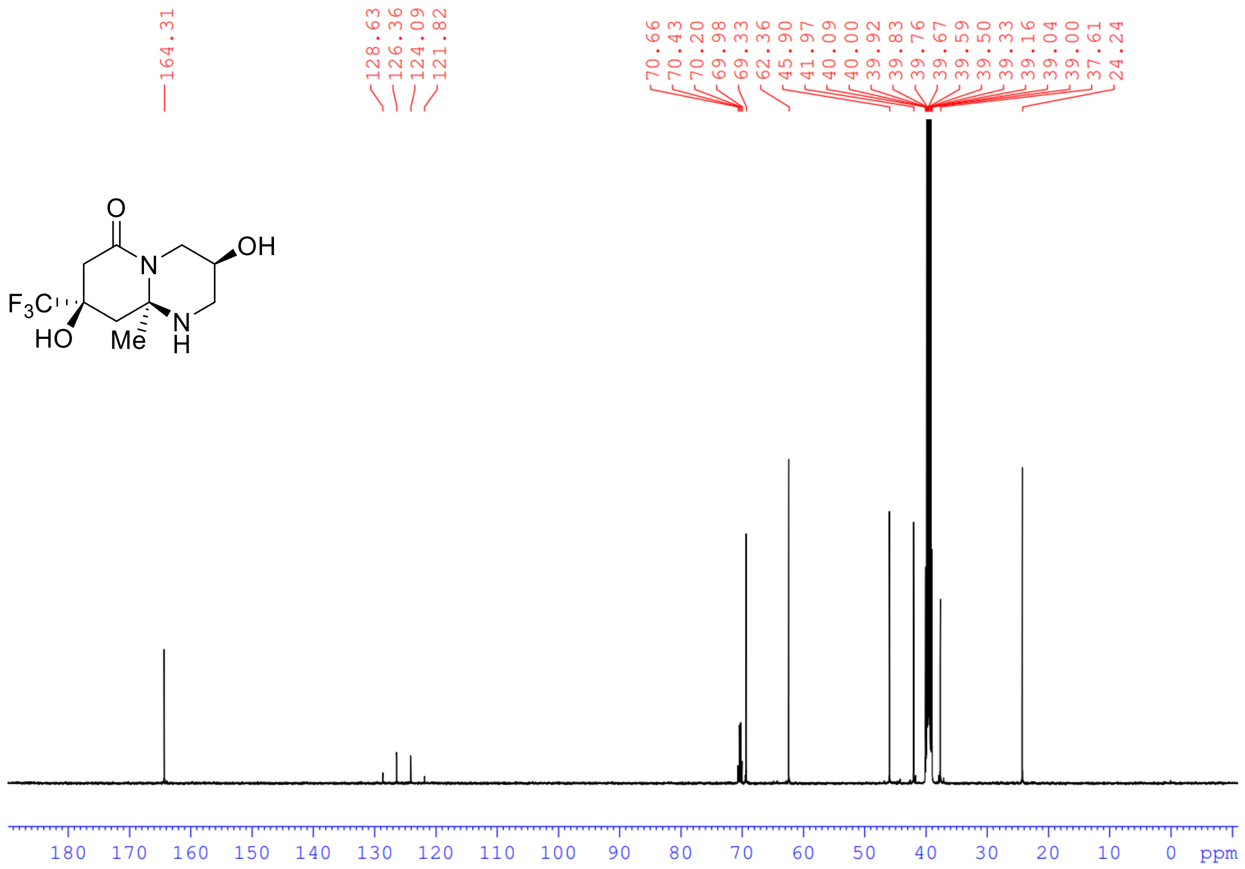

Figure S12: ^{13}C NMR (126 MHz, DMSO-d_6) spectrum of **4a**^{ct}.

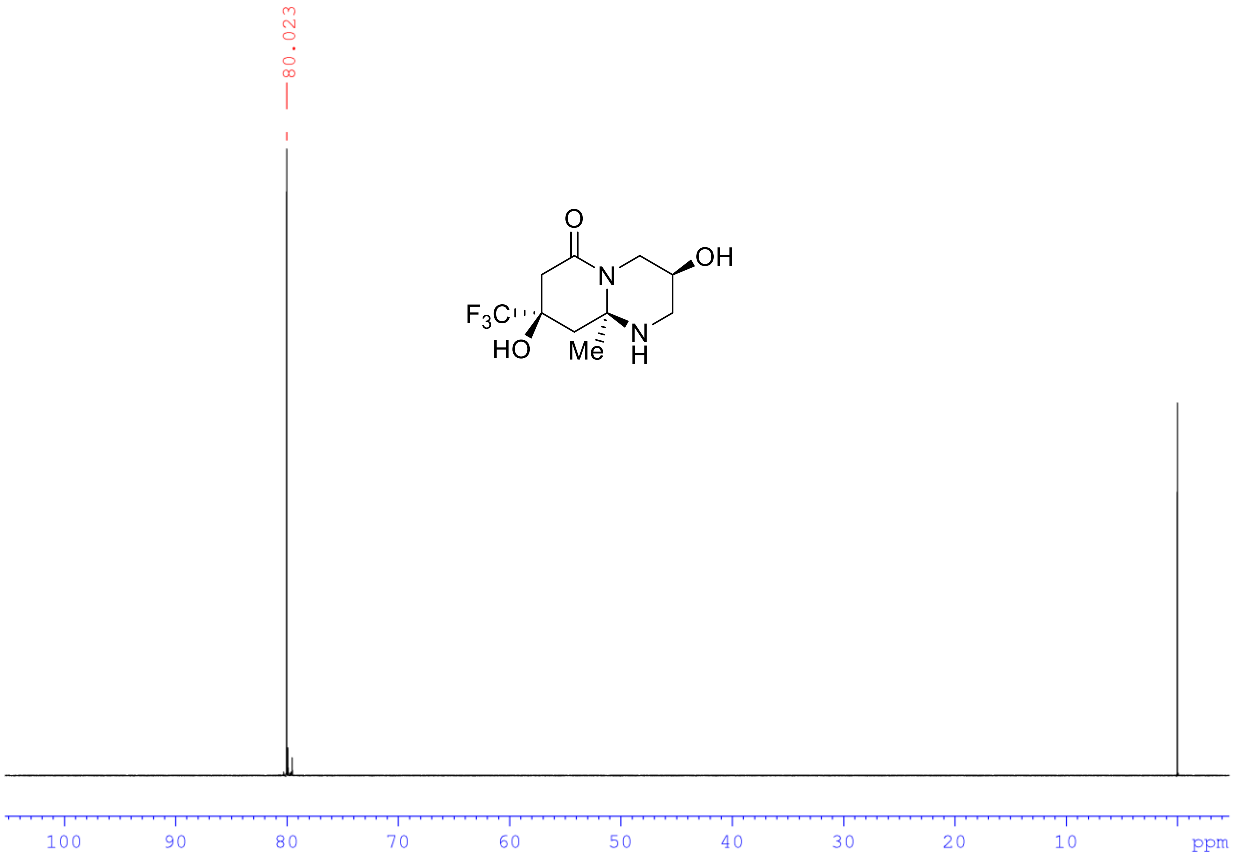

Figure S13: ^{19}F NMR (376 MHz, DMSO-d_6) spectrum of **4a**^{ct}.

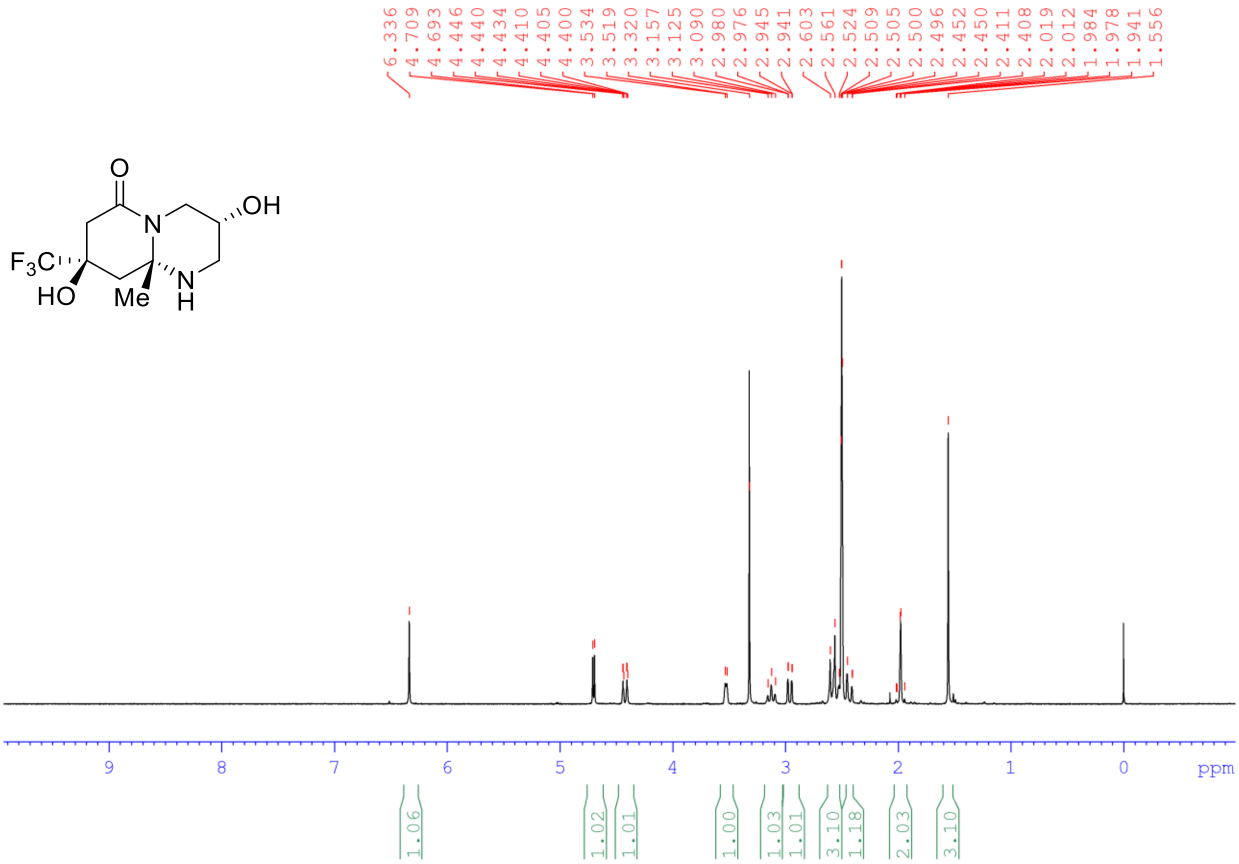

Figure S14: ^{1}H NMR (400 MHz, DMSO-d_6) spectrum of **4a^tt^**.

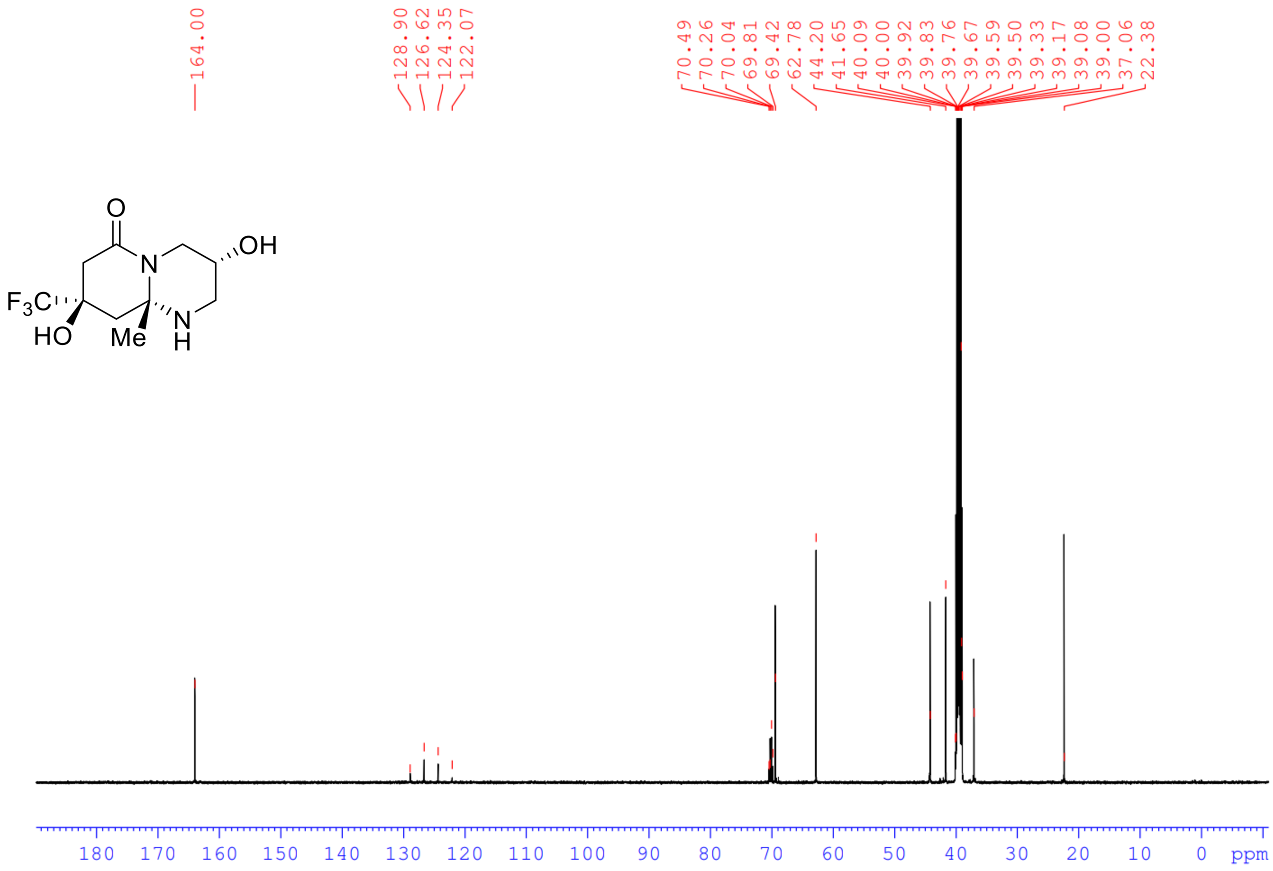

Figure S15: ^{13}C NMR (126 MHz, DMSO-d_6) spectrum of **4a^tt^**.

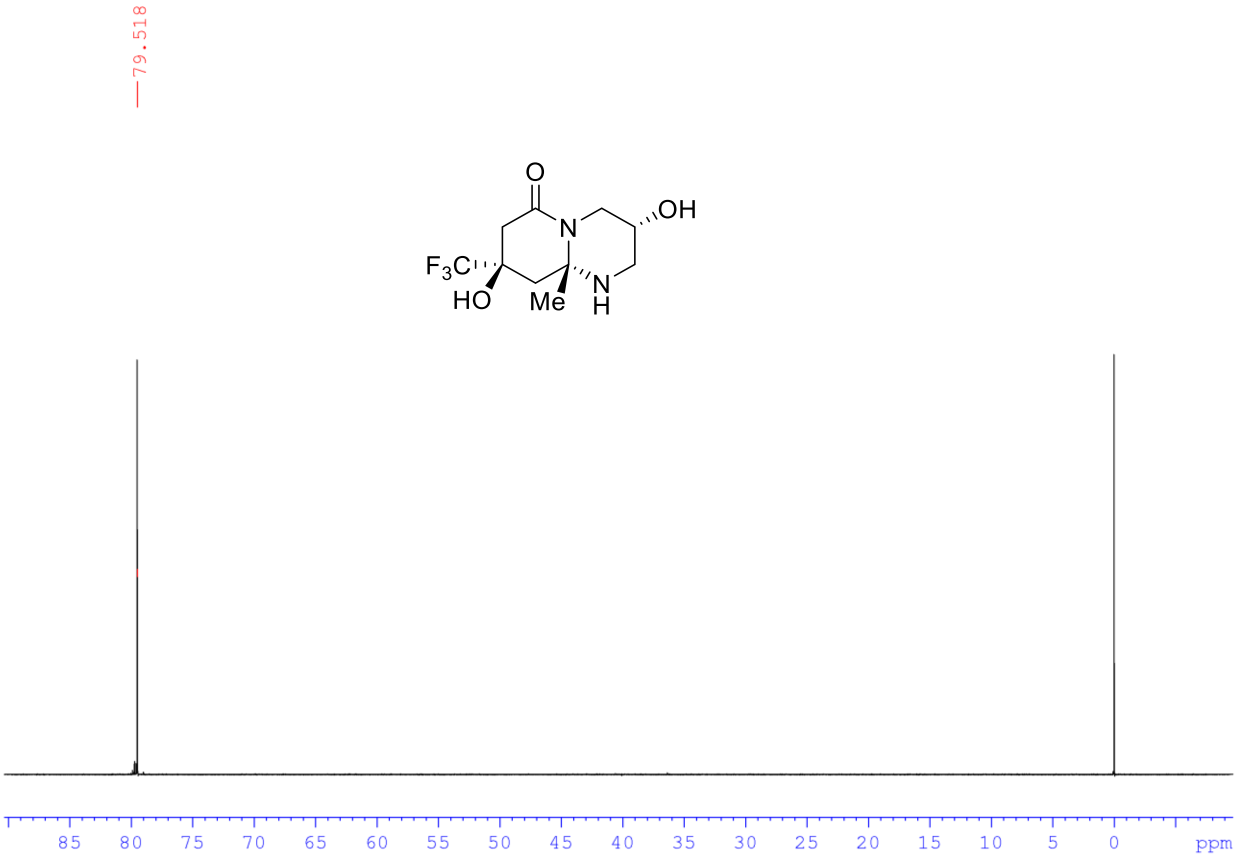

Figure S16: ^{19}F NMR (376 MHz, DMSO-d_6) spectrum of **4a^{tt}**.

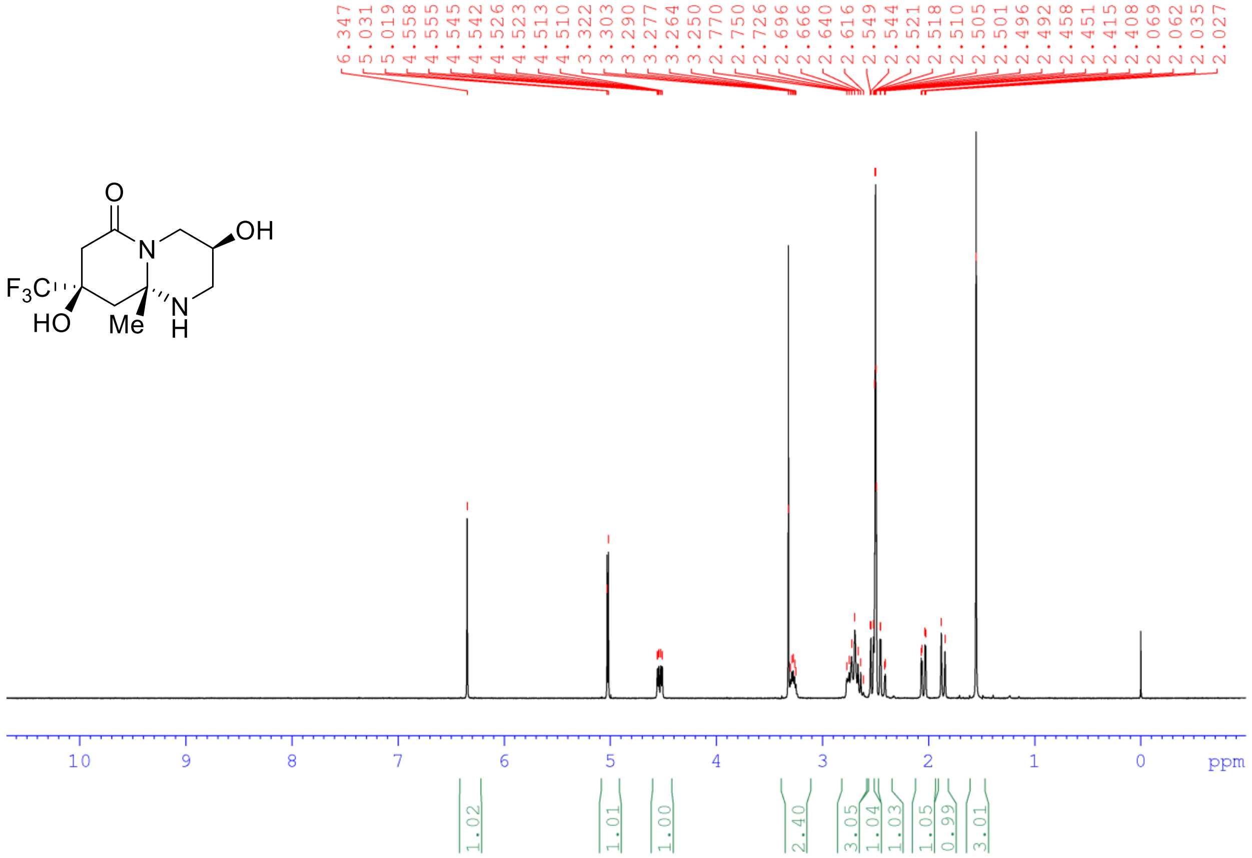

Figure S17: ^{1}H NMR (400 MHz, DMSO-d_6) spectrum of **4a^{tc}**.

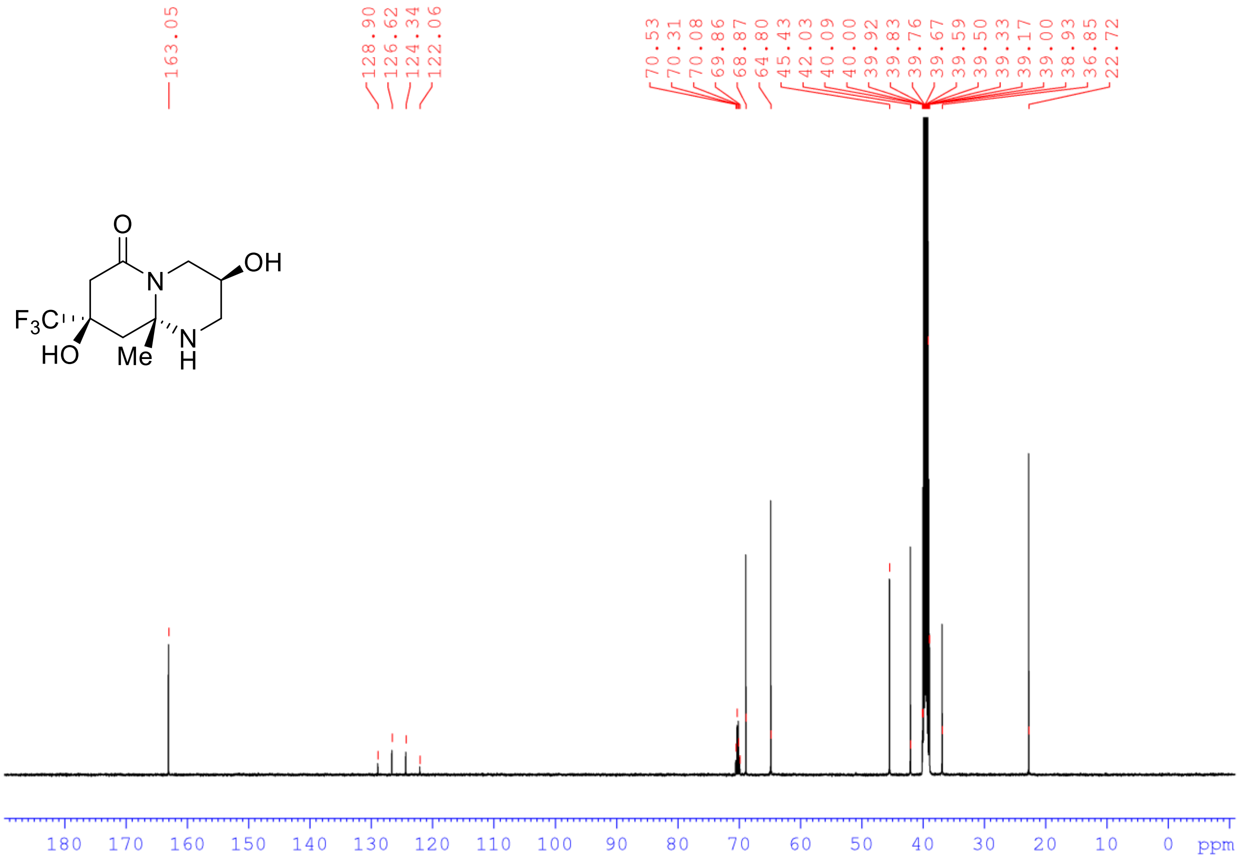

Figure S18: ^{13}C NMR (126 MHz, DMSO-d_6) spectrum of **4a**^{tc}.

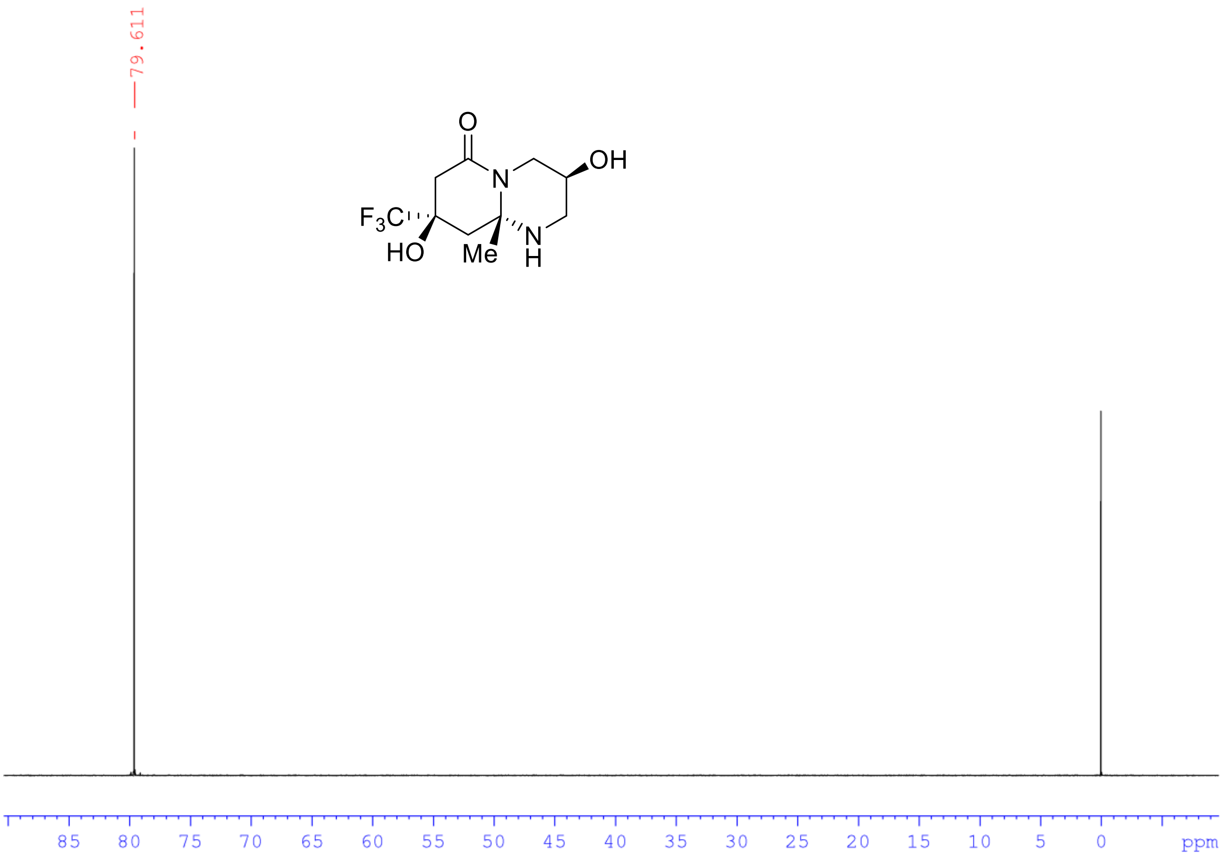

Figure S19: ^{19}F NMR (376 MHz, DMSO-d_6) spectrum of **4a**^{tc}.

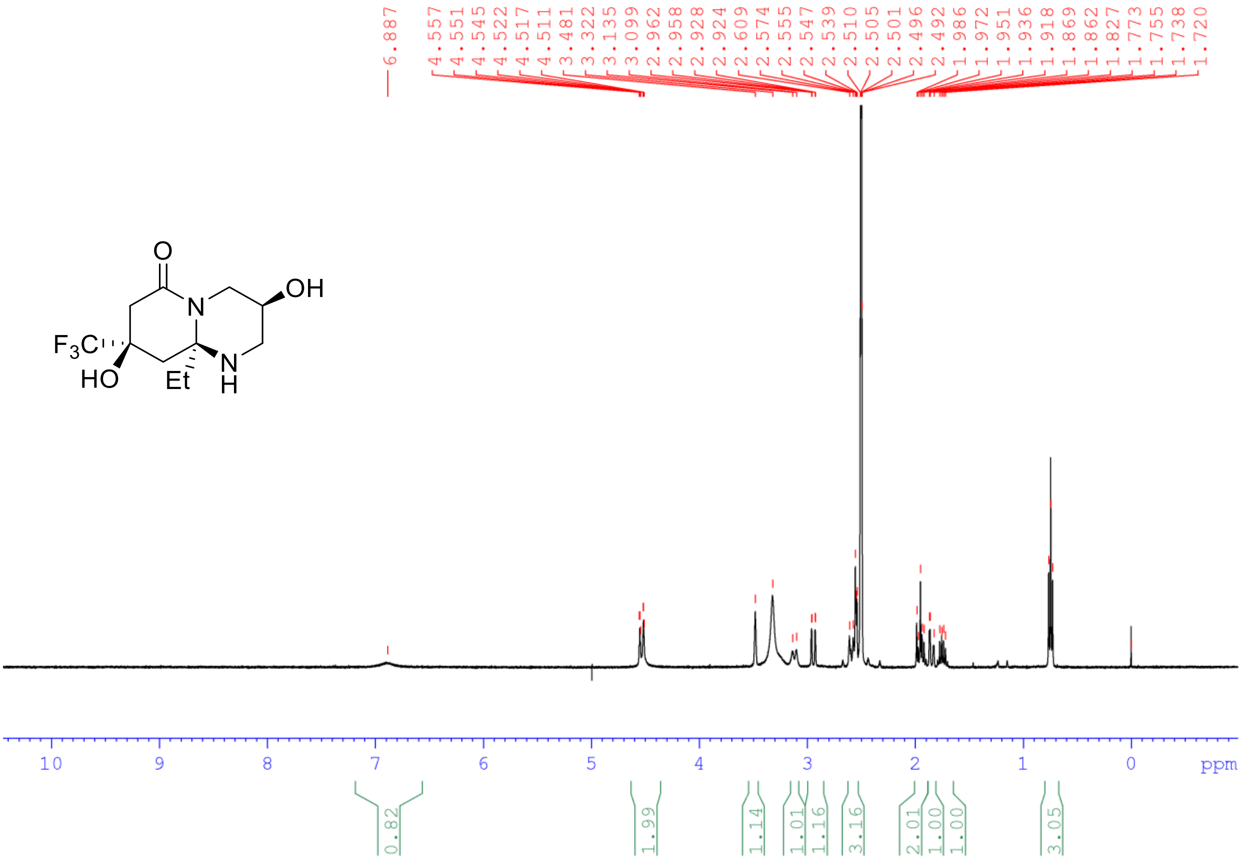

Figure S20: ^{1}H NMR (400 MHz, DMSO-*d*$_6$) spectrum of **4b**^{ct}.

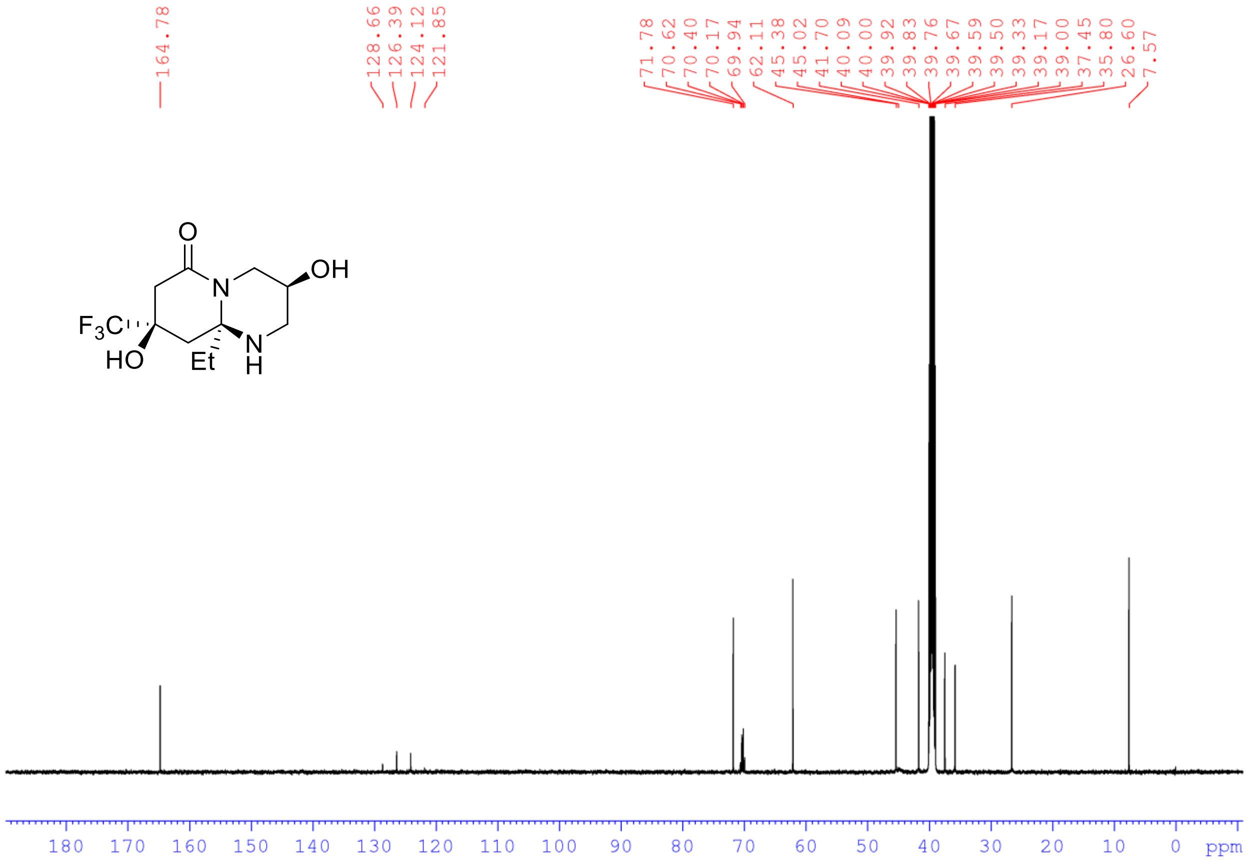

Figure S21: ^{13}C NMR (126 MHz, DMSO-*d*$_6$) spectrum of **4b**^{ct}.

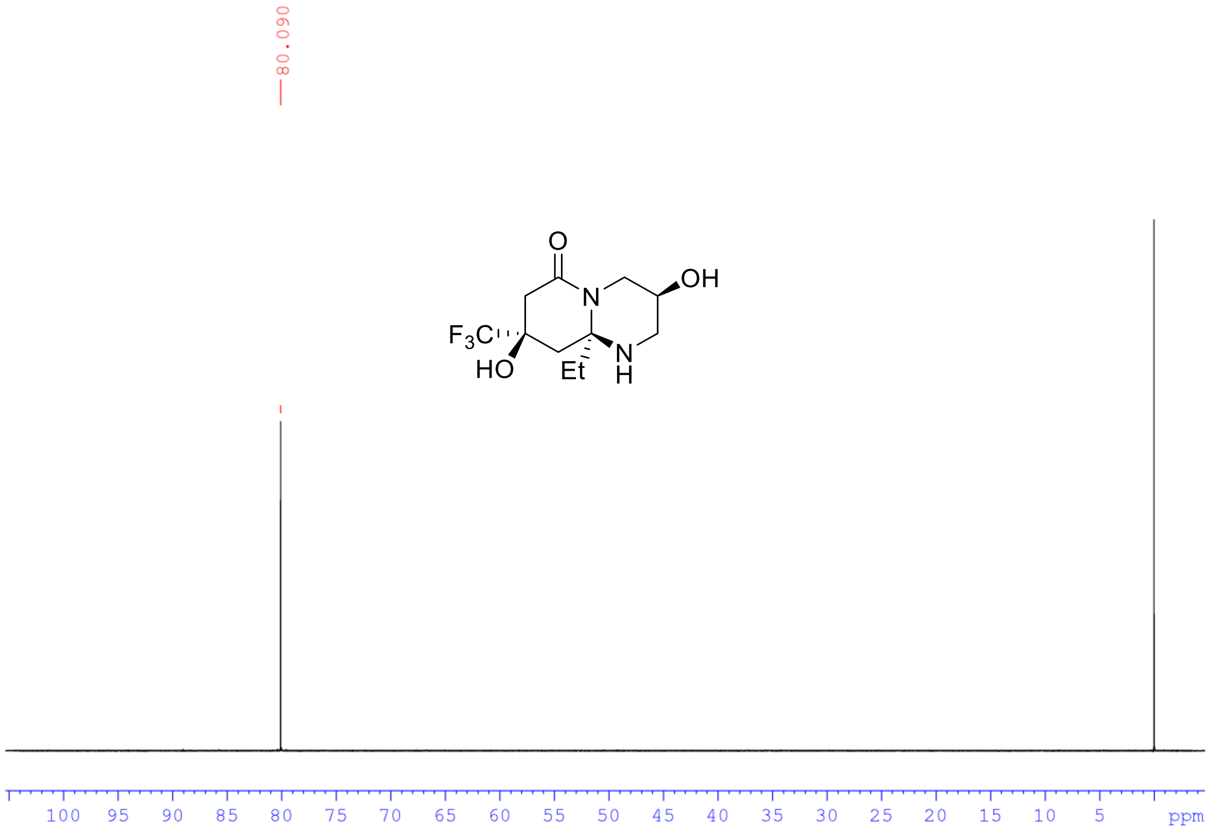

Figure S22: ^{19}F NMR (376 MHz, DMSO-d_6) spectrum of **4b^{ct}**.

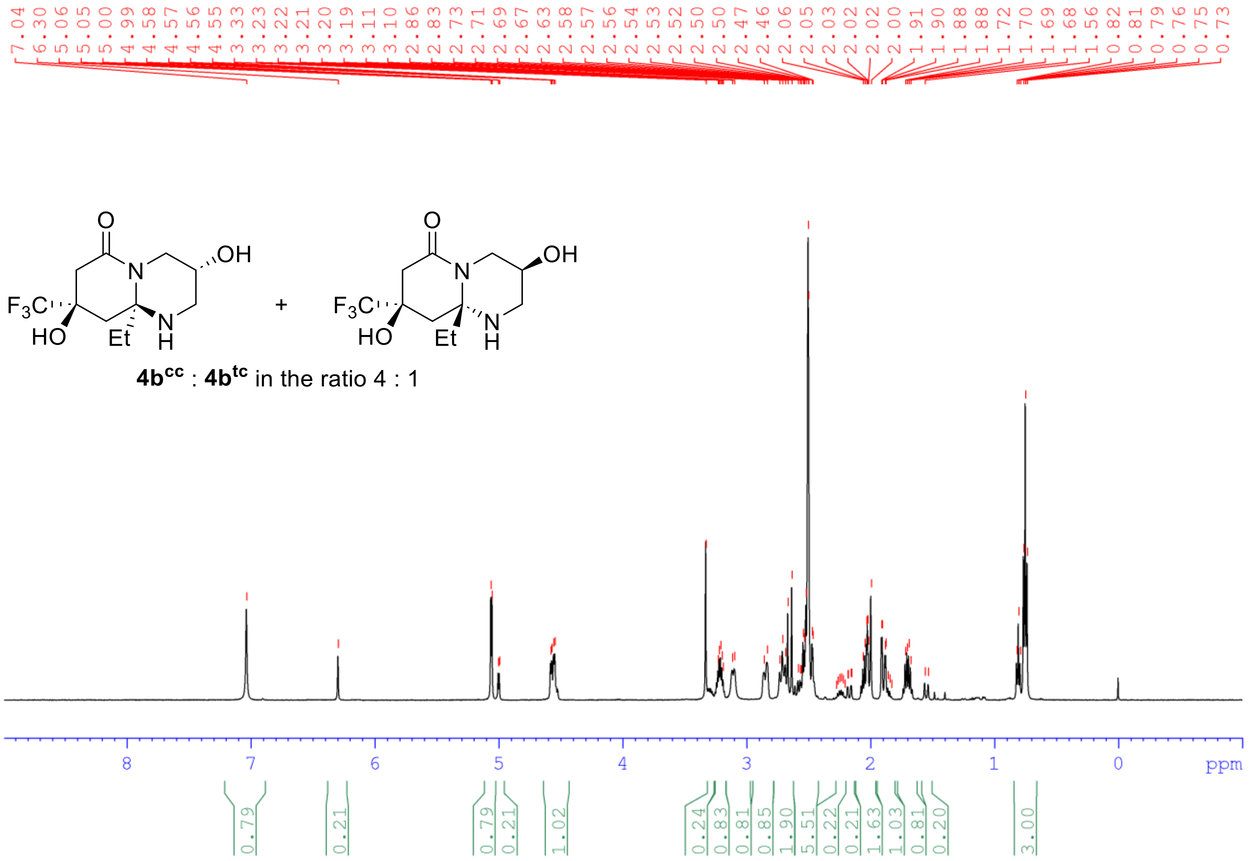

Figure S23: ^{1}H NMR (500 MHz, DMSO-d_6) spectrum of a mixture of diastereomers **4b^{cc}** and **4b^{tc}**.

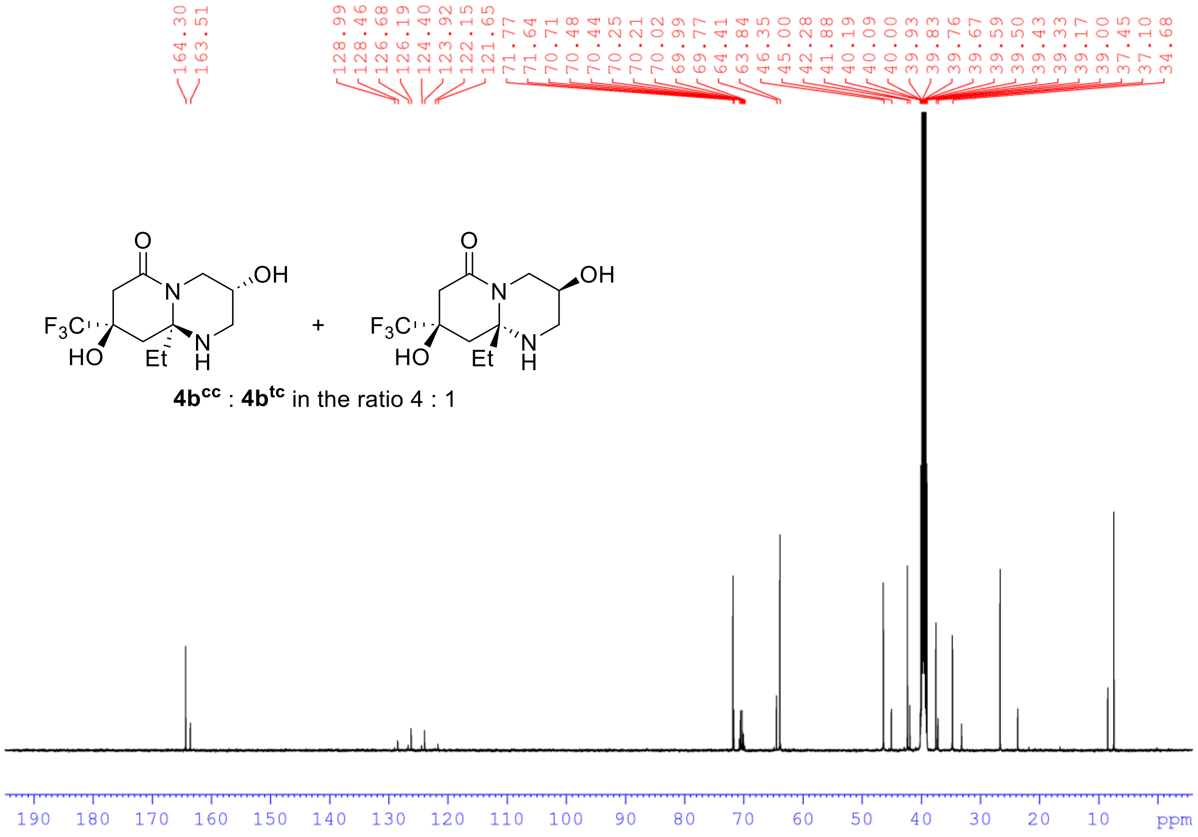

Figure S24: ^{13}C NMR (126 MHz, DMSO-d_6) spectrum of a mixture of diastereomers **4b**^{cc} and **4b**^{tc}.

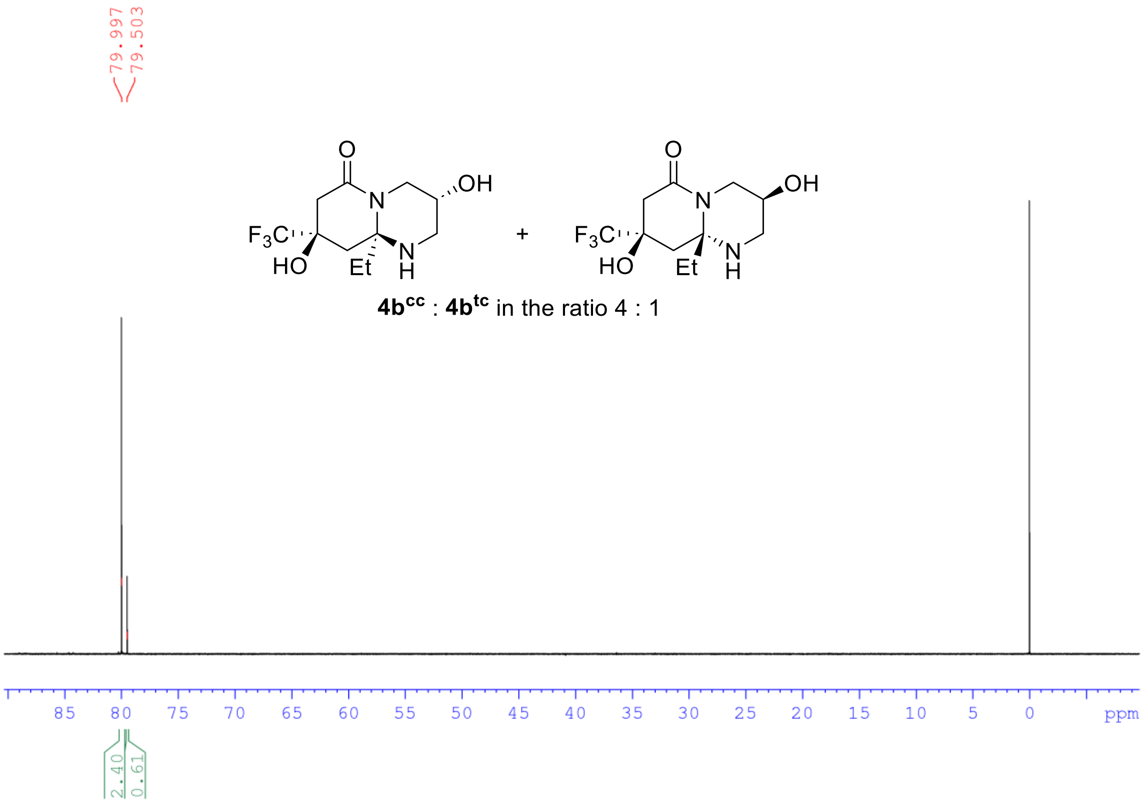

Figure S25: ^{19}F NMR (376 MHz, DMSO-d_6) spectrum of a mixture of diastereomers **4b**^{cc} and **4b**^{tc}.

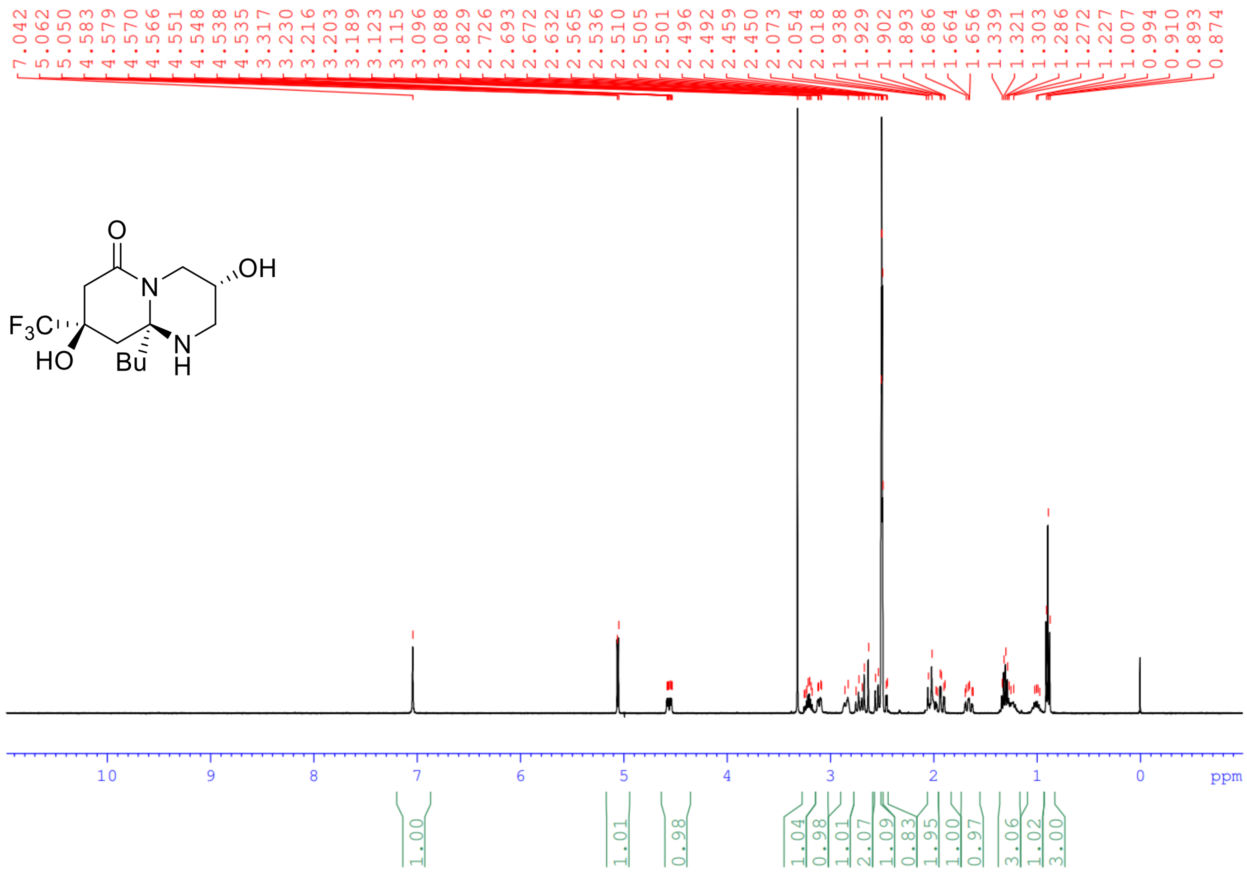

Figure S26: ^{1}H NMR (400 MHz, DMSO-d_6) spectrum of **4c**[cc].

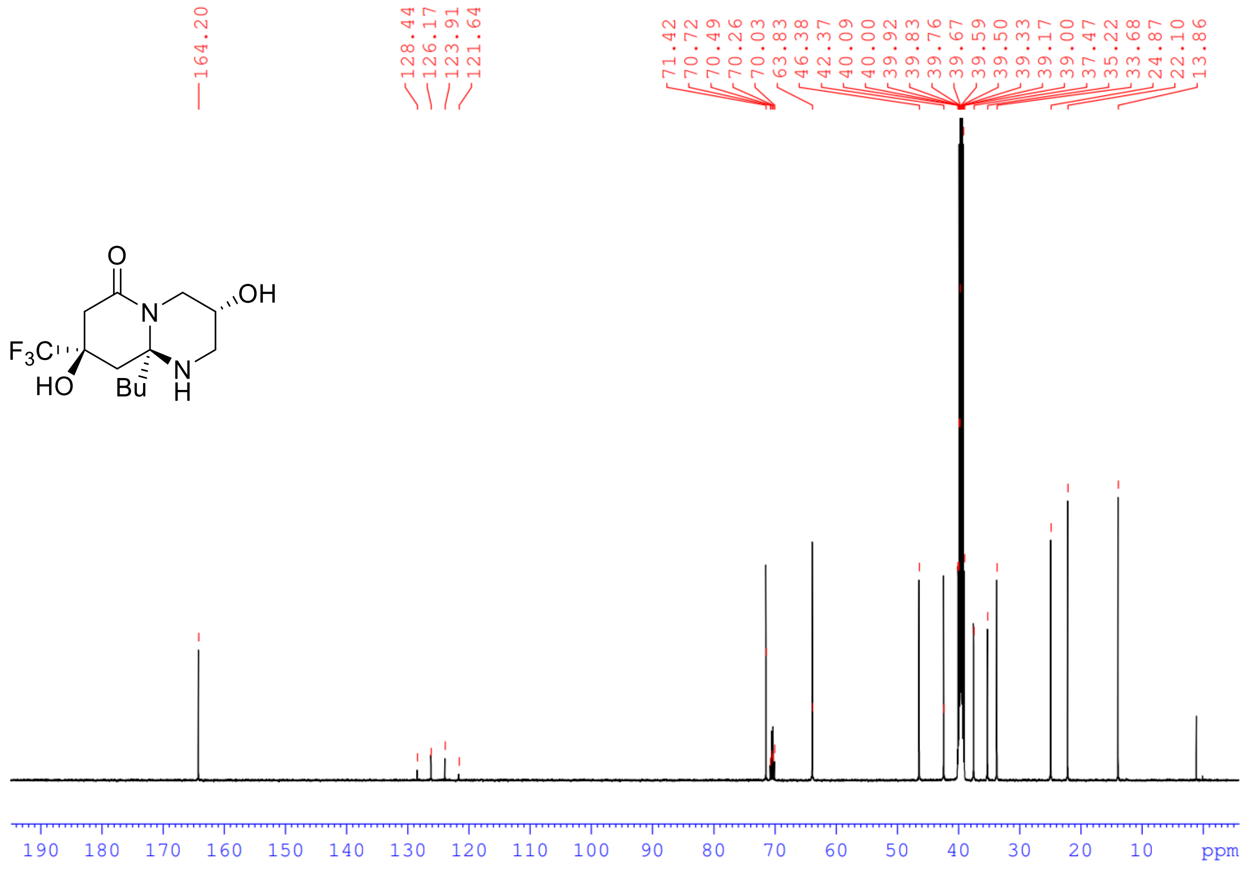

Figure S27: ^{13}C NMR (126 MHz, DMSO-d_6) spectrum of **4c**[cc].

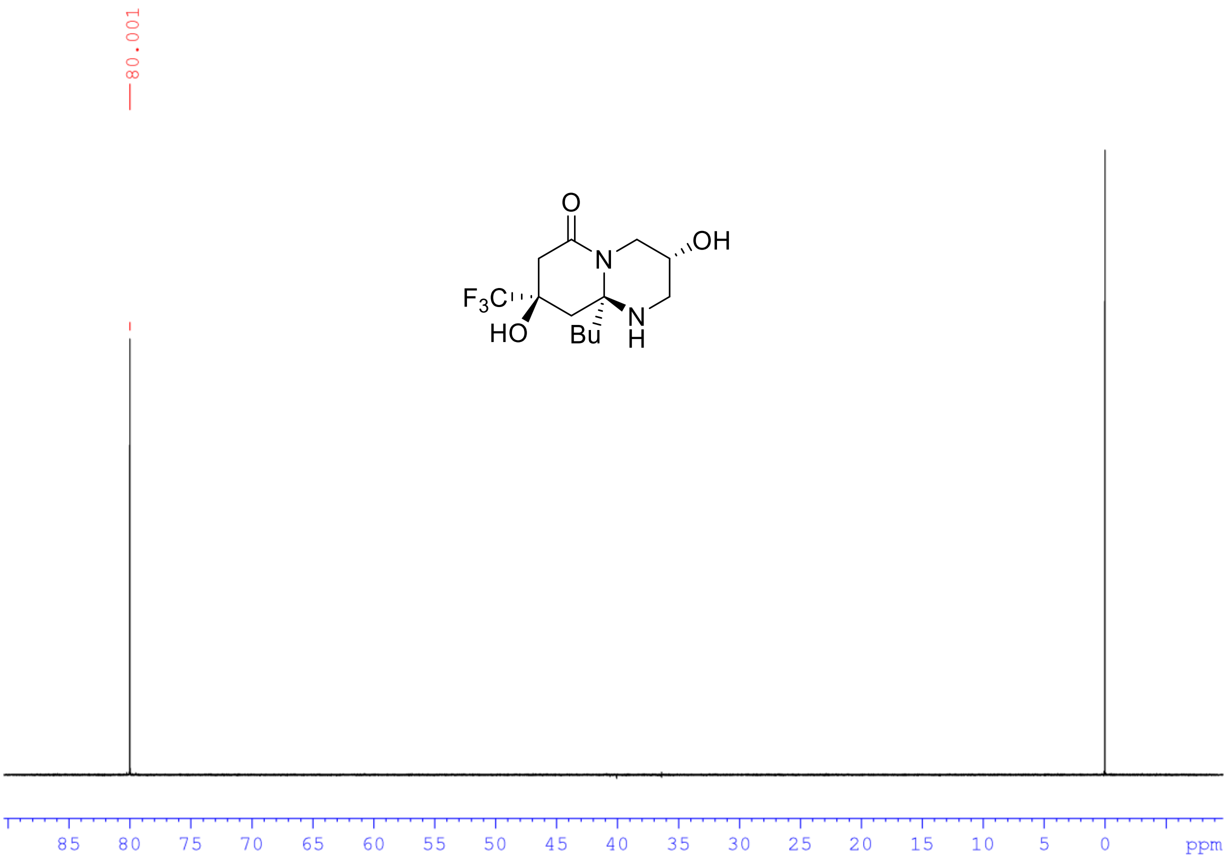

Figure S28: ^{19}F NMR (376 MHz, DMSO-d_6) spectrum of **4c**^{cc}.

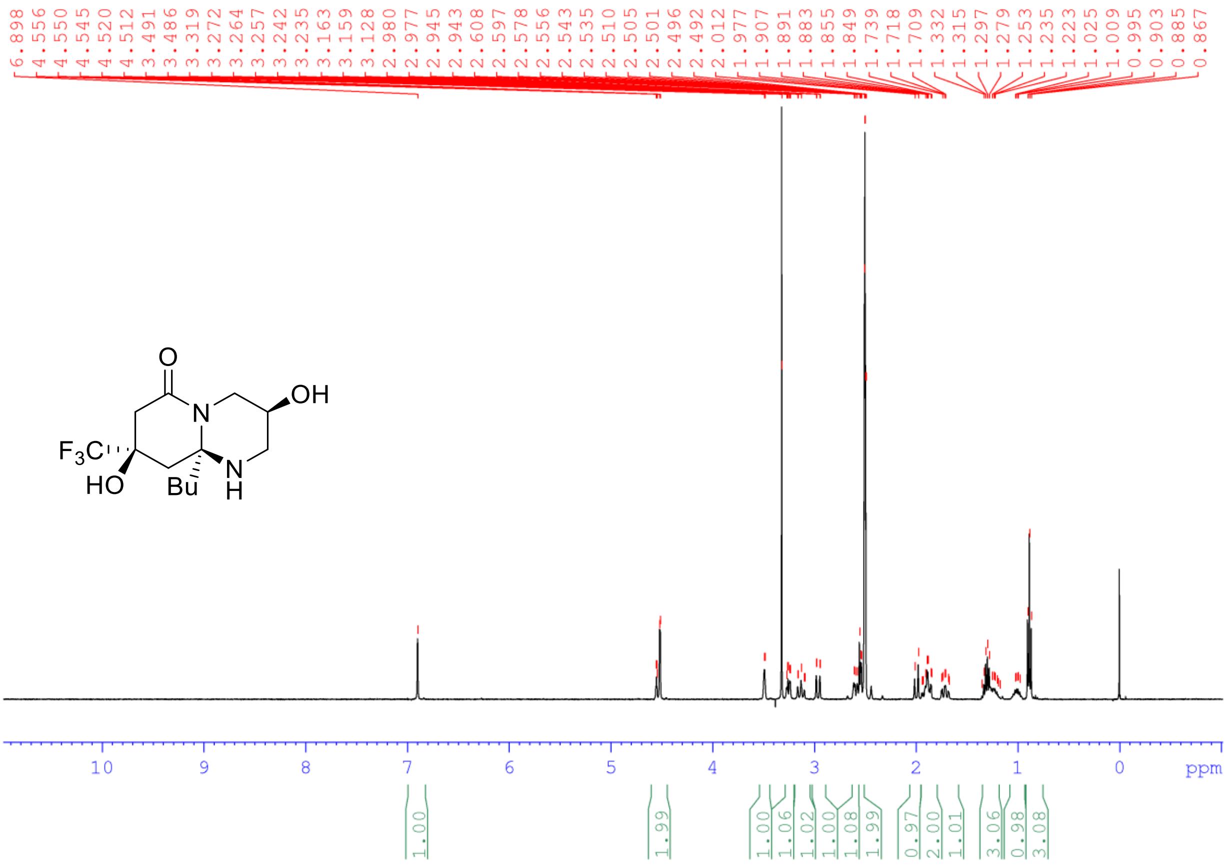

Figure S29: ^{1}H NMR (400 MHz, DMSO-d_6) spectrum of **4c**^{ct}.

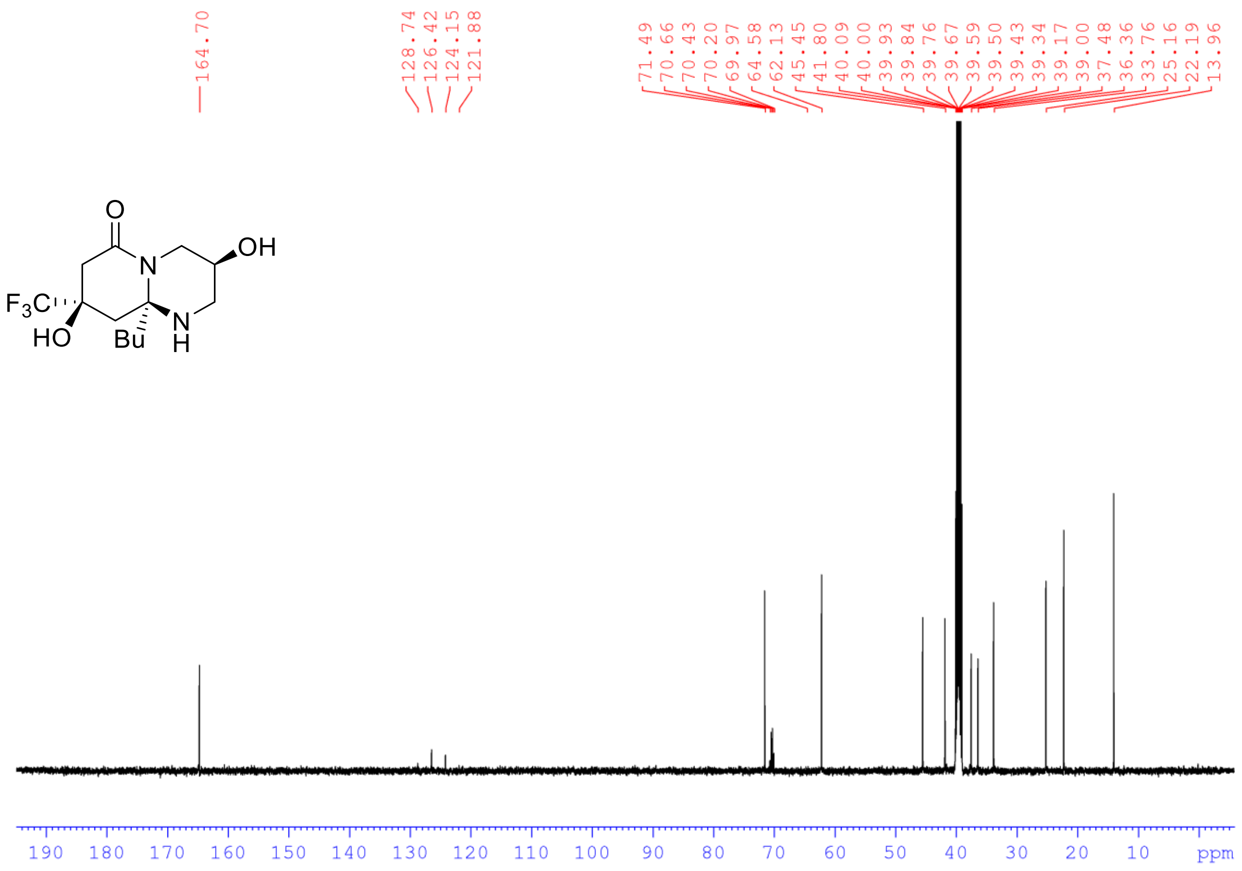

Figure S30: ^{13}C NMR (126 MHz, DMSO-d_6) spectrum of **4c**^{ct}.

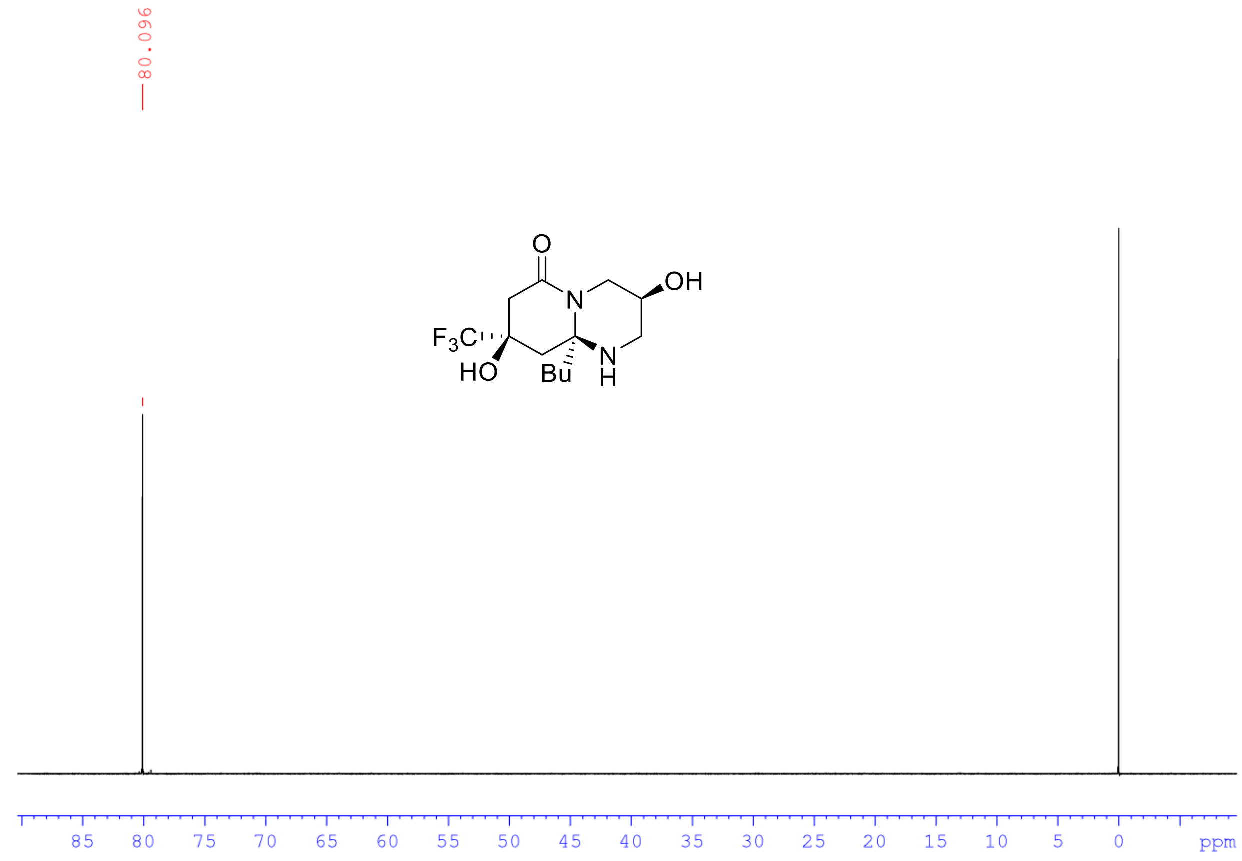

Figure S31: ^{19}F NMR (376 MHz, DMSO-d_6) spectrum of **4c**^{ct}.

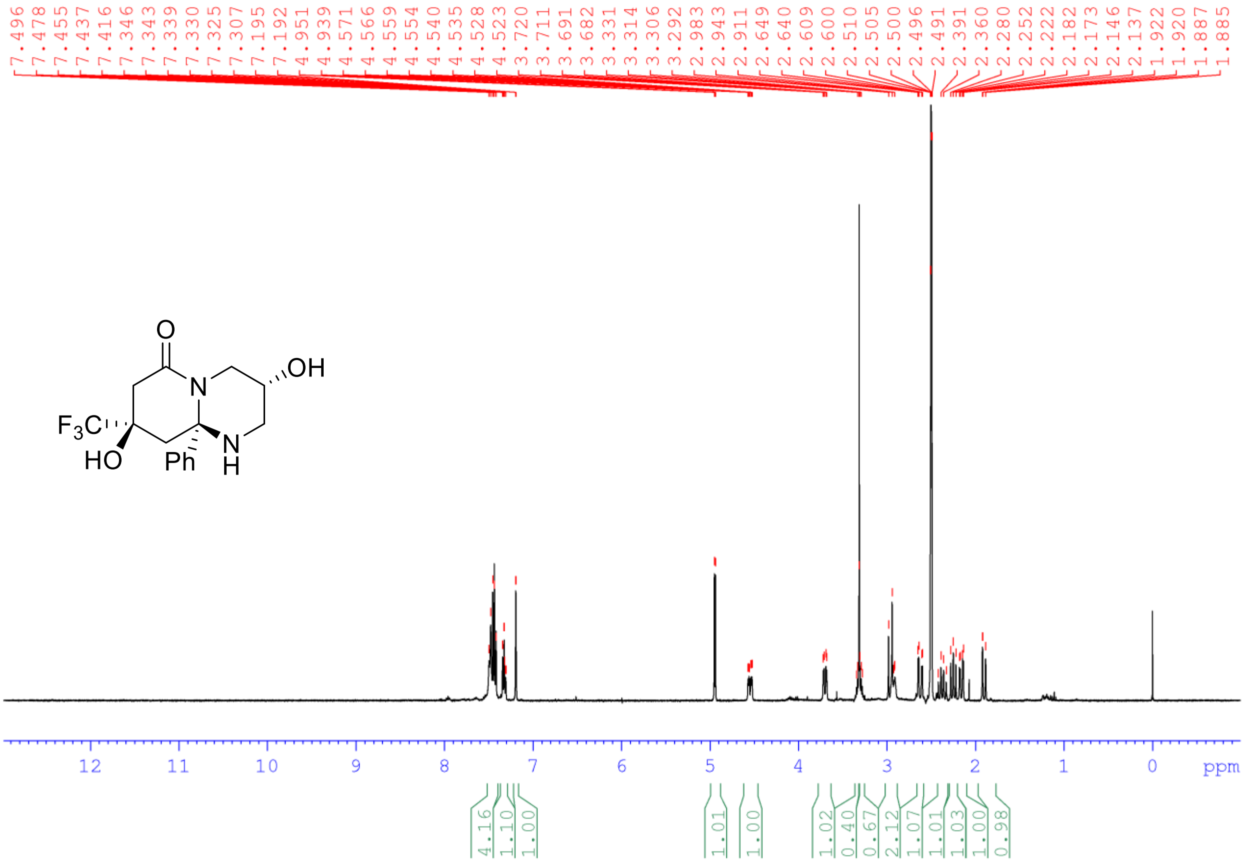

Figure S32: ^{1}H NMR (400 MHz, DMSO-d_6) spectrum of **4d**[cc].

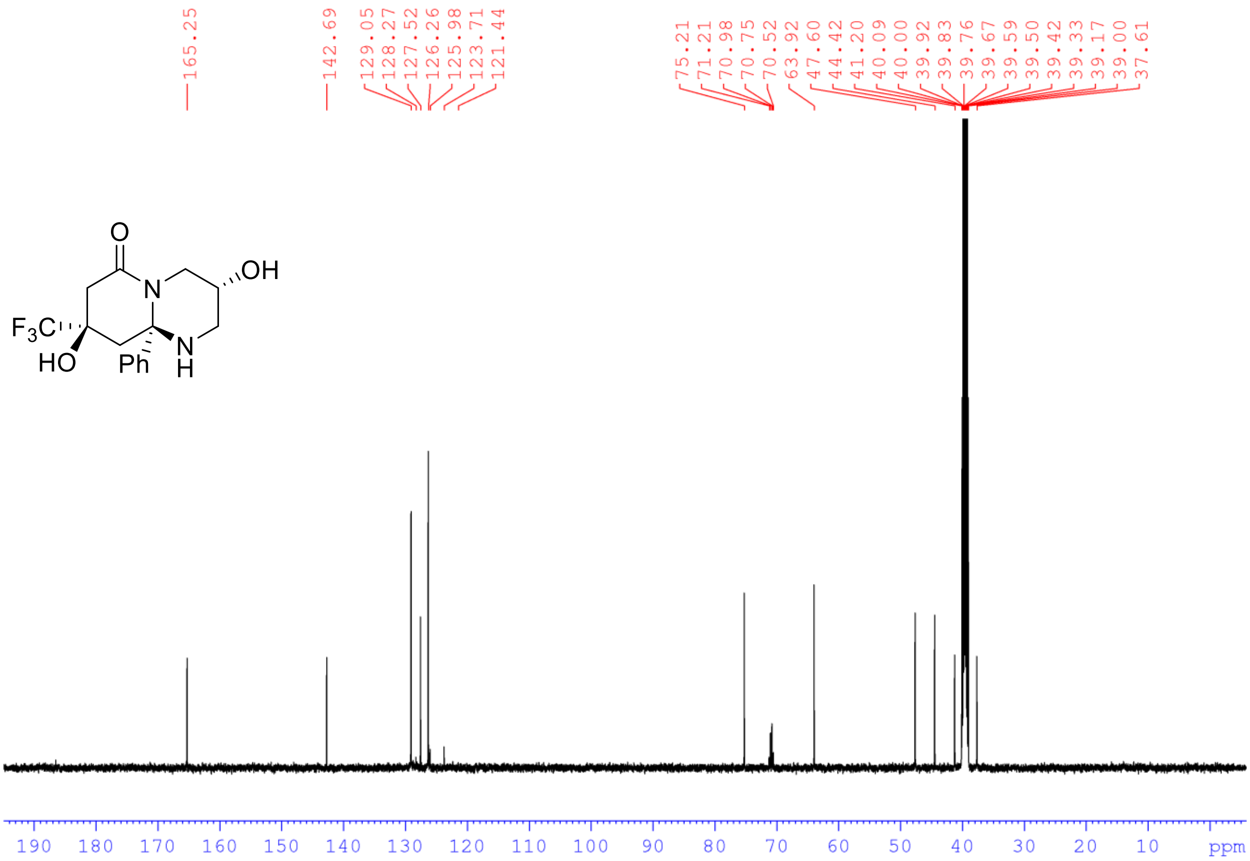

Figure S33: ^{13}C NMR (126 MHz, DMSO-d_6) spectrum of **4d**[cc].

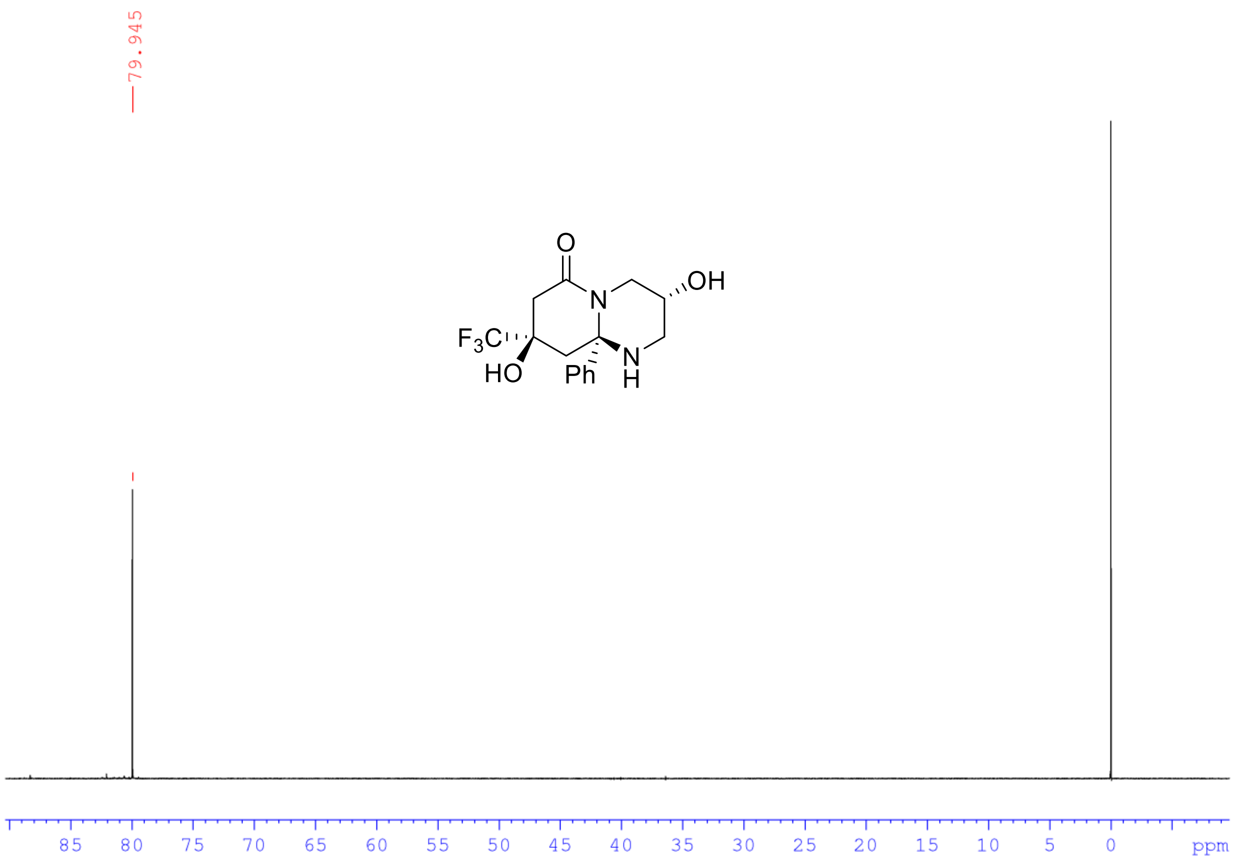

Figure S34: ^{19}F NMR (376 MHz, DMSO-d_6) spectrum of **4d^{cc}**.

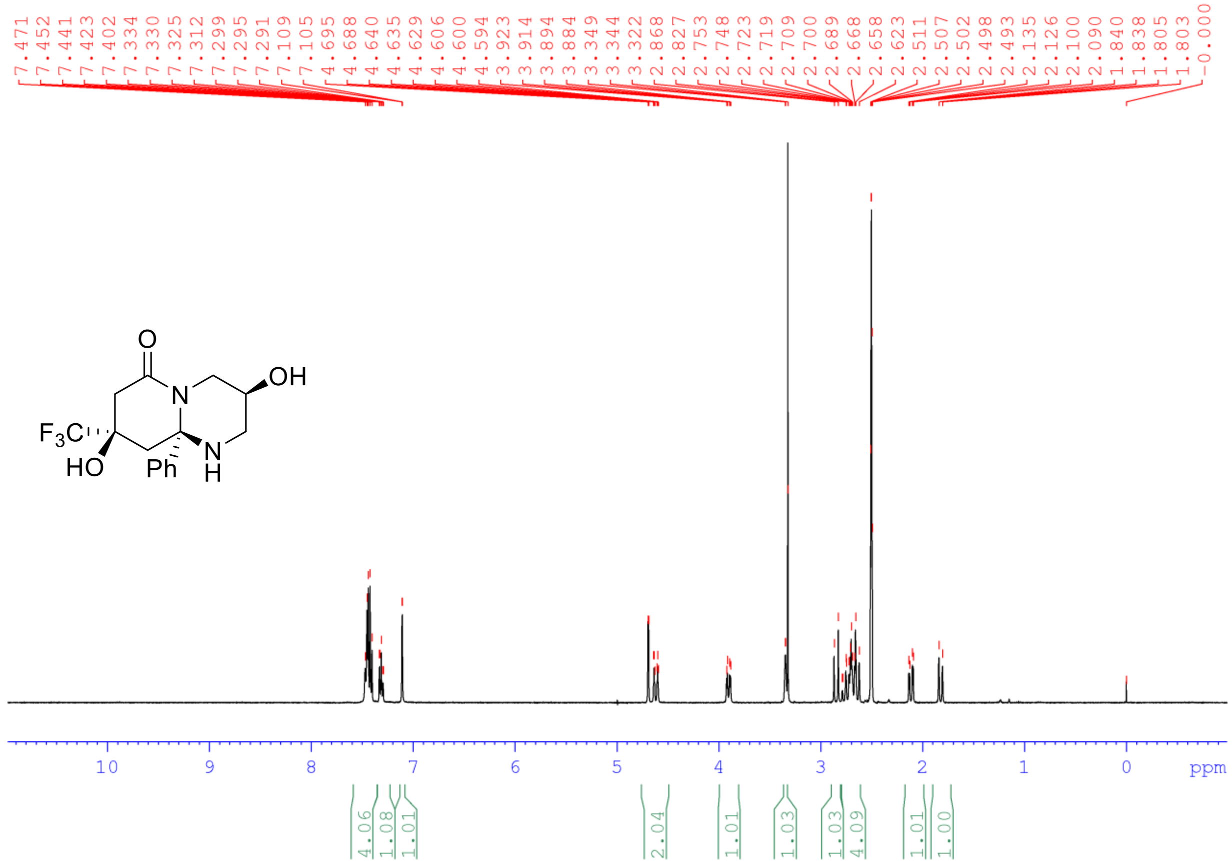

Figure S35: ^{1}H NMR (400 MHz, DMSO-d_6) spectrum of **4d^{ct}**.

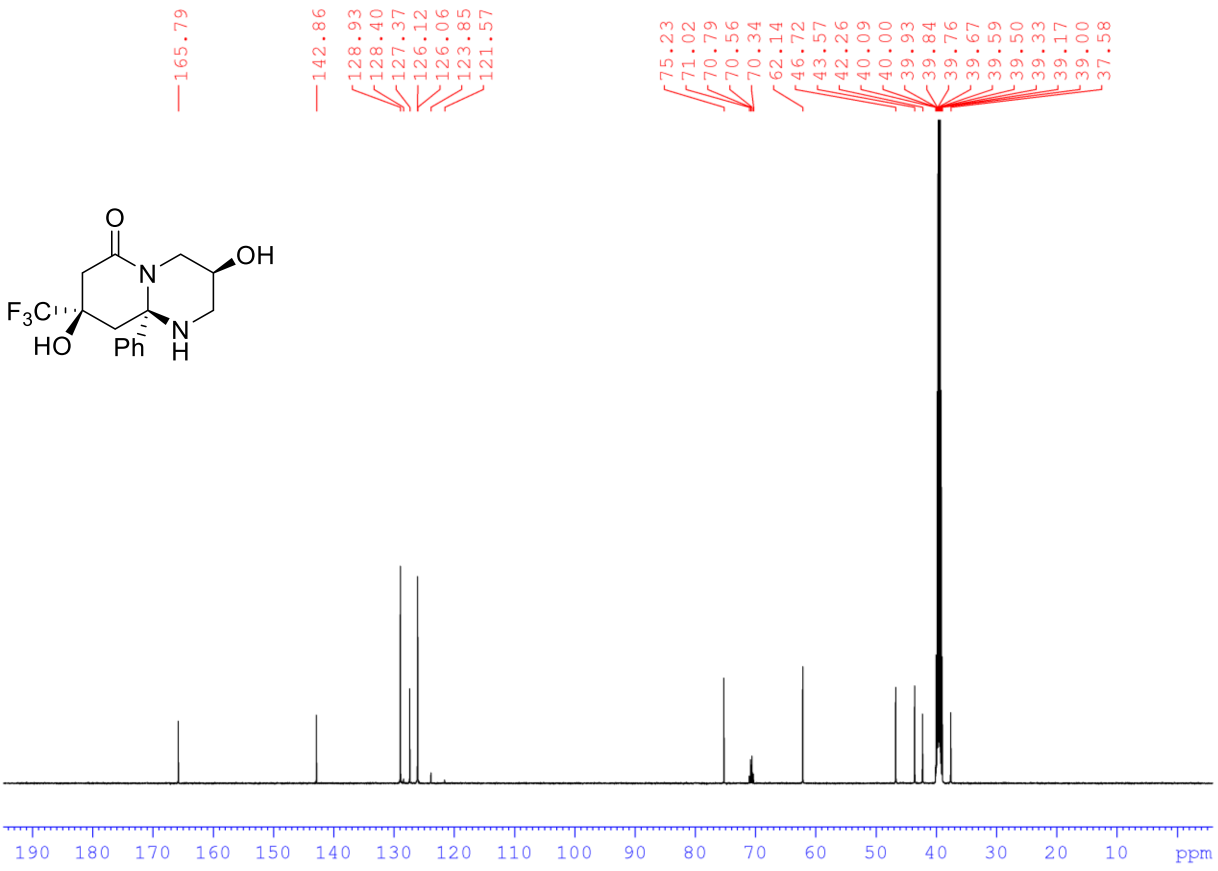

Figure S36: ^{13}C NMR (126 MHz, DMSO-d_6) spectrum of **4d**^{ct}.

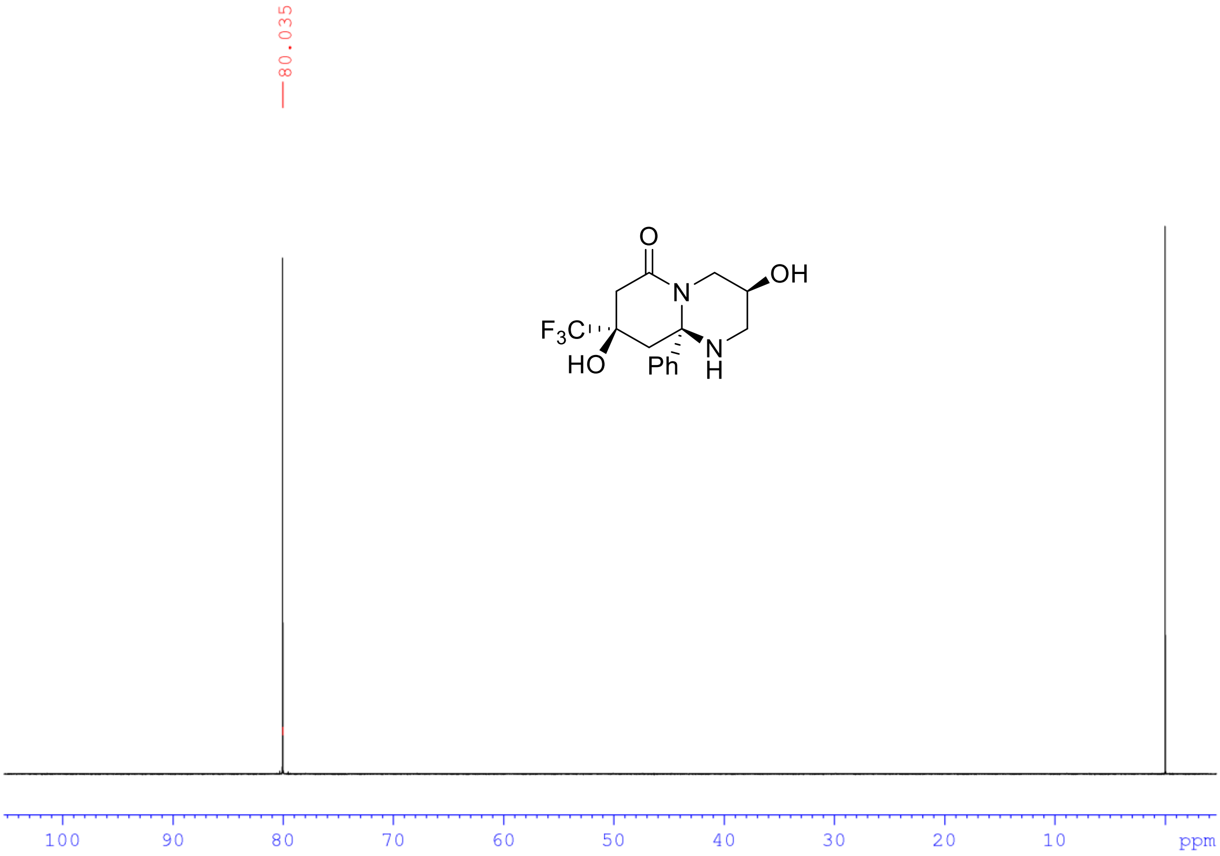

Figure S37: ^{19}F NMR (376 MHz, DMSO-d_6) spectrum of **4d**^{ct}.

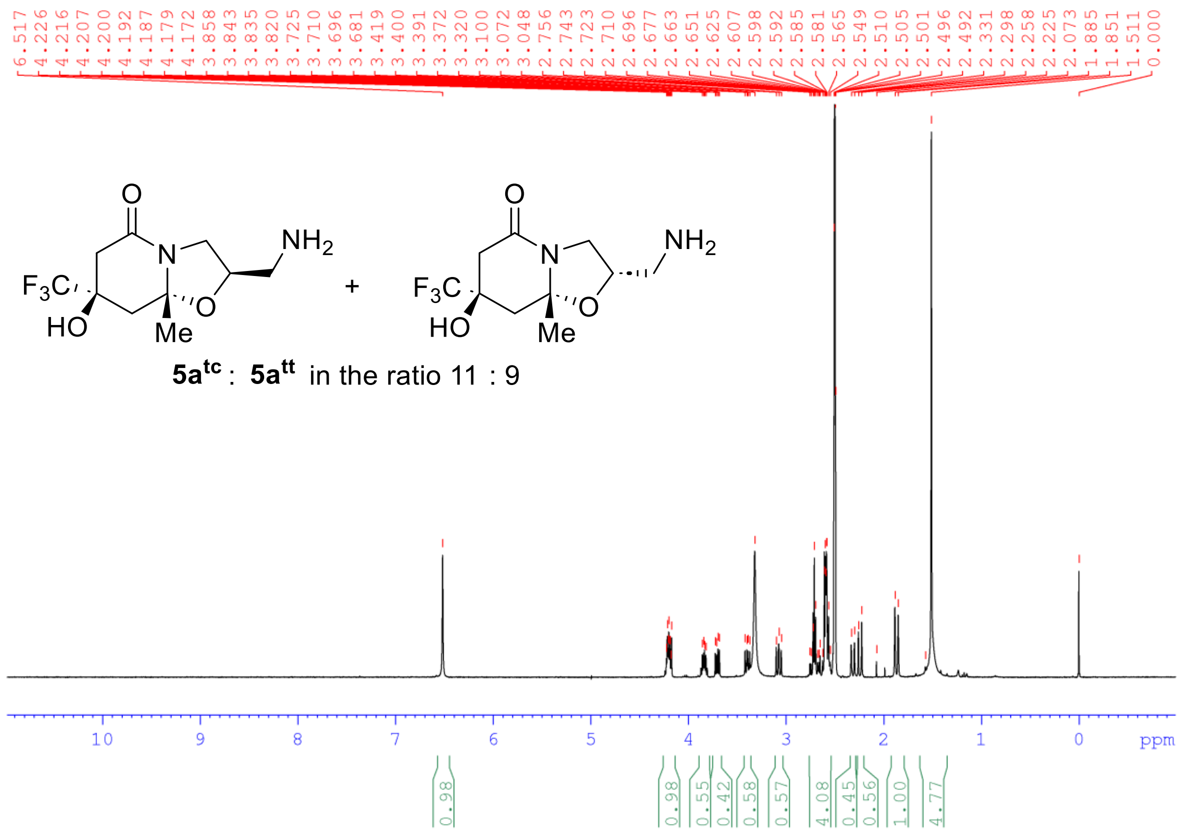

Figure S38: ^{1}H NMR (400 MHz, DMSO-d_6) spectrum of a mixture of diastereomers **5a^{tc}** and **5a^{tt}**.

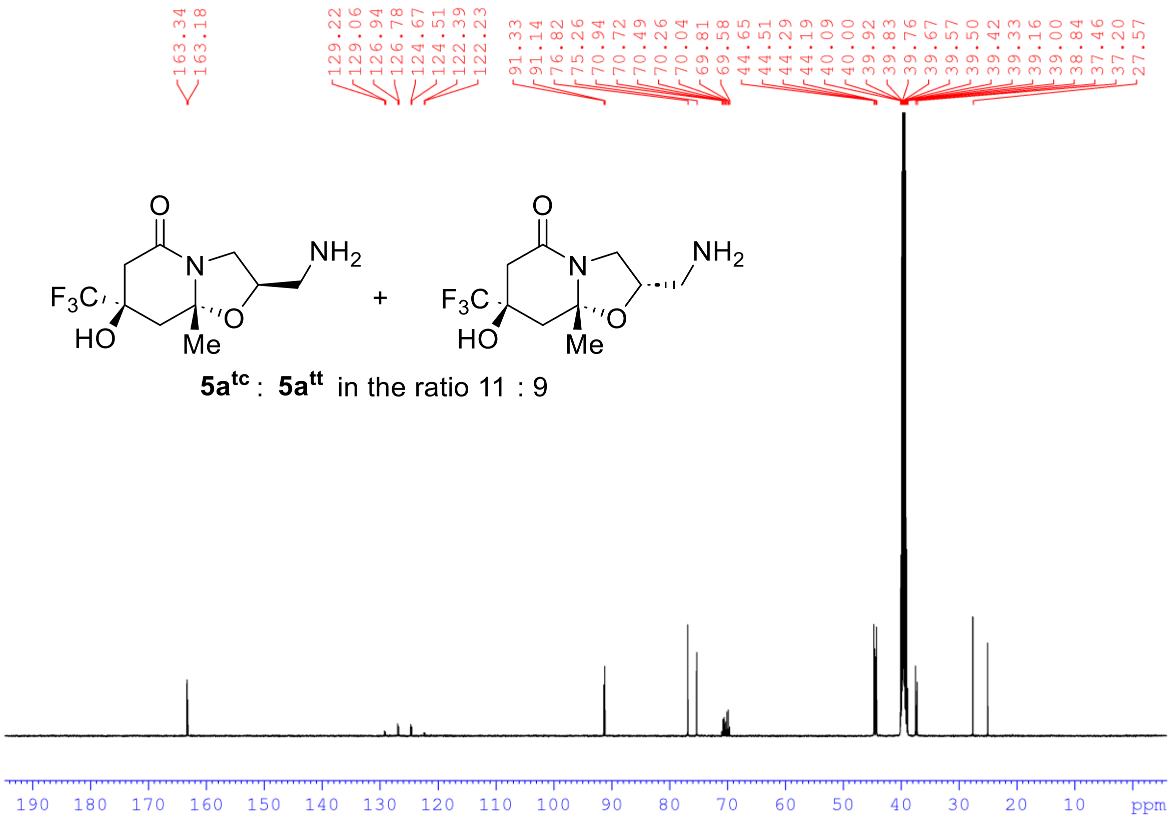

Figure S39: ^{13}C NMR (126 MHz, DMSO-d_6) spectrum of a mixture of diastereomers **5a^{tc}** and **5a^{tt}**.

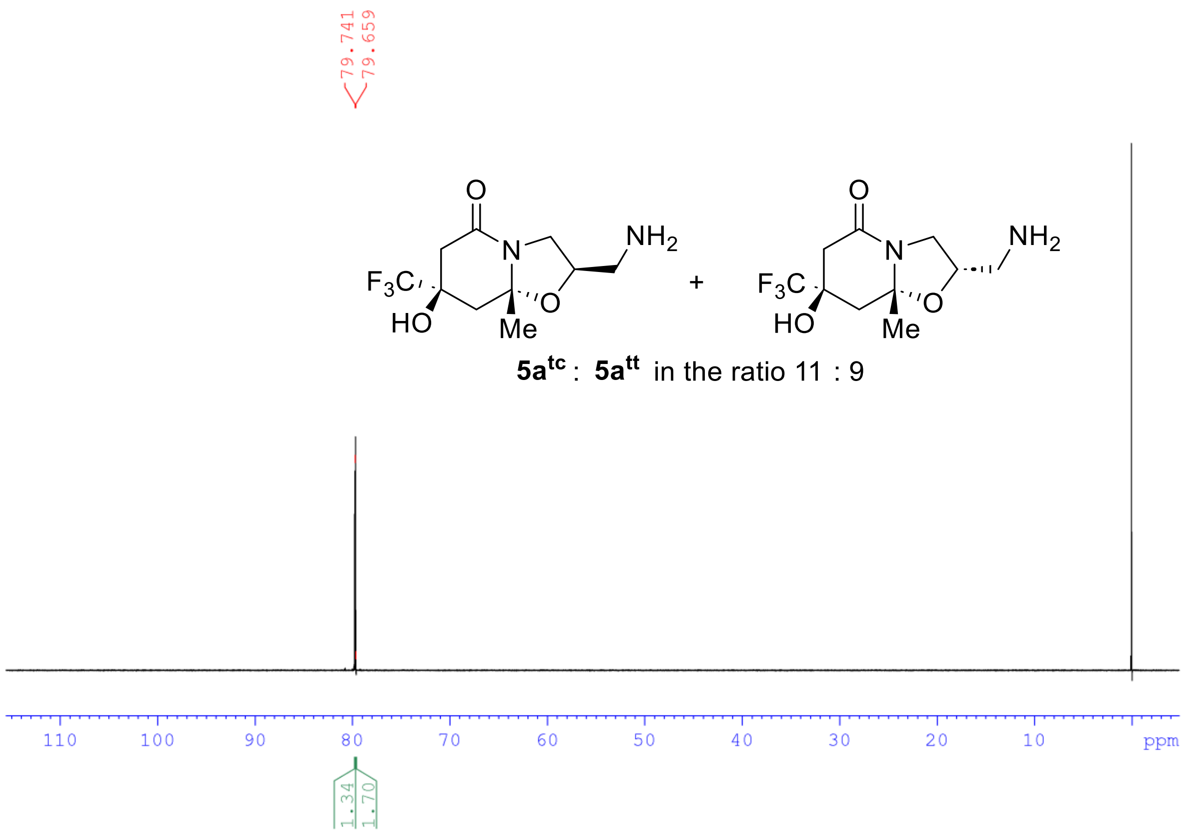

Figure S40: ^{19}F NMR (376 MHz, DMSO-d_6) spectrum of a mixture of diastereomers **5a^{tc}** and **5a^{tt}**.

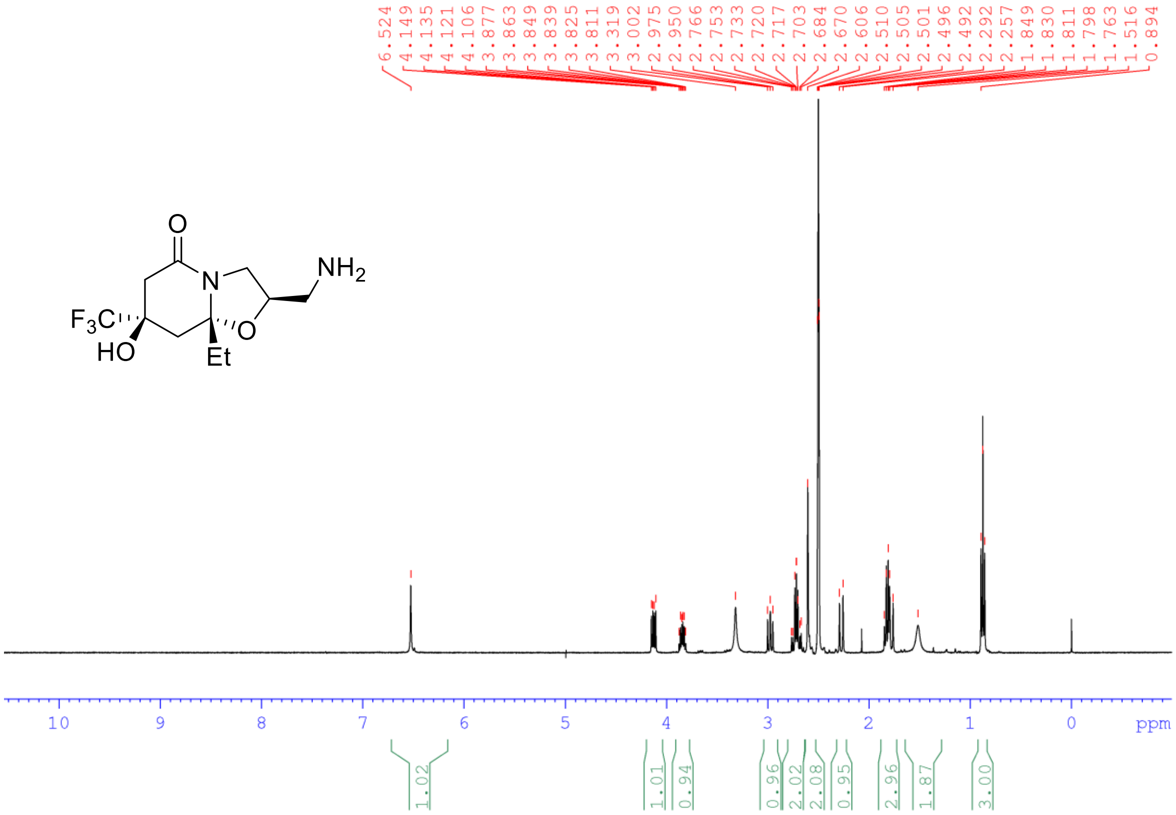

Figure S41: ^{1}H NMR (400 MHz, DMSO-d_6) spectrum of **5b^{tc}**.

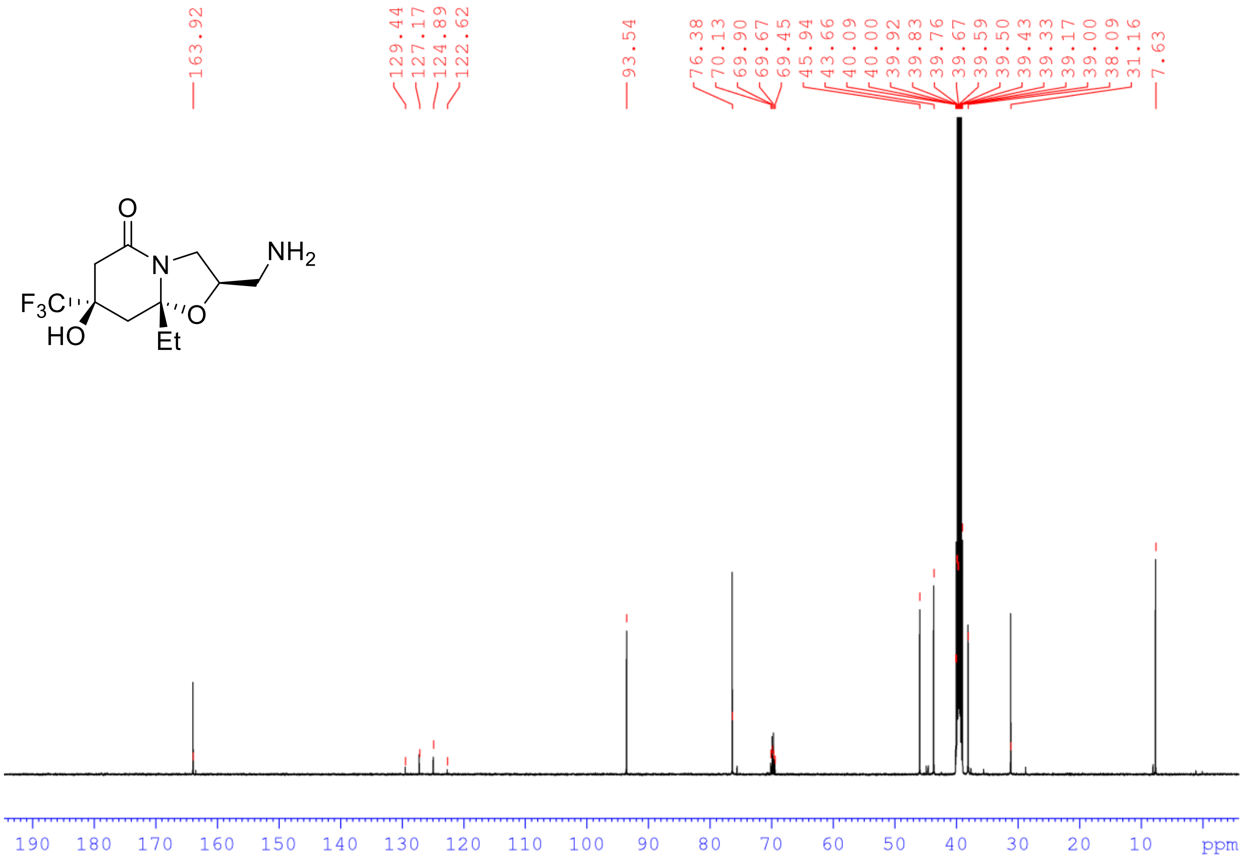

Figure S42: ^{13}C NMR (126 MHz, DMSO-*d*$_6$) spectrum of **5b**^{tc}.

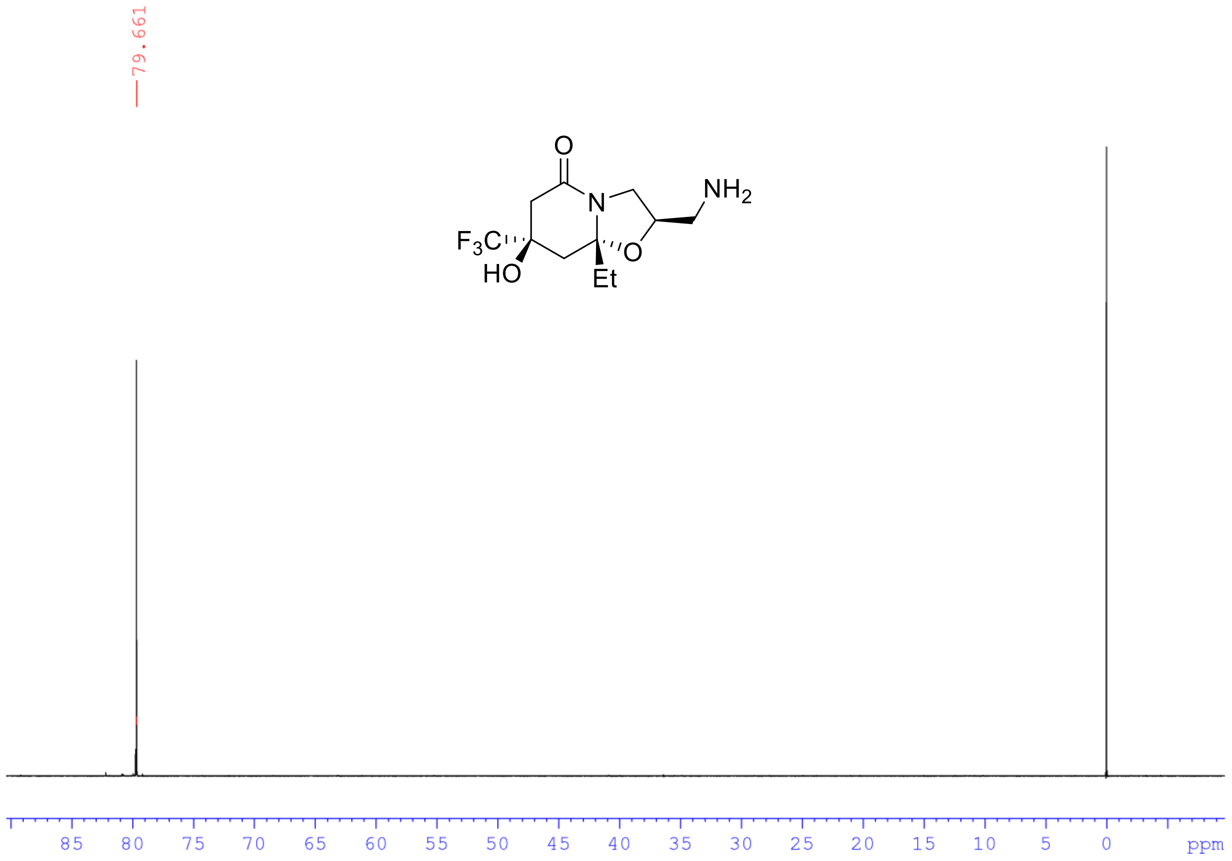

Figure S43: ^{19}F NMR (376 MHz, DMSO-*d*$_6$) spectrum of **5b**^{tc}.

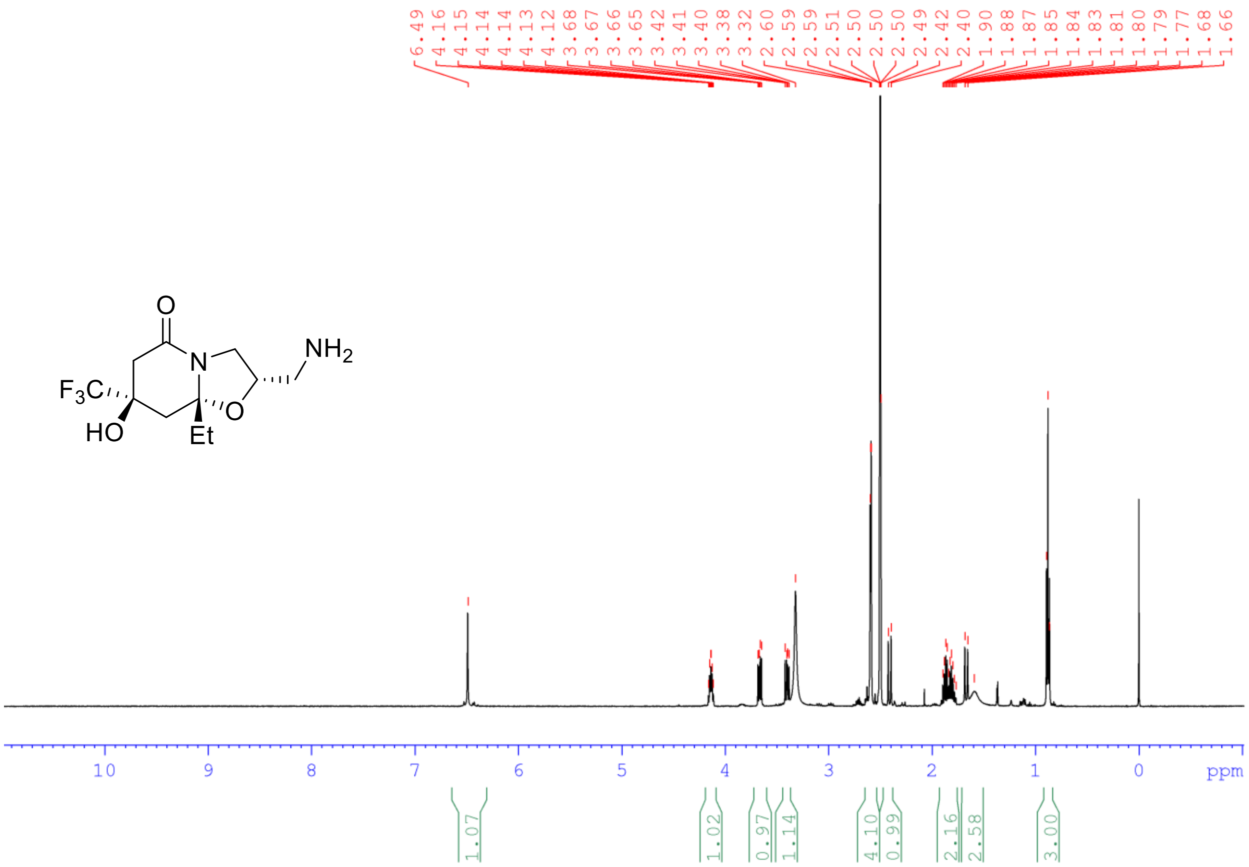

Figure S44: ^{1}H NMR (500 MHz, DMSO-d_6) spectrum of **5b^{tt}**.

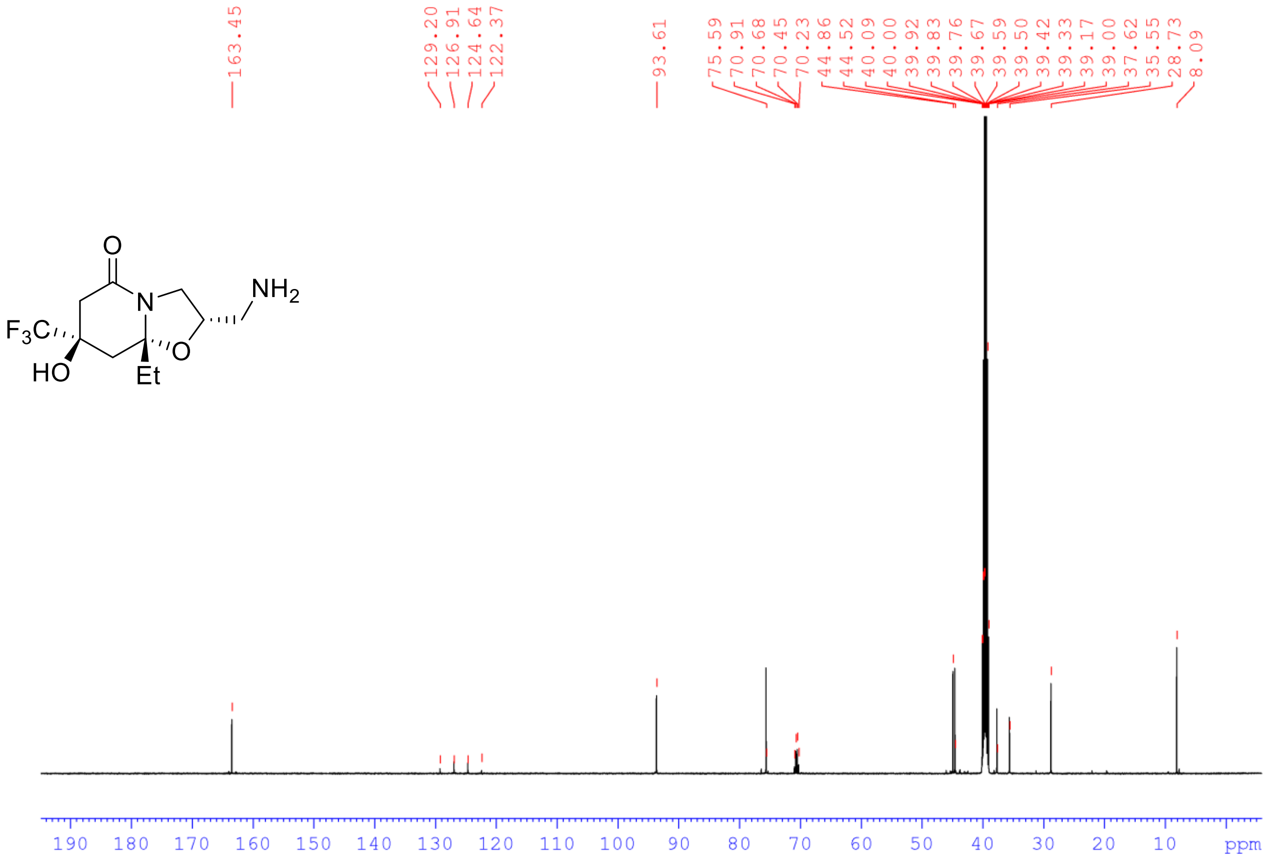

Figure S45: ^{13}C NMR (126 MHz, DMSO-d_6) spectrum of **5b^{tt}**.

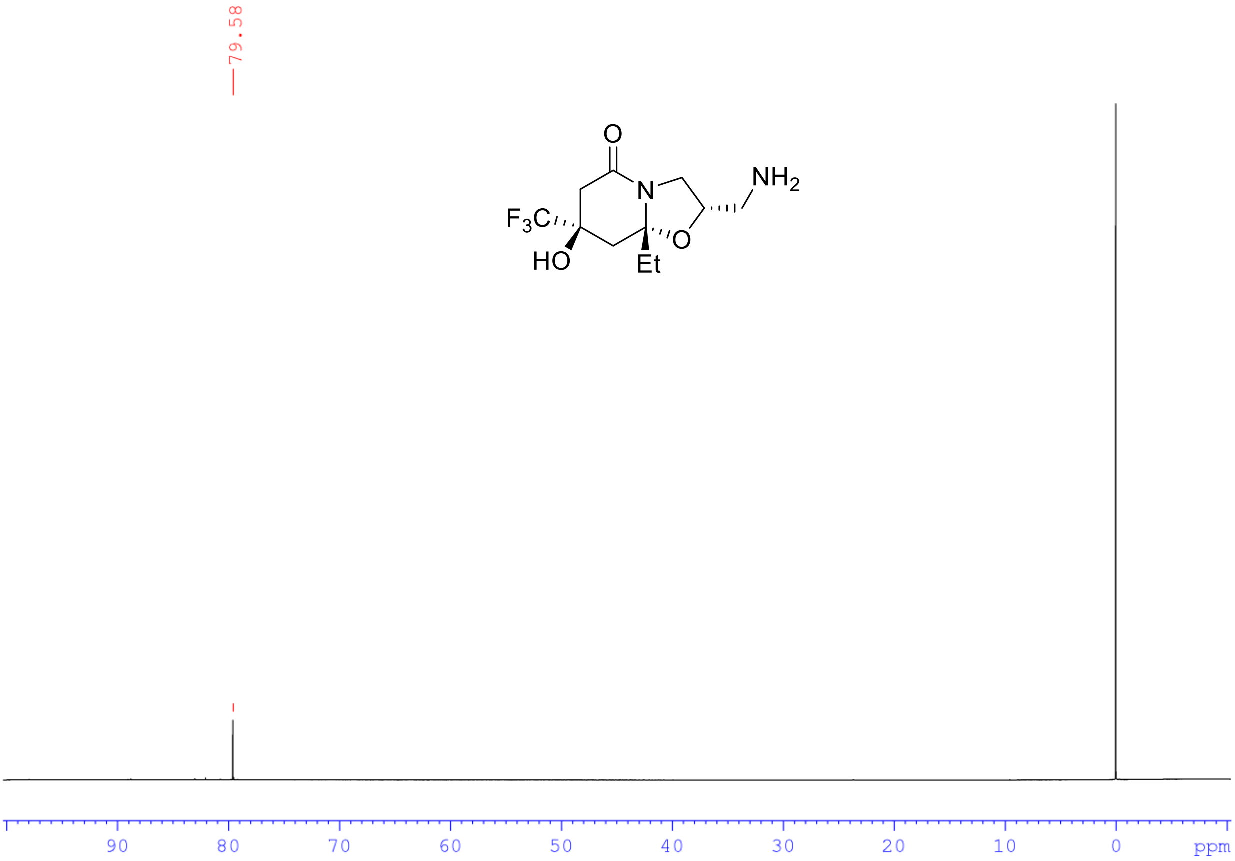

Figure S46: ^{19}F NMR (470 MHz, DMSO-d_6) spectrum of **5b**$^{\mathbf{tt}}$.

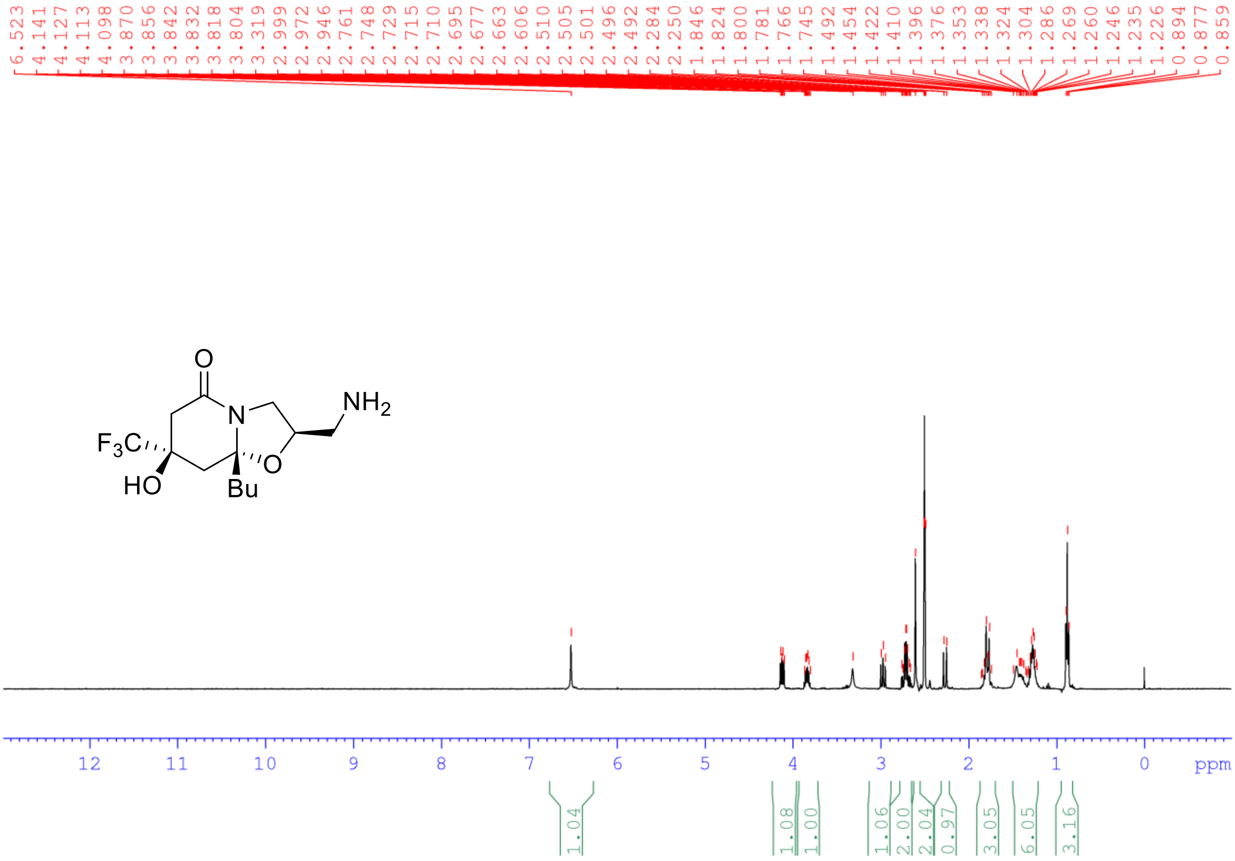

Figure S47: ^{1}H NMR (400 MHz, DMSO-d_6) spectrum of **5c**$^{\mathbf{tc}}$.

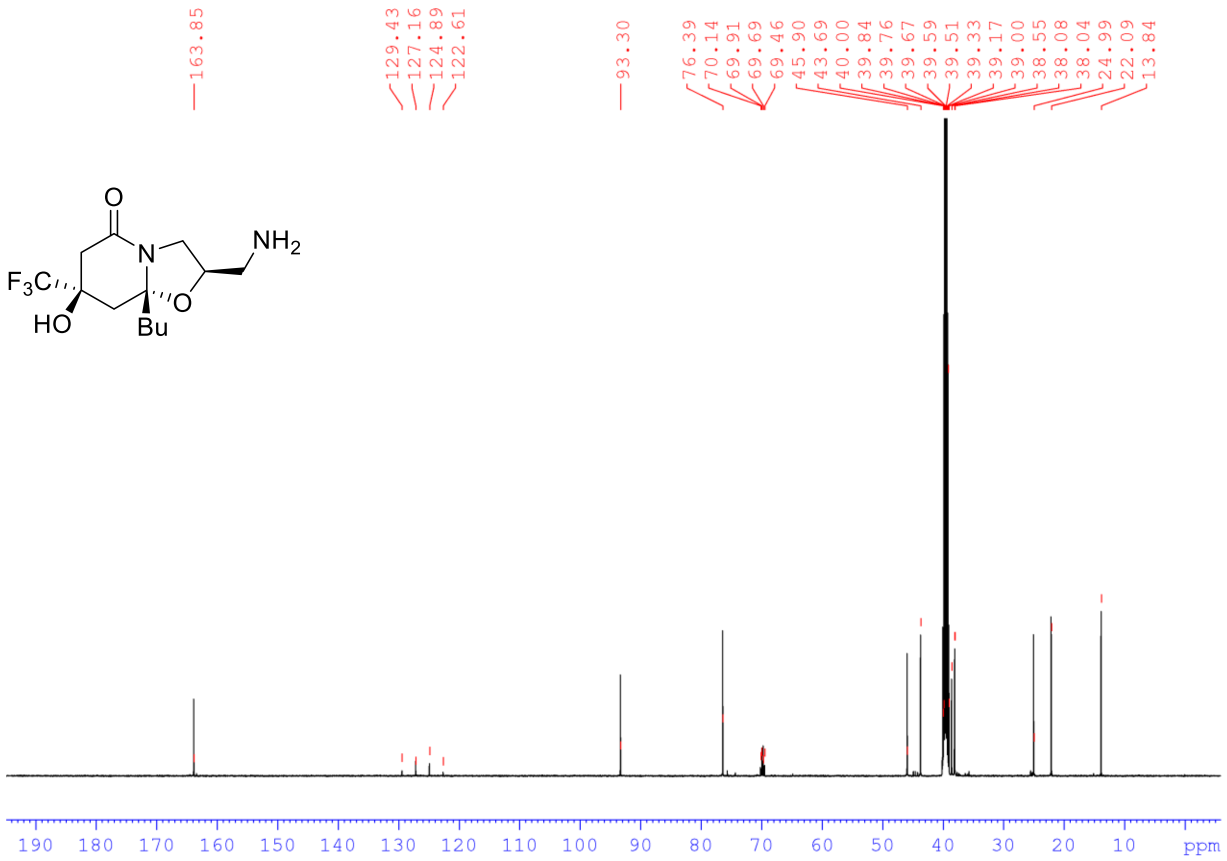

Figure S48: ^{13}C NMR (126 MHz, DMSO-*d*$_6$) spectrum of **5c^{tc}**.

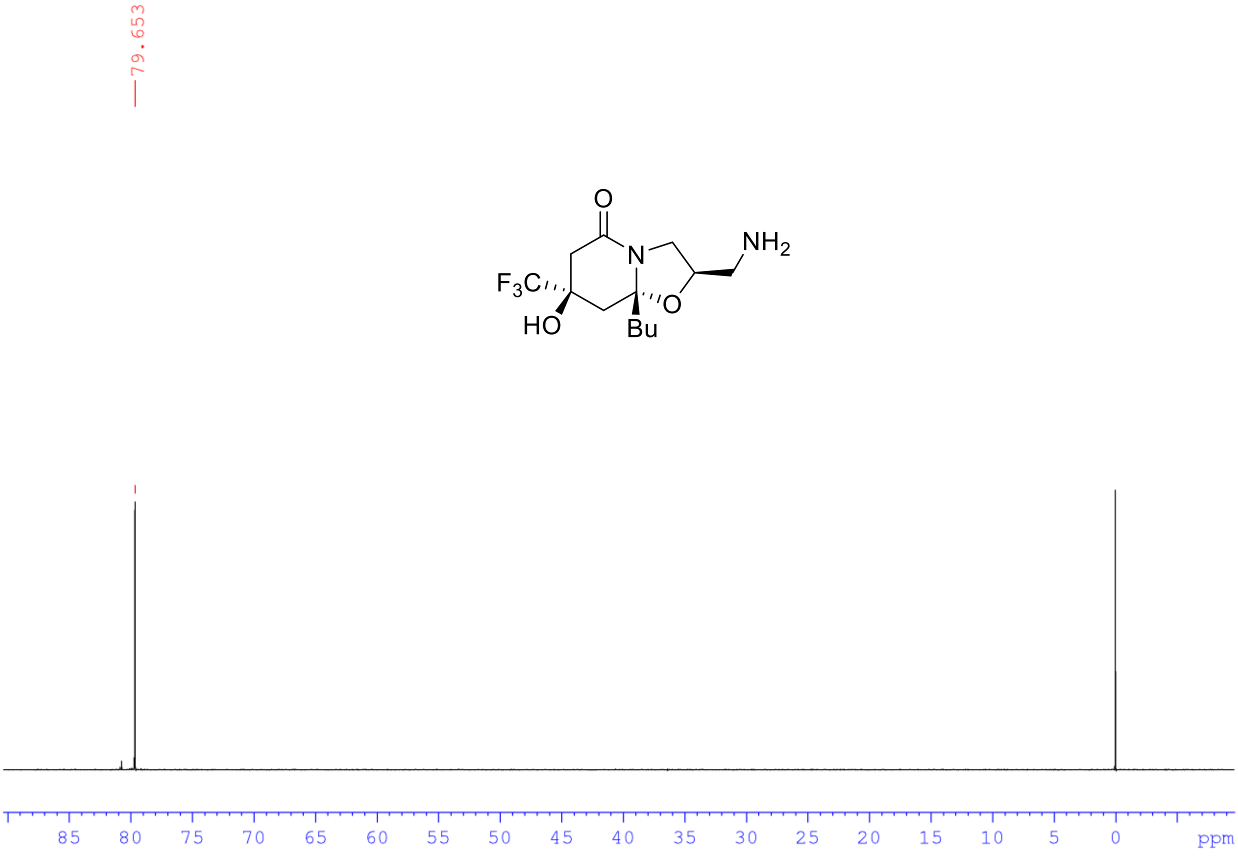

Figure S49: ^{19}F NMR (376 MHz, DMSO-*d*$_6$) spectrum of **5c^{tc}**.

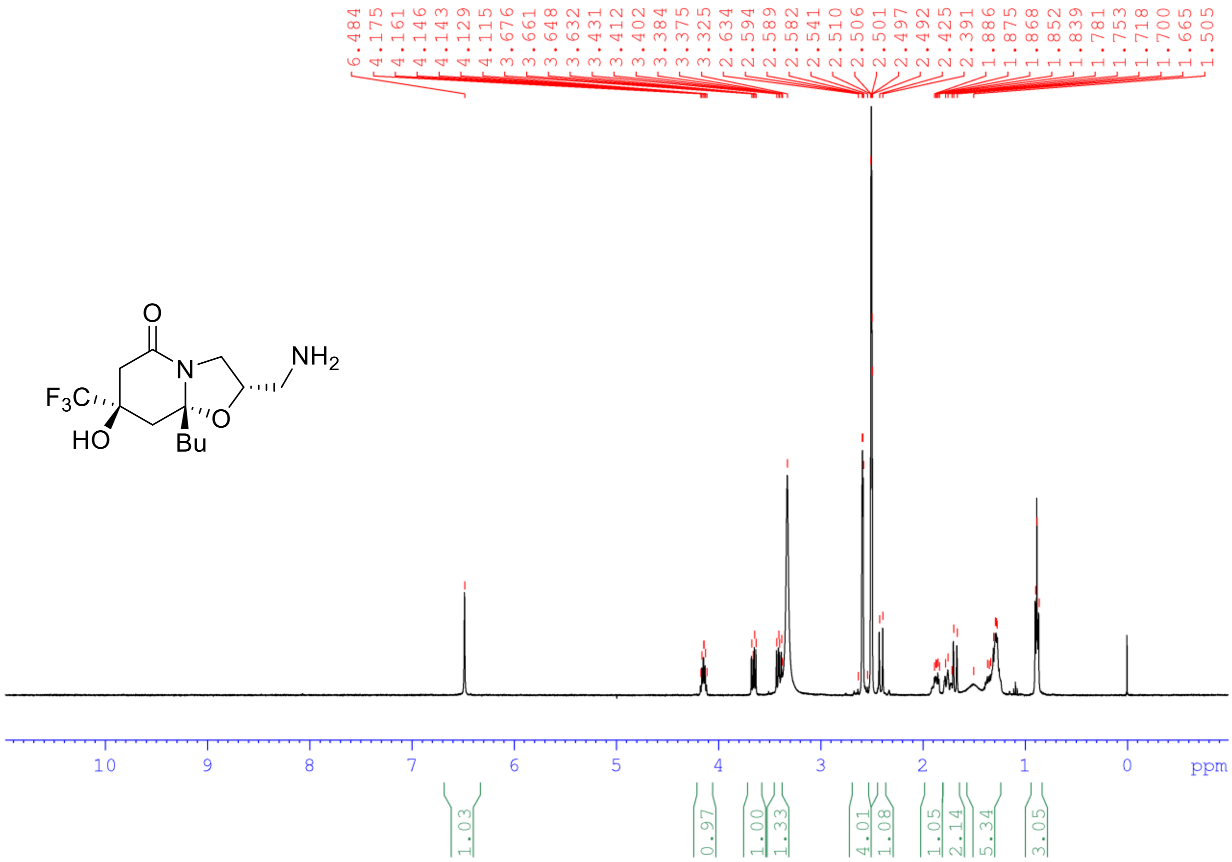

Figure S50: ^{1}H NMR (400 MHz, DMSO-d_6) spectrum of **5c**^{tt}.

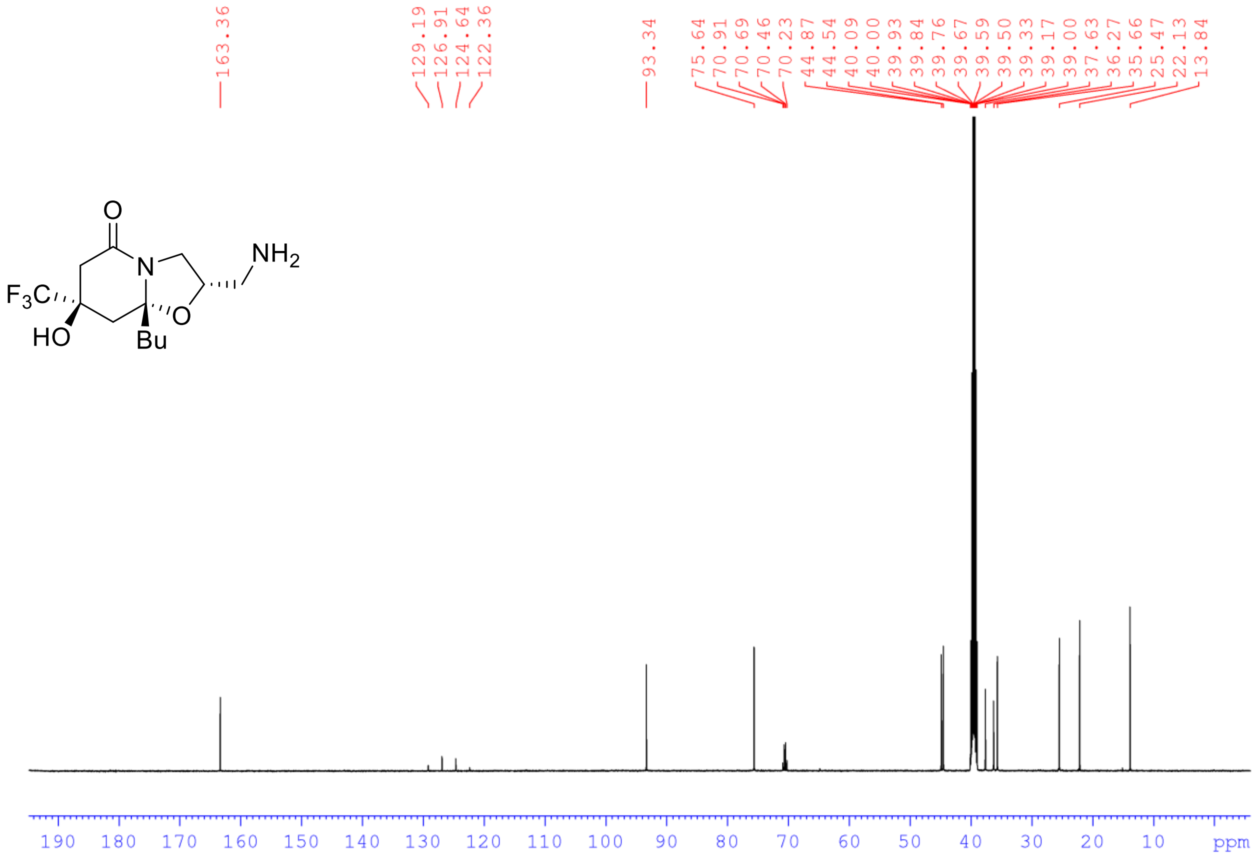

Figure S51: ^{13}C NMR (126 MHz, DMSO-d_6) spectrum of **5c**^{tt}.

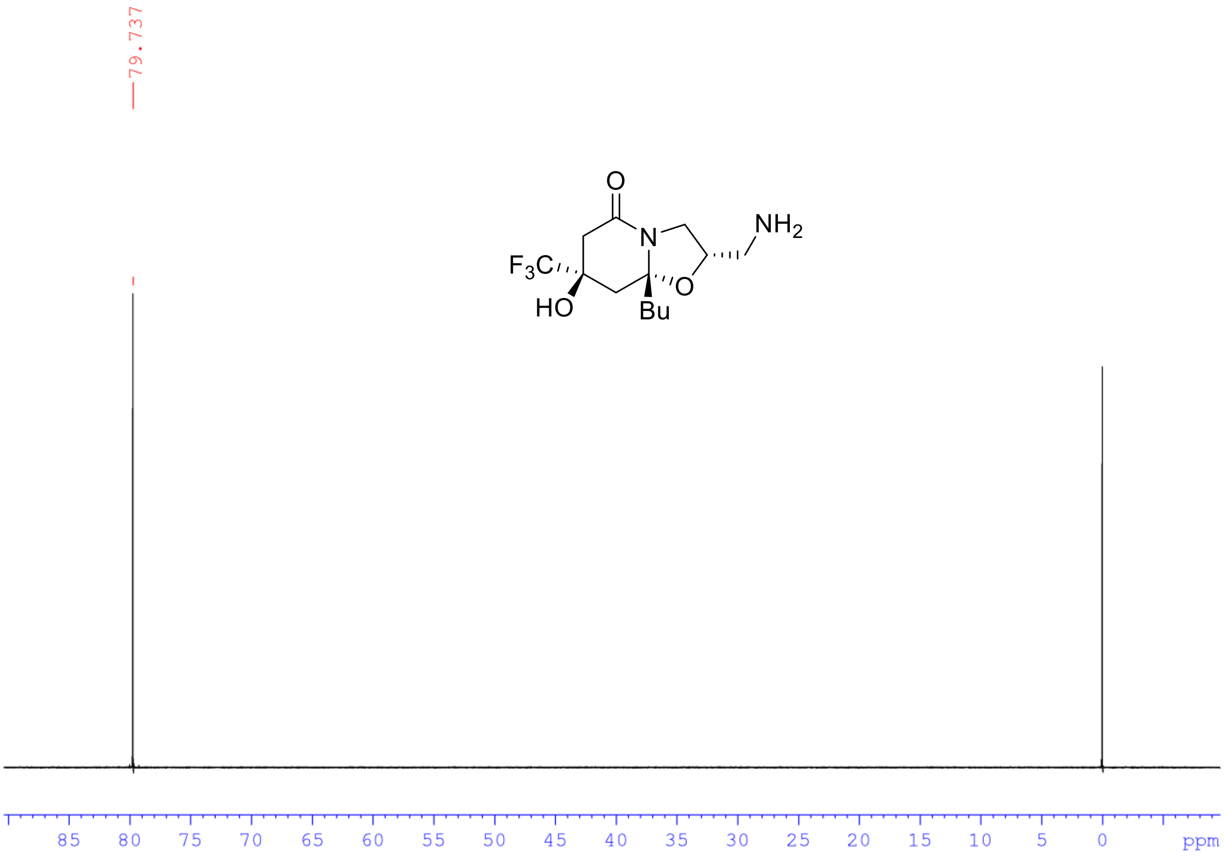

Figure S52: ^{19}F NMR (376 MHz, DMSO-d_6) spectrum of **5c^{tt}**.

Copies of IR spectra

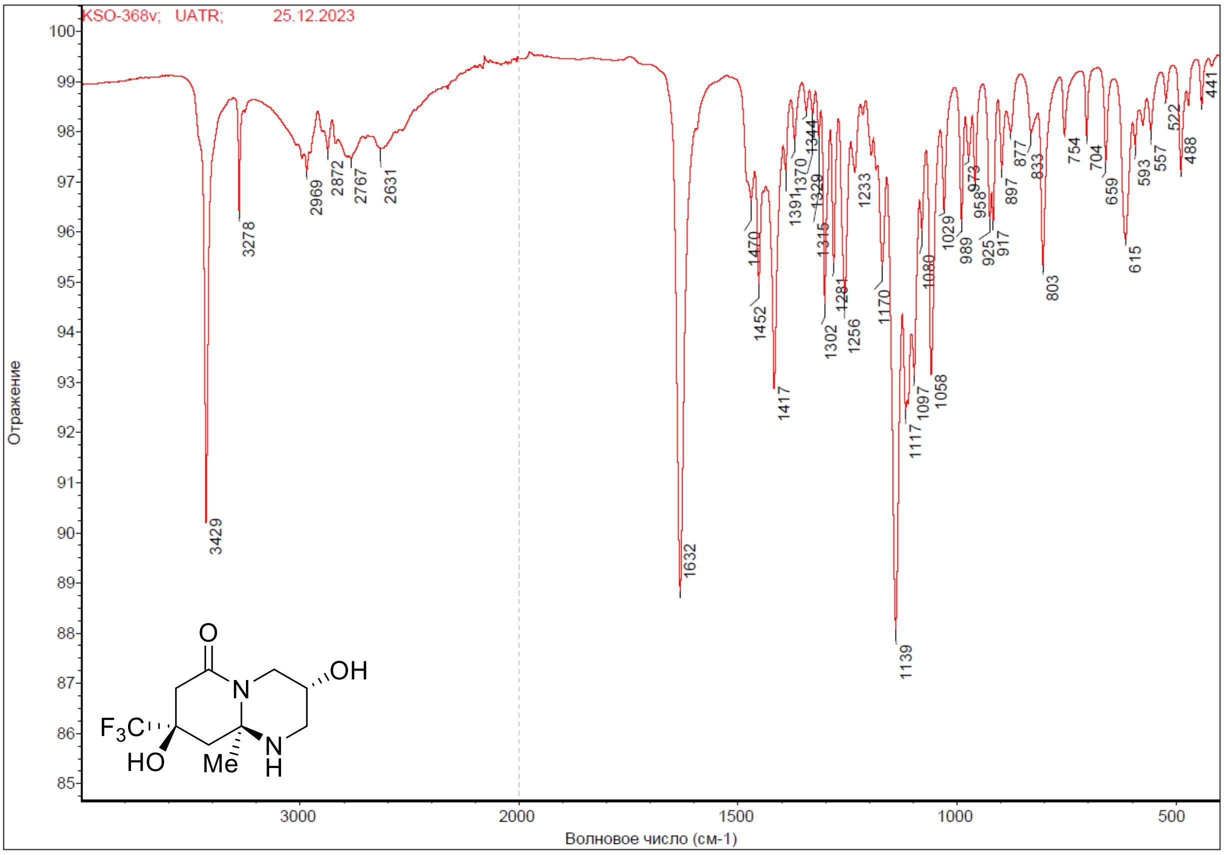

Figure S53: IR spectrum of **4a^{cc}**.

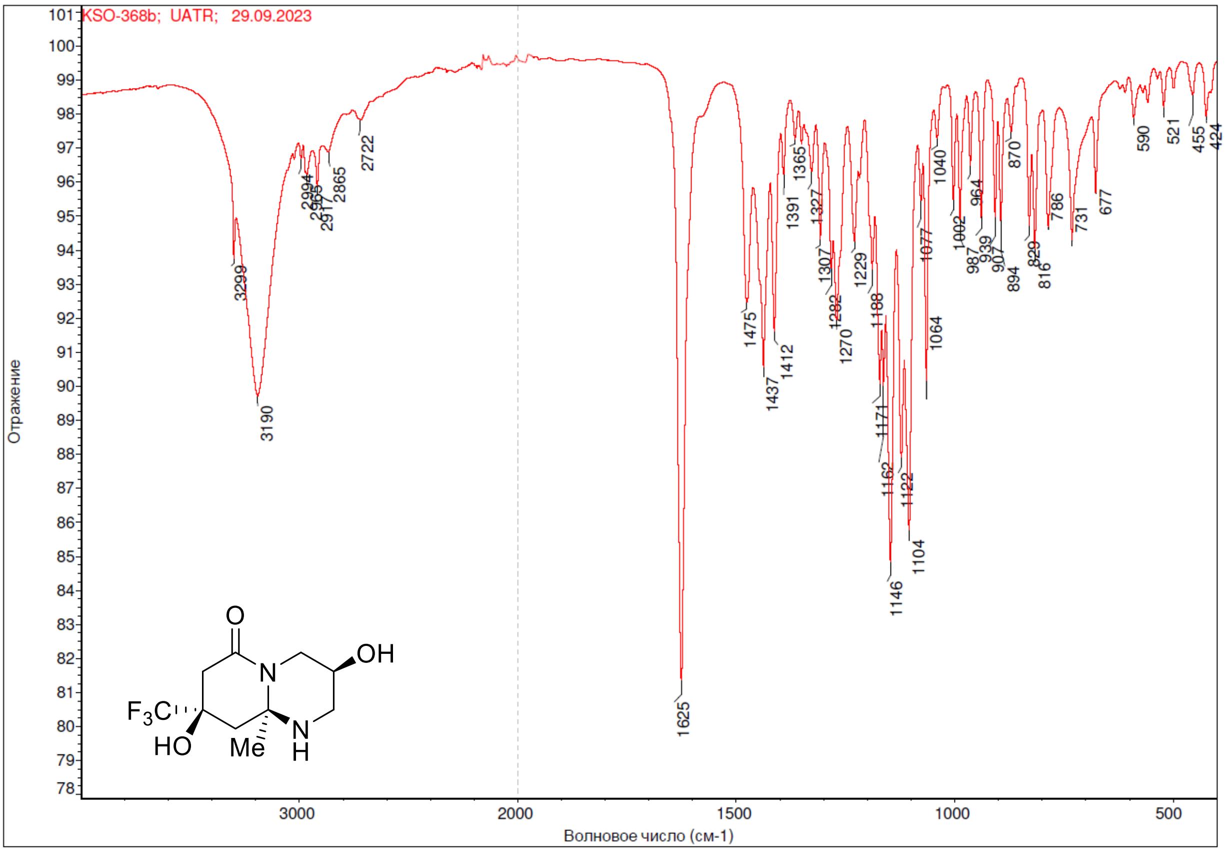

Figure S54: IR spectrum of **4a**^{ct}.

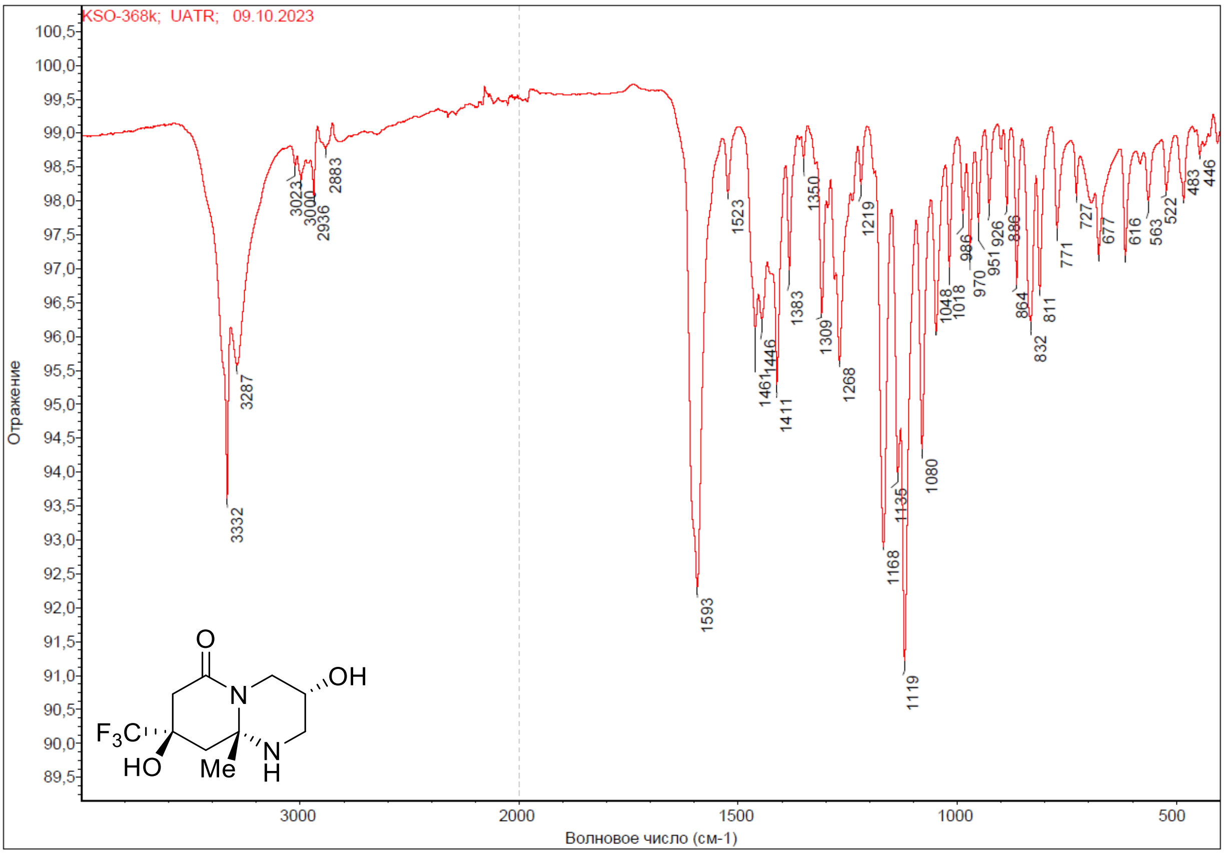

Figure S55: IR spectrum of **4a**^{tt}.

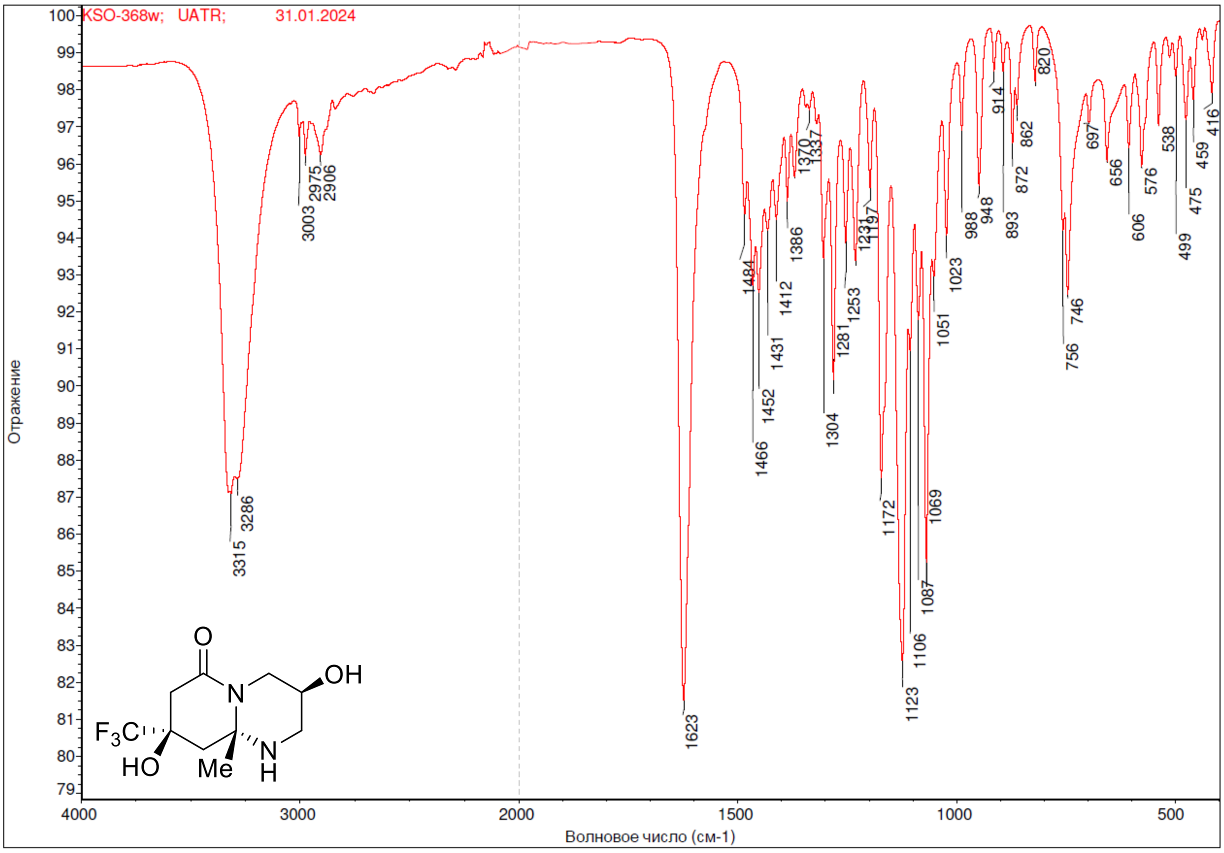

Figure S56: IR spectrum of **4a**^{tc}.

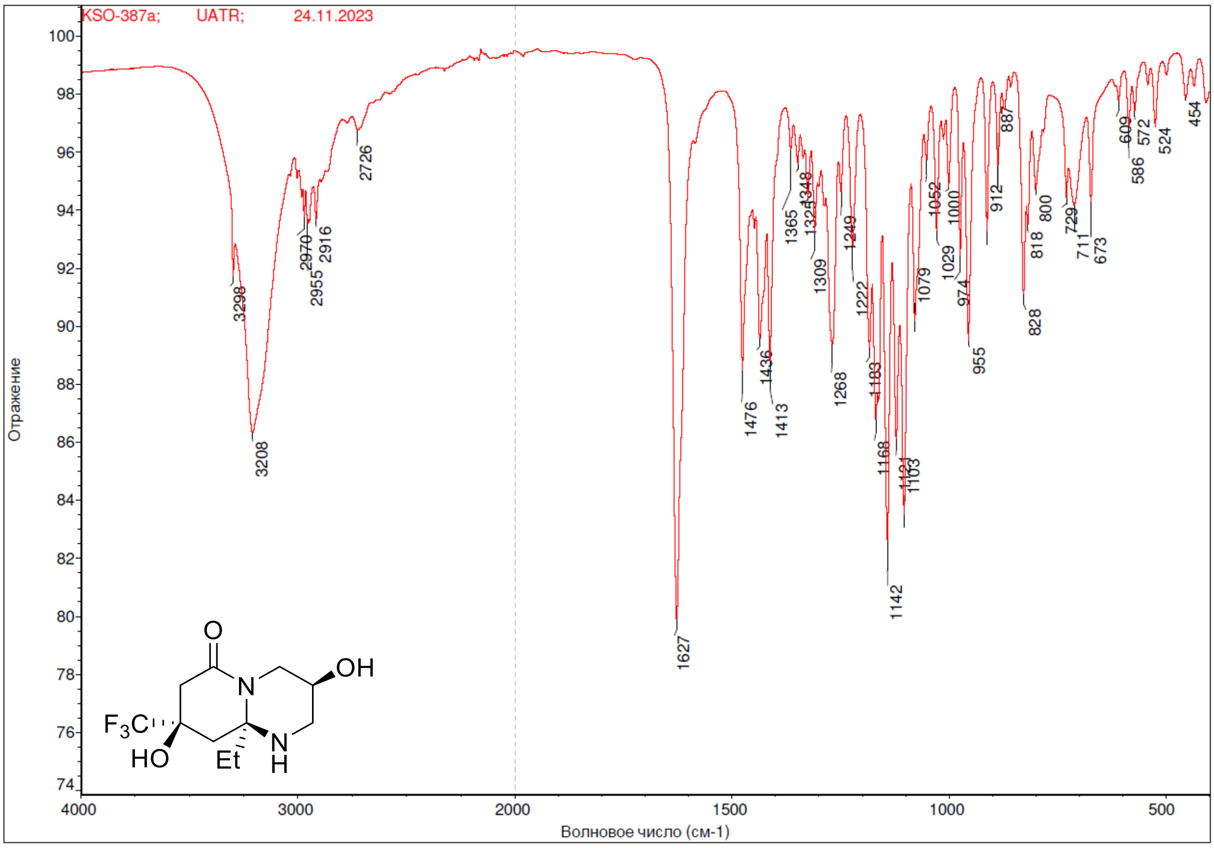

Figure S57: IR spectrum of **4b**^{ct}.

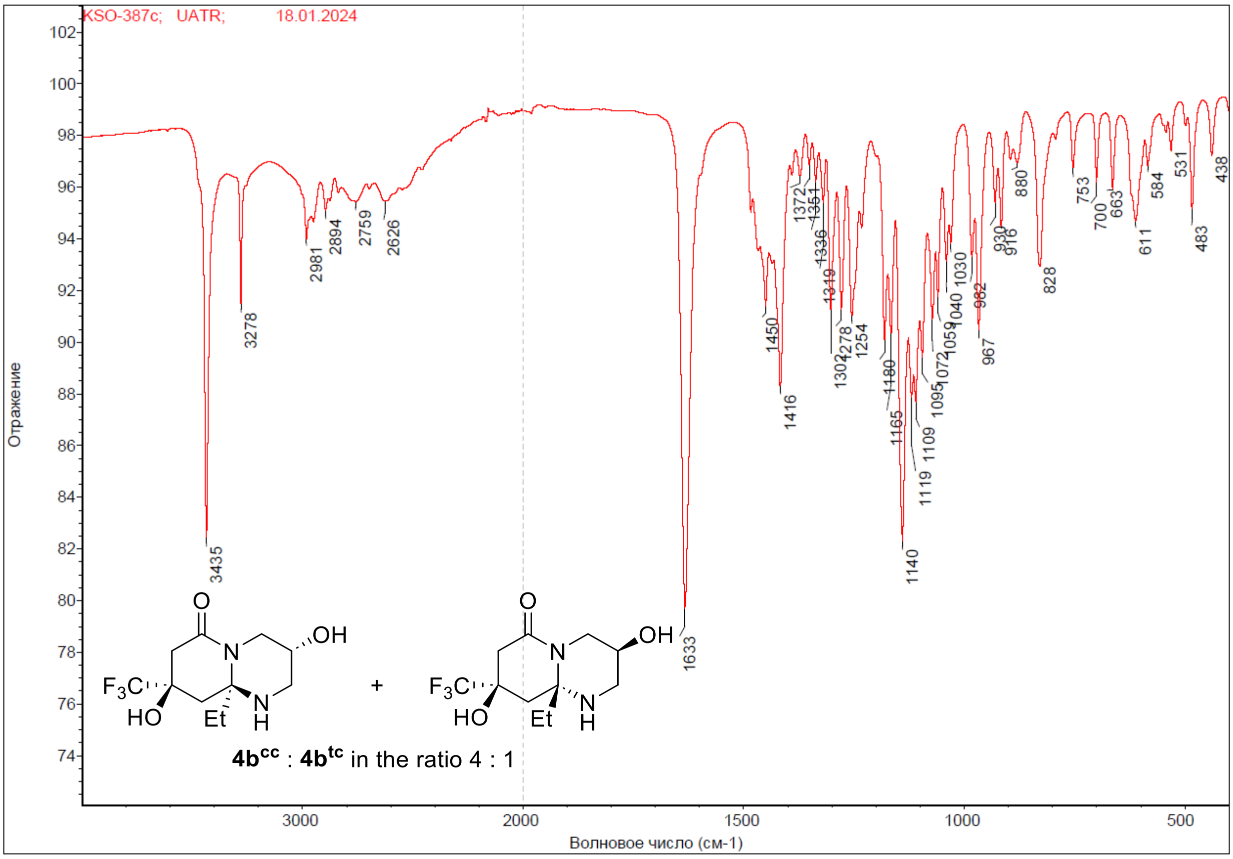

Figure S58: IR spectrum of a mixture of diastereomers **4b**^{cc} and **4b**^{tc}.

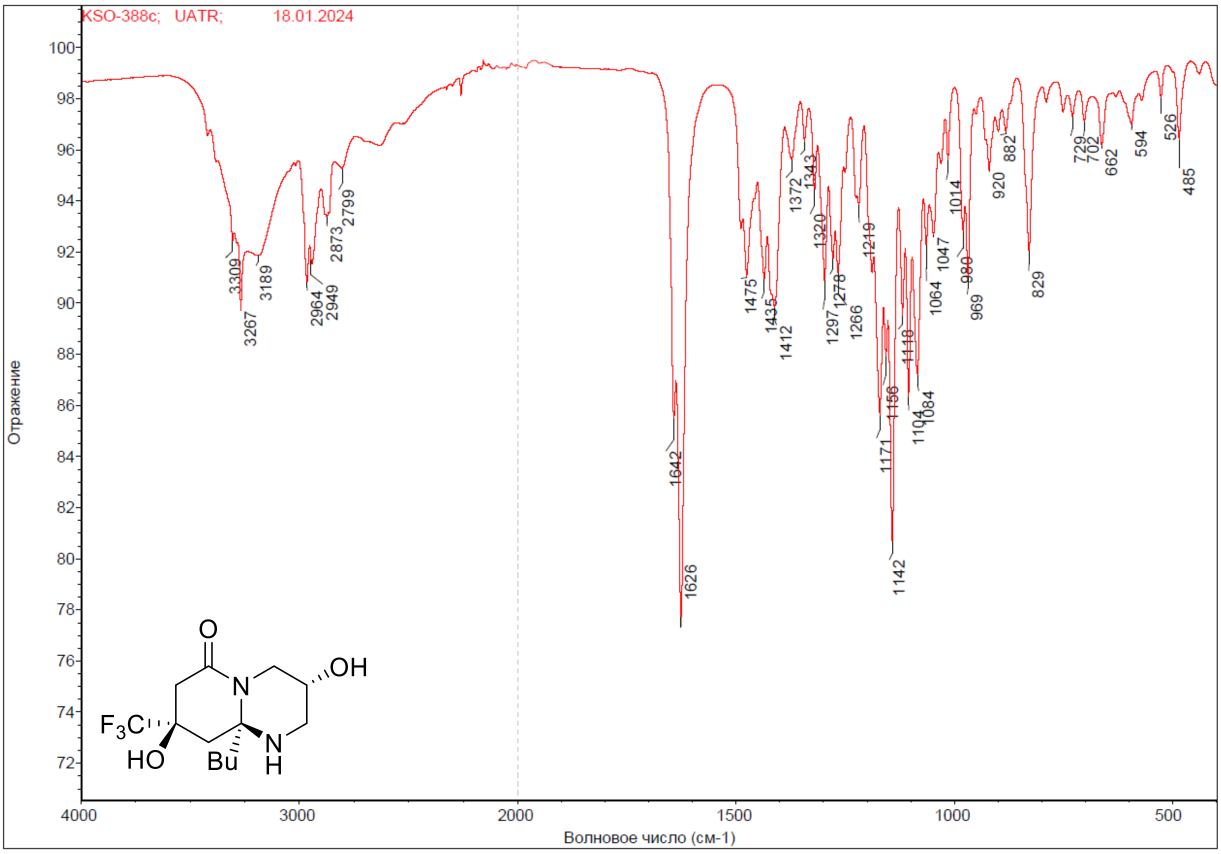

Figure S59: IR spectrum of **4c**^{cc}.

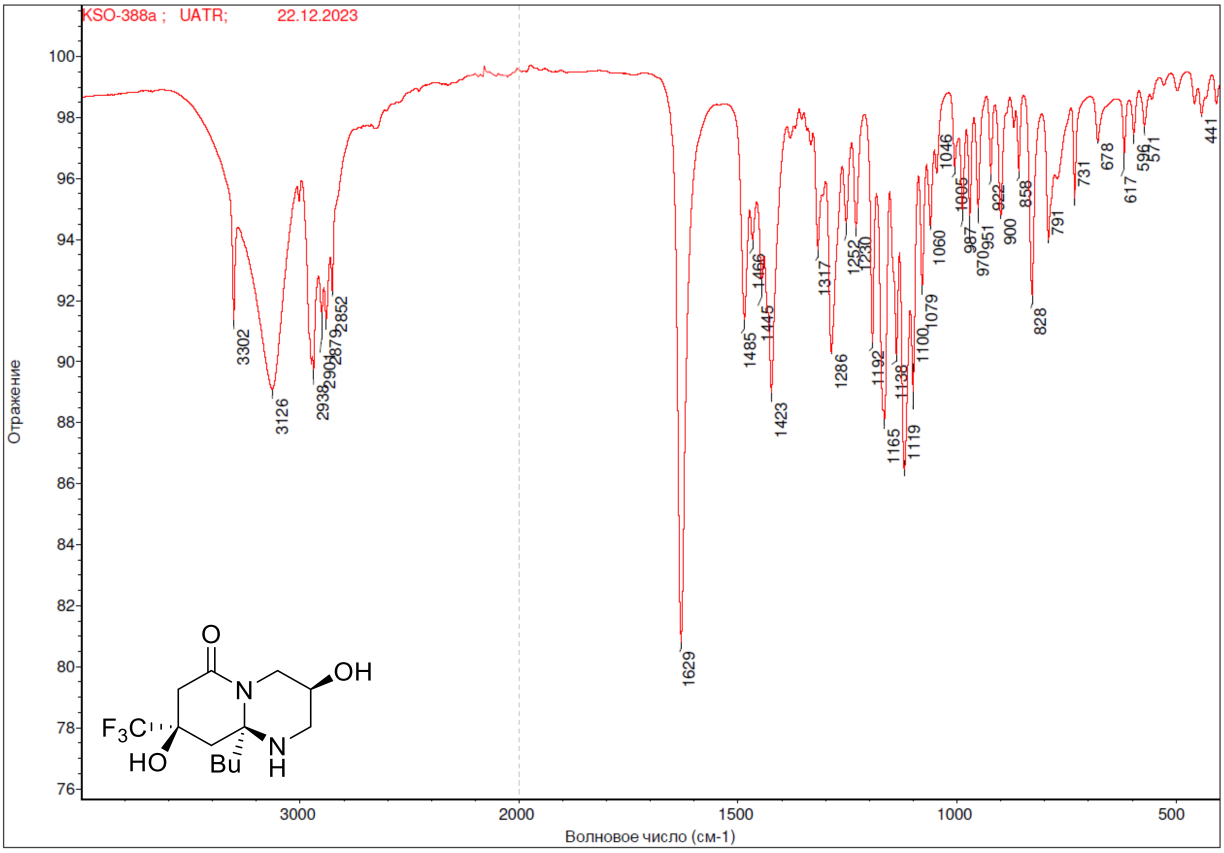

Figure S60: IR spectrum of **4c**^{ct}.

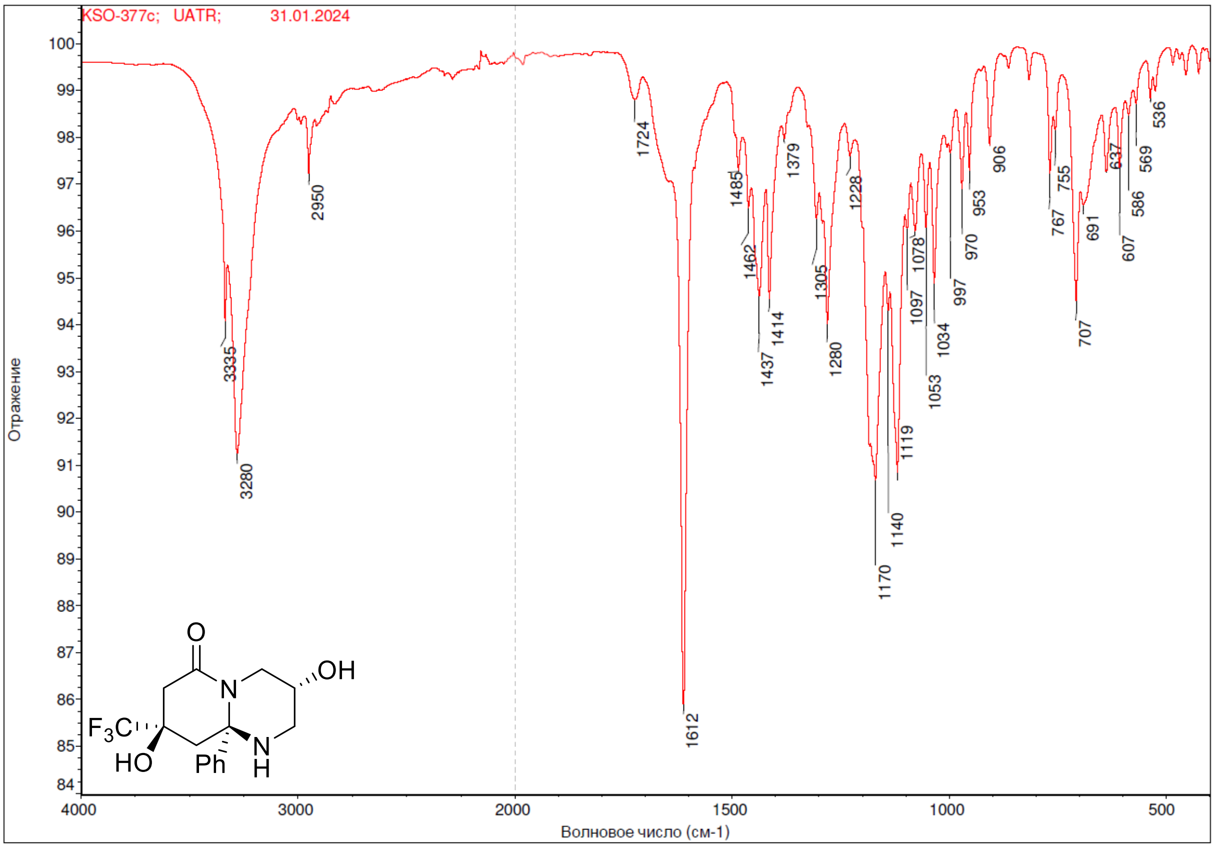

Figure S61: IR spectrum of **4d**^{cc}.

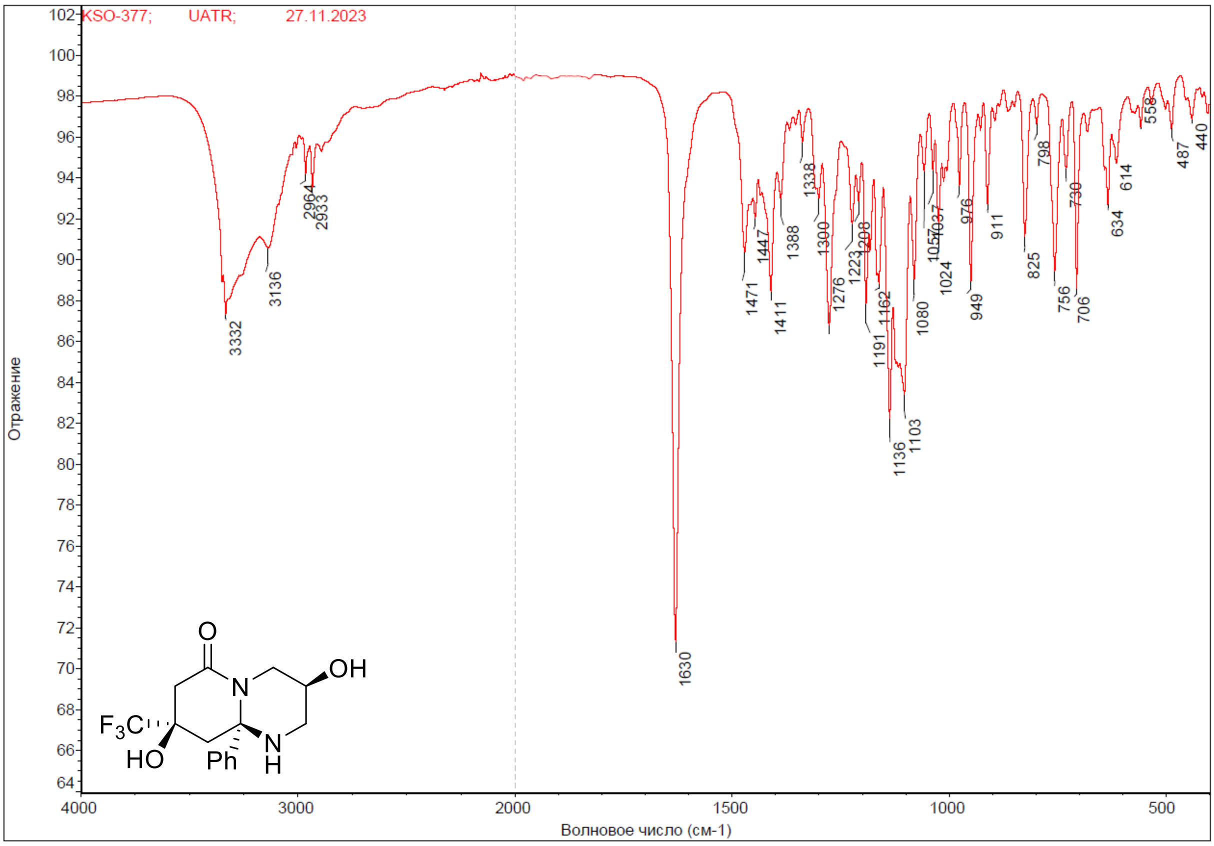

Figure S62: IR spectrum of **4d**^{ct}.

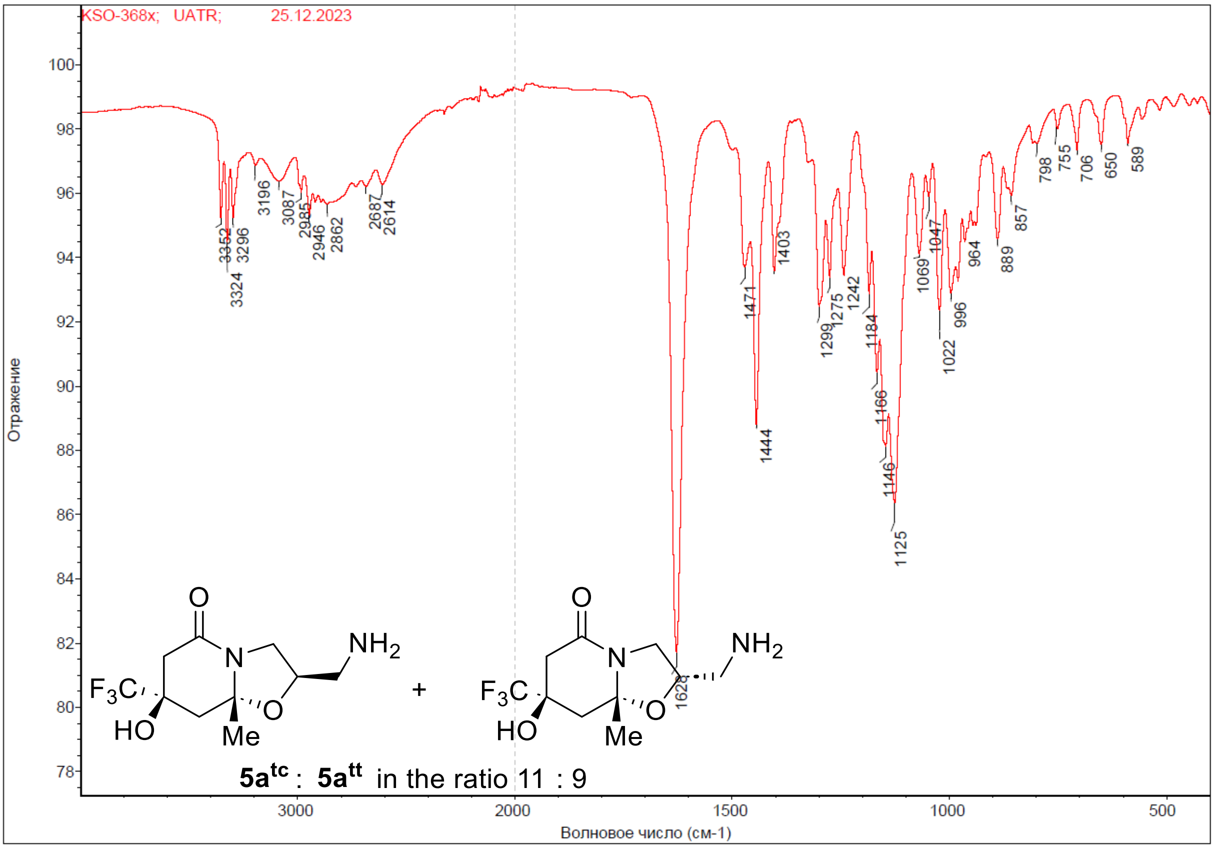

Figure S63: IR spectrum of a mixture of diastereomers **5a**^{tc} and **5a**^{tt}.

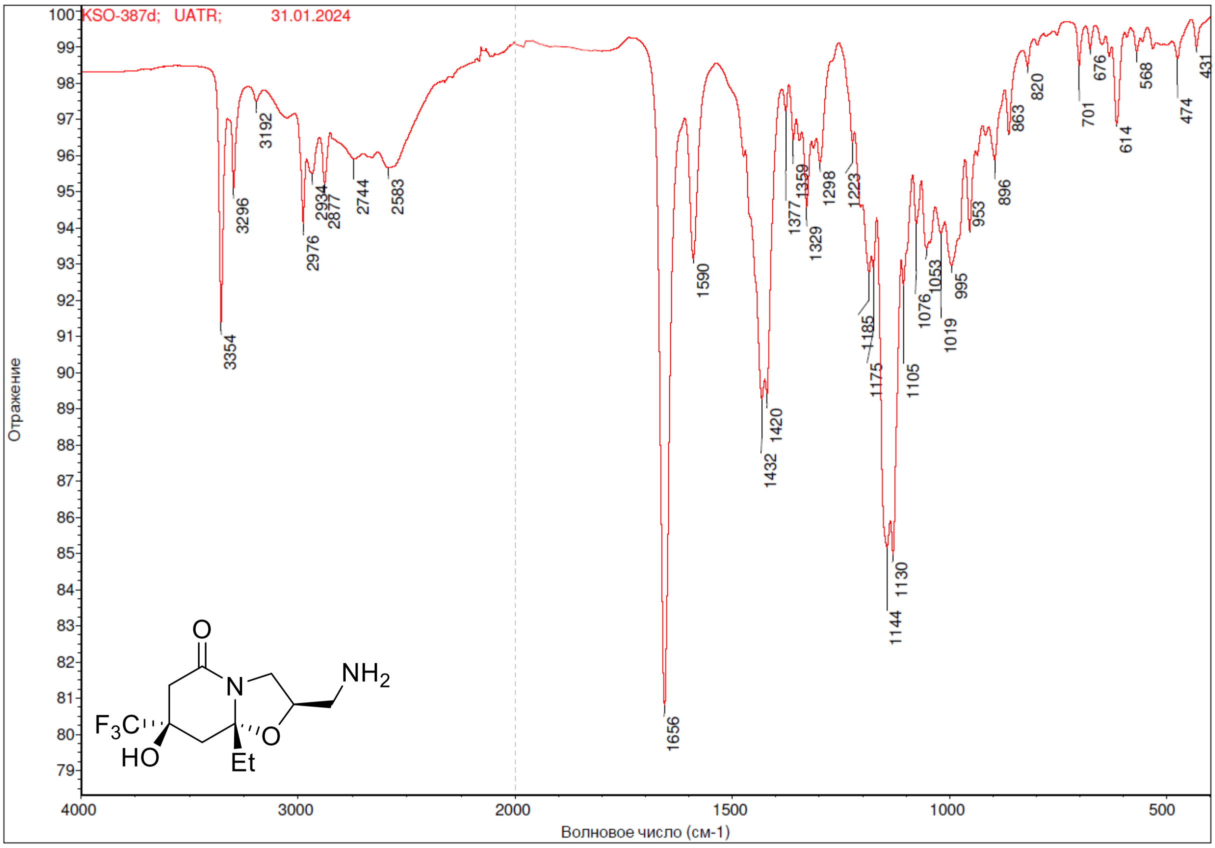

Figure S64: IR spectrum of **5b**[tc].

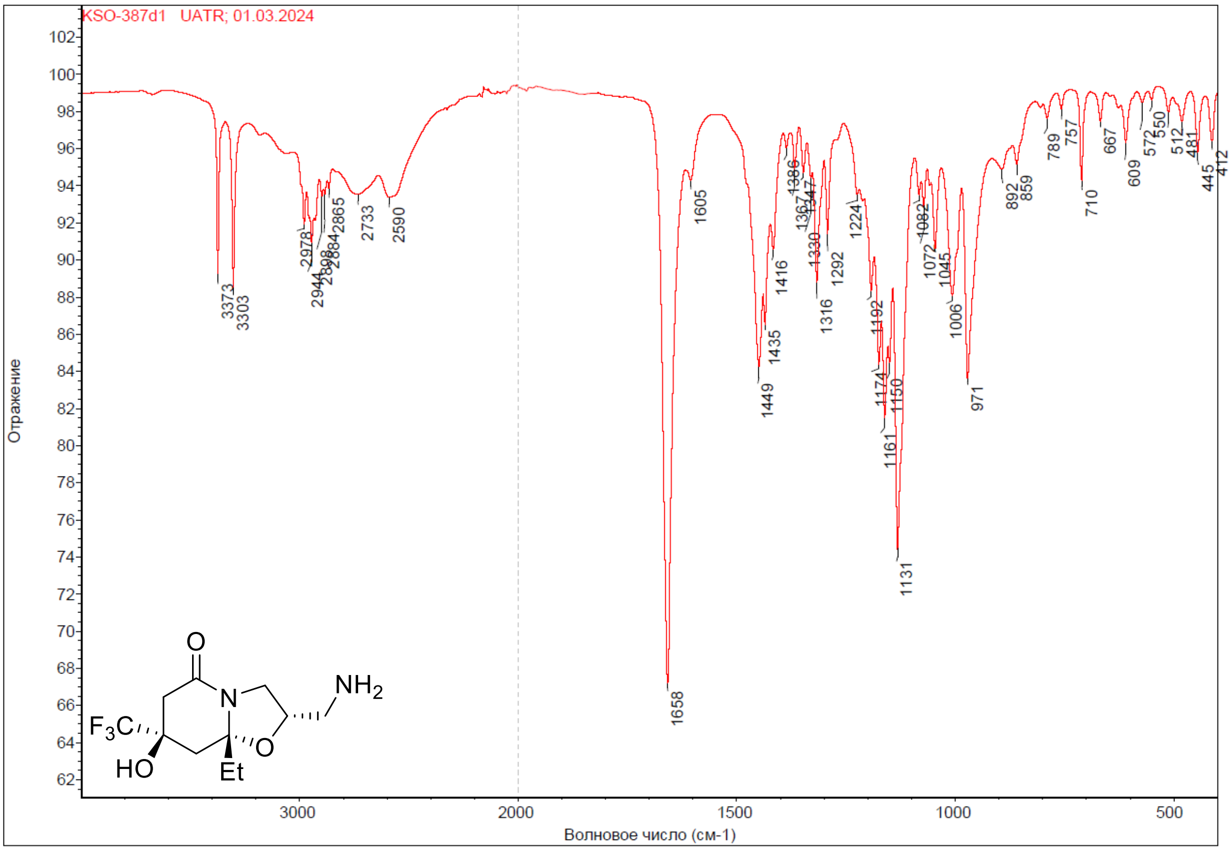

Figure S65: IR spectrum of **5b**[tt].

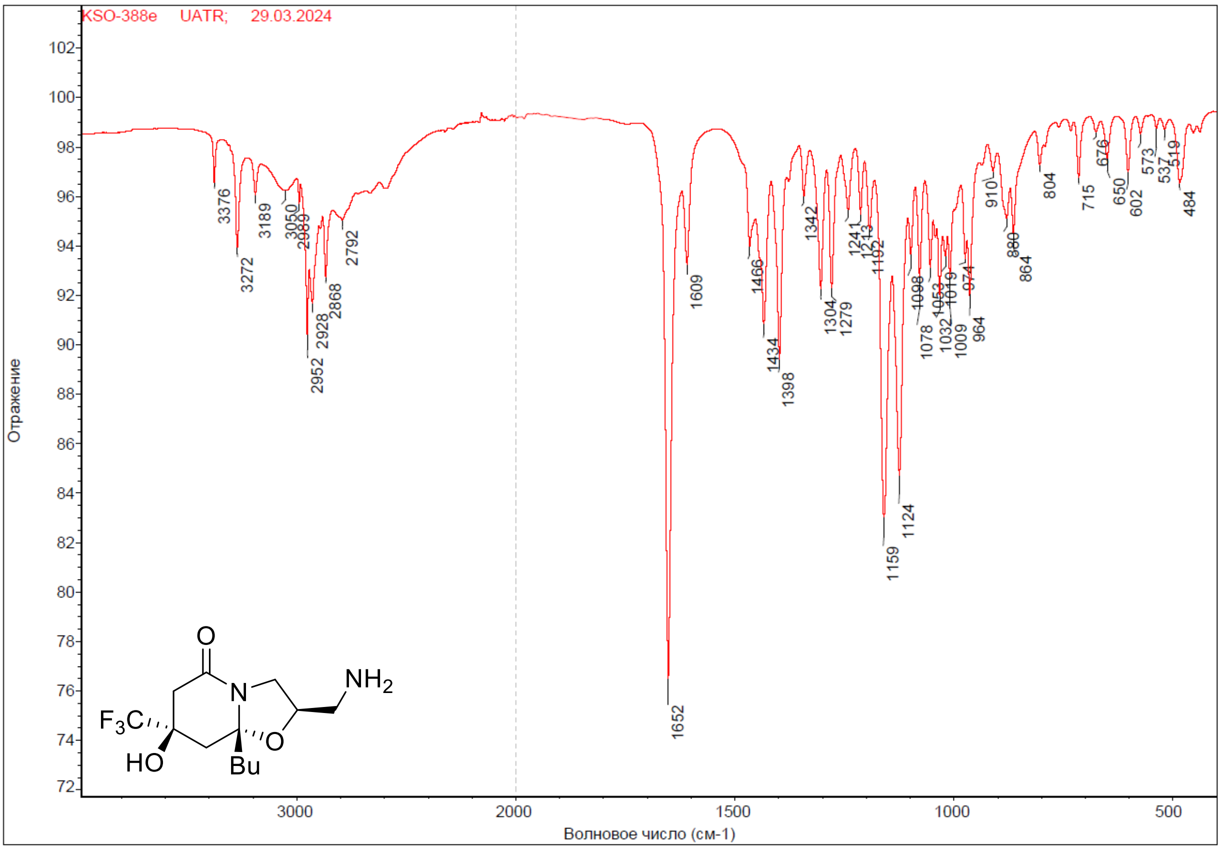

Figure S66: IR spectrum of **5c**^{tc}.

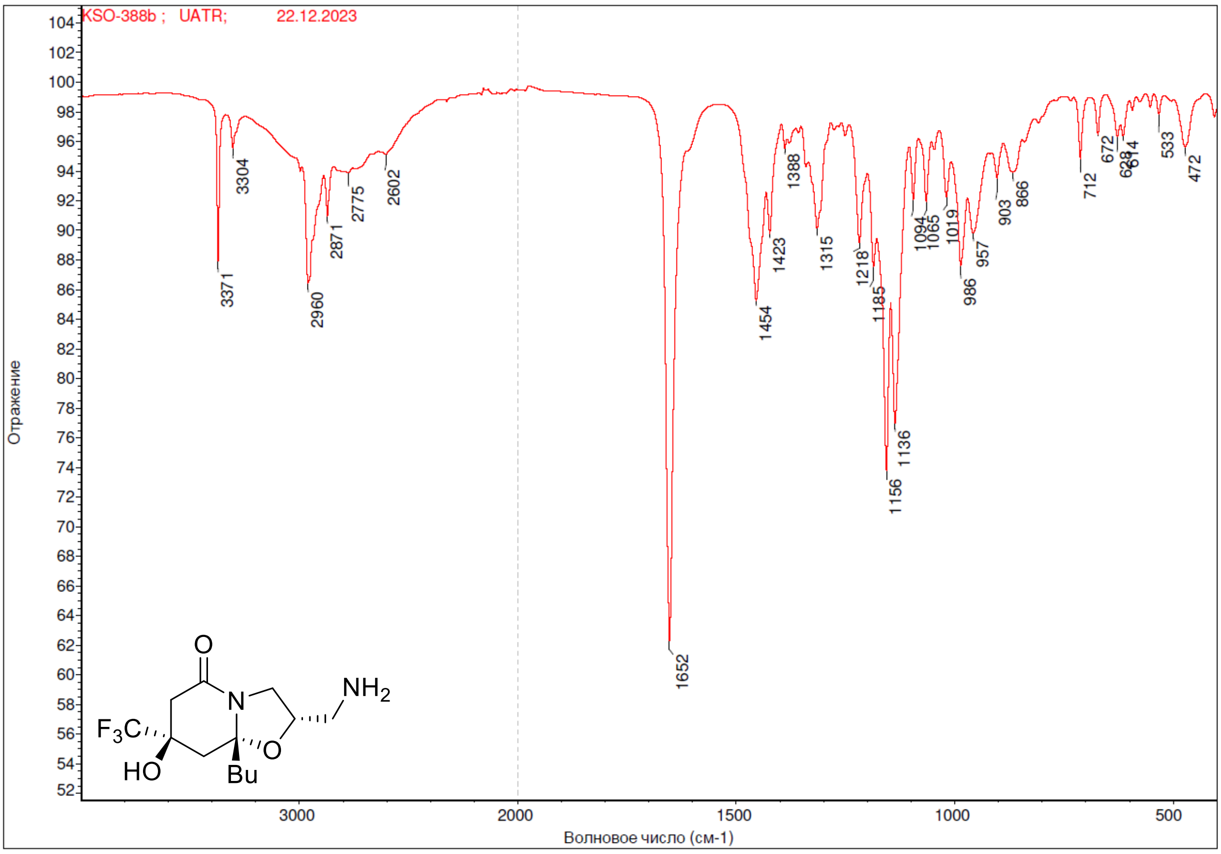

Figure S67: IR spectrum of **5c**^{tt}.

Copies of HRMS spectra

Cmpd 1, 0.5 min

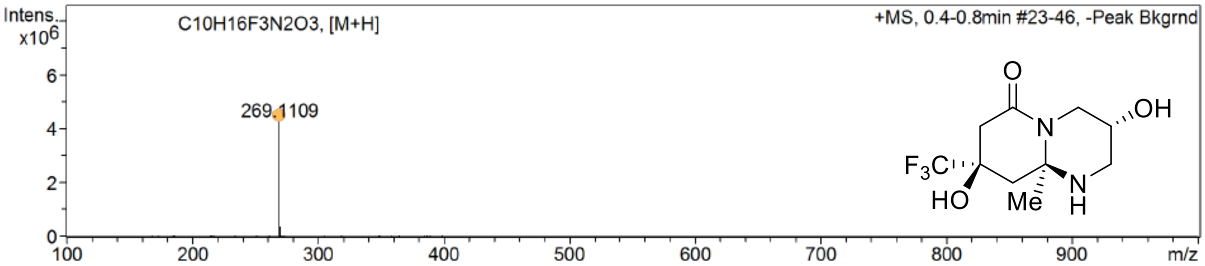

Meas. m/z	#	Ion Formula	m/z	err [ppm]	mSigma	# mSigma	Score	rdb	e⁻ Conf	N-Rule
269.1109	1	C10H16F3N2O3	269.1108	-0.6	14.0	1	100.00	2.5	even	ok

Figure S68: HRMS (ESI) spectrum of **4a**^{cc}.

Cmpd 1, 1.1 min

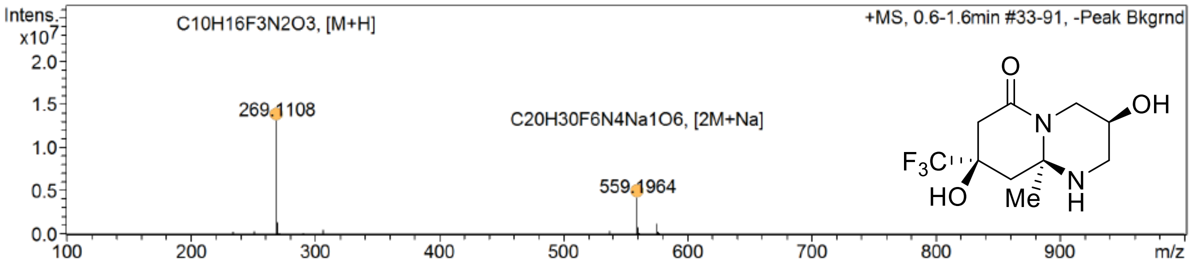

Meas. m/z	#	Ion Formula	m/z	err [ppm]	mSigma	# mSigma	Score	rdb	e⁻ Conf	N-Rule
269.1108	1	C10H16F3N2O3	269.1108	-0.2	4.6	1	100.00	2.5	even	ok
559.1964	1	C20H30F6N4NaO6	559.1962	-0.3	28.0	1	100.00	4.5	even	ok

Figure S69: HRMS (ESI) spectrum of **4a**^{ct}.

Cmpd 1, 0.8 min

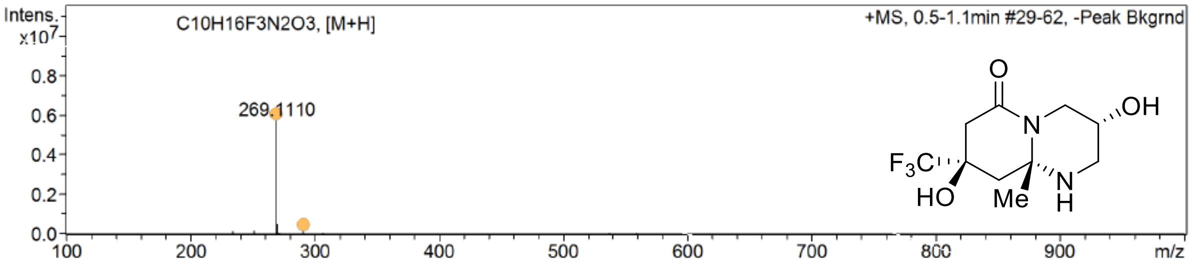

Meas. m/z	#	Ion Formula	m/z	err [ppm]	mSigma	# mSigma	Score	rdb	e⁻ Conf	N-Rule
269.1110	1	C10H16F3N2O3	269.1108	-0.8	14.7	1	100.00	2.5	even	ok
291.0925	1	C10H15F3N2NaO3	291.0927	0.7	3.7	2	92.97	2.5	even	ok

Figure S70: HRMS (ESI) spectrum of **4a**^{tt}.

Cmpd 1, 0.6 min

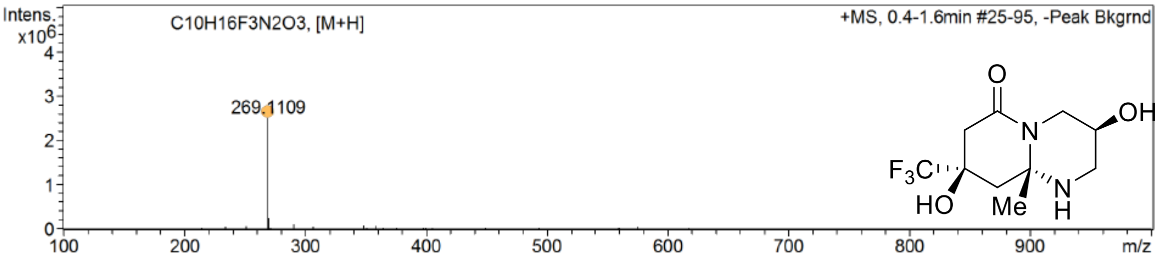

Meas. m/z	#	Ion Formula	m/z	err [ppm]	mSigma	# mSigma	Score	rdb	e⁻ Conf	N-Rule
269.1109	1	C10H16F3N2O3	269.1108	-0.6	8.9	1	100.00	2.5	even	ok

Figure S71: HRMS (ESI) spectrum of **4a**^{tc}.

Cmpd 1, 0.7 min

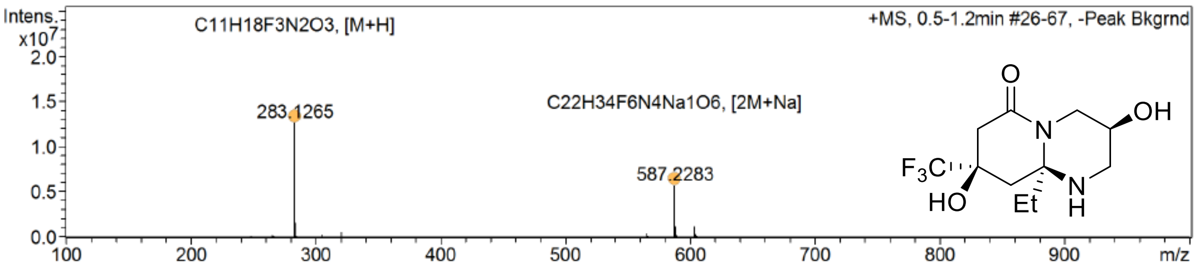

Meas. m/z	#	Ion Formula	m/z	err [ppm]	mSigma	# mSigma	Score	rdb	e⁻ Conf	N-Rule
283.1265	1	C11H18F3N2O3	283.1264	-0.5	1.8	1	100.00	2.5	even	ok
587.2283	1	C22H34F6N4NaO6	587.2275	-1.3	26.1	1	100.00	4.5	even	ok

Figure S72: HRMS (ESI) spectrum of **4b^{ct}**.

Cmpd 1, 0.6 min

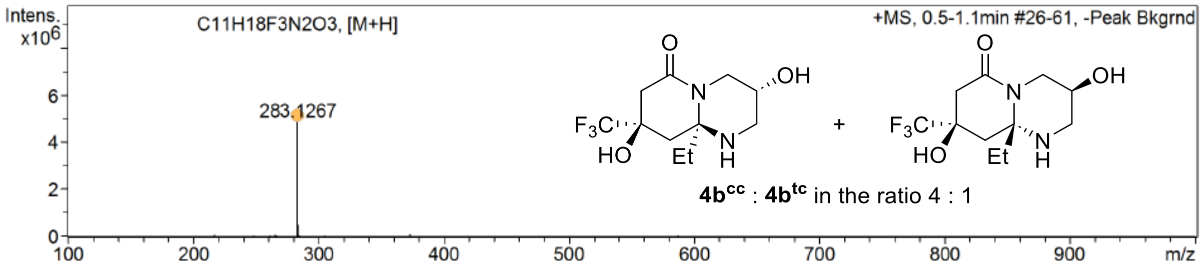

Meas. m/z	#	Ion Formula	m/z	err [ppm]	mSigma	# mSigma	Score	rdb	e⁻ Conf	N-Rule
283.1267	1	C11H18F3N2O3	283.1264	-0.9	12.7	2	92.07	2.5	even	ok

Figure S73: HRMS (ESI) spectrum of a mixture of diastereomers **4b^{cc}** and **4b^{tc}**.

Cmpd 1, 0.6 min

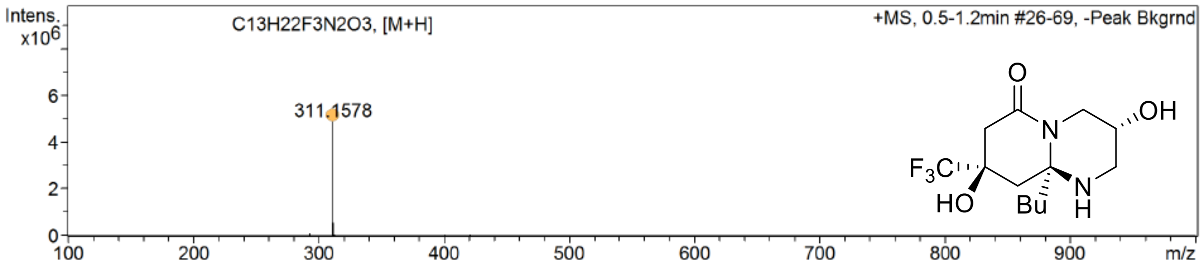

Meas. m/z	#	Ion Formula	m/z	err [ppm]	mSigma	# mSigma	Score	rdb	e⁻ Conf	N-Rule
311.1578	1	C13H22F3N2O3	311.1577	-0.3	17.9	3	88.41	2.5	even	ok

Figure S74: HRMS (ESI) spectrum of **4c^{cc}**.

Cmpd 1, 0.7 min

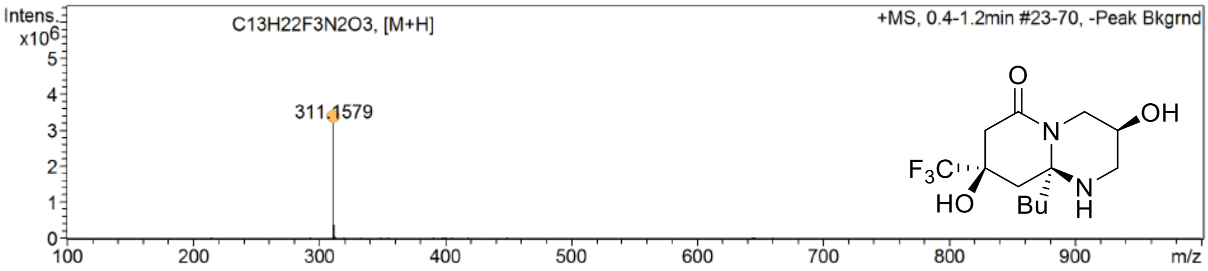

Meas. m/z	#	Ion Formula	m/z	err [ppm]	mSigma	# mSigma	Score	rdb	e⁻ Conf	N-Rule
311.1579	1	C13H22F3N2O3	311.1577	-0.7	14.2	2	100.00	2.5	even	ok

Figure S75: HRMS (ESI) spectrum of **4c^{ct}**.

Cmpd 1, 0.6 min

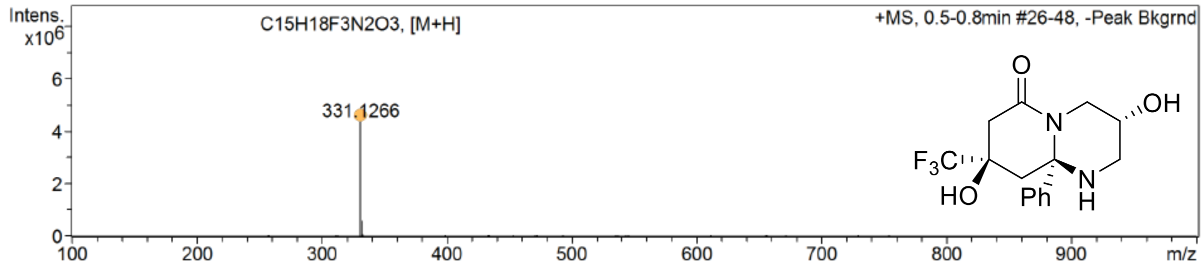

Meas. m/z	#	Ion Formula	m/z	err [ppm]	mSigma	# mSigma	Score	rdb	e⁻ Conf	N-Rule
331.1266	1	C15H18F3N2O3	331.1264	-0.7	20.4	5	72.27	6.5	even	ok

Figure S76: HRMS (ESI) spectrum of **4d**^{cc}.

Cmpd 1, 0.5 min

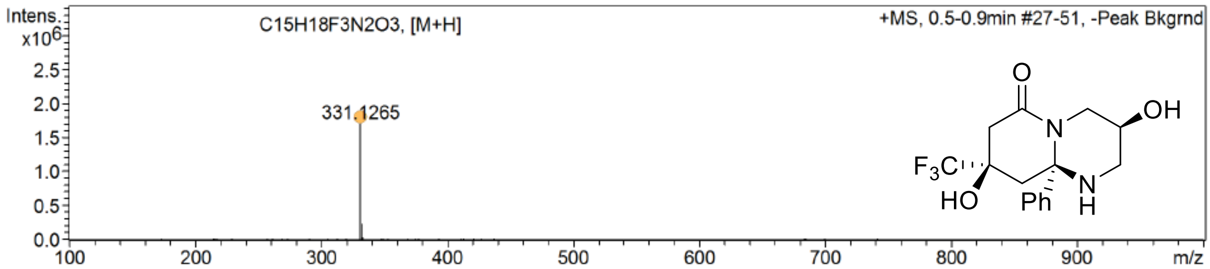

Meas. m/z	#	Ion Formula	m/z	err [ppm]	mSigma	# mSigma	Score	rdb	e⁻ Conf	N-Rule
331.1265	1	C15H18F3N2O3	331.1264	-0.4	15.5	2	76.34	6.5	even	ok

Figure S77: HRMS (ESI) spectrum of **4d**^{ct}.

Cmpd 2, 2.1 min

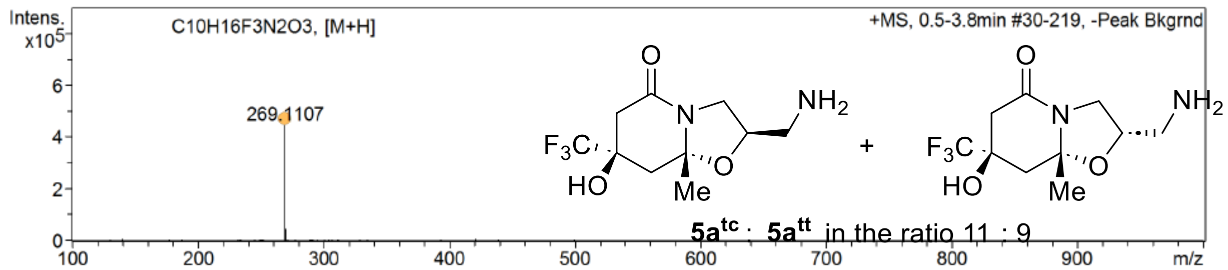

Meas. m/z	#	Ion Formula	m/z	err [ppm]	mSigma	# mSigma	Score	rdb	e⁻ Conf	N-Rule
269.1107	1	C10H16F3N2O3	269.1108	0.1	5.7	2	89.67	2.5	even	ok

Figure S78: HRMS (ESI) spectrum of mixture of diastereomers **5a**^{tc} and **5a**^{tt}.

Cmpd 1, 1.4 min

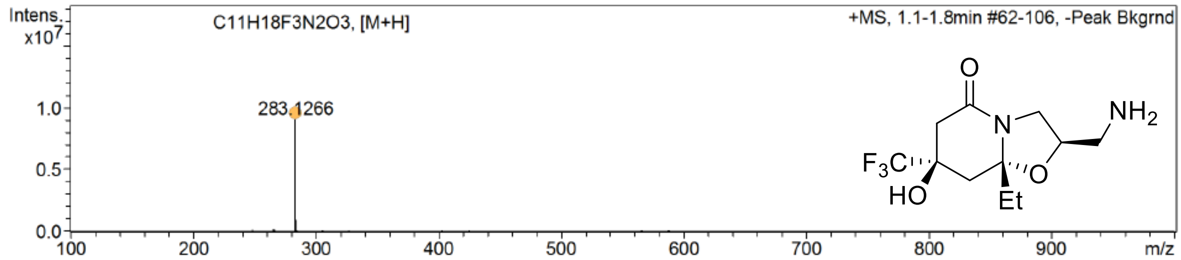

Meas. m/z	#	Ion Formula	m/z	err [ppm]	mSigma	# mSigma	Score	rdb	e⁻ Conf	N-Rule
283.1266	1	C11H18F3N2O3	283.1264	-0.8	10.9	2	90.03	2.5	even	ok

Figure S79: HRMS (ESI) spectrum of **5b**^{tc}.

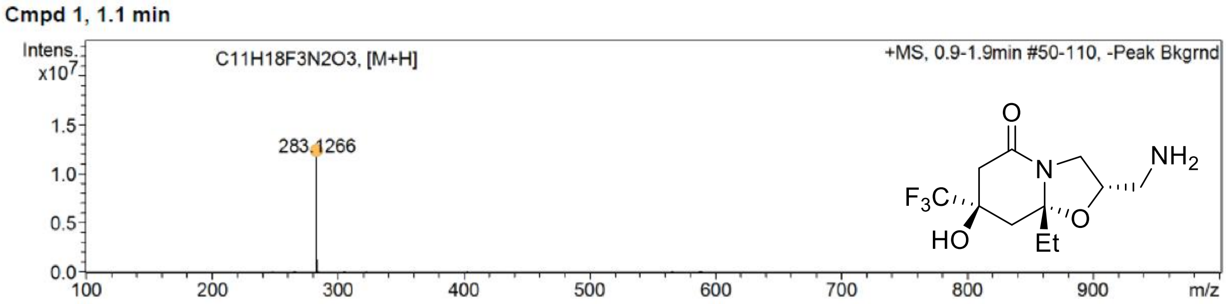

Meas. m/z	#	Ion Formula	m/z	err [ppm]	mSigma	# mSigma	Score	rdb	e⁻ Conf	N-Rule
283.1266	1	C11H18F3N2O3	283.1264	-0.8	9.3	1	92.22	2.5	even	ok

Figure S80: HRMS (ESI) spectrum of **5b**^{tt}.

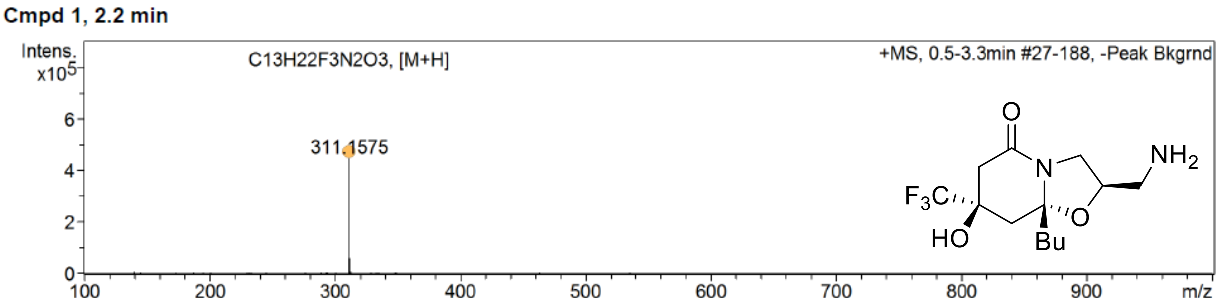

Meas. m/z	#	Ion Formula	m/z	err [ppm]	mSigma	# mSigma	Score	rdb	e⁻ Conf	N-Rule
311.1575	1	C13H22F3N2O3	311.1577	0.6	6.6	2	95.14	2.5	even	ok

Figure S81: HRMS (ESI) spectrum of **5c**^{tc}.

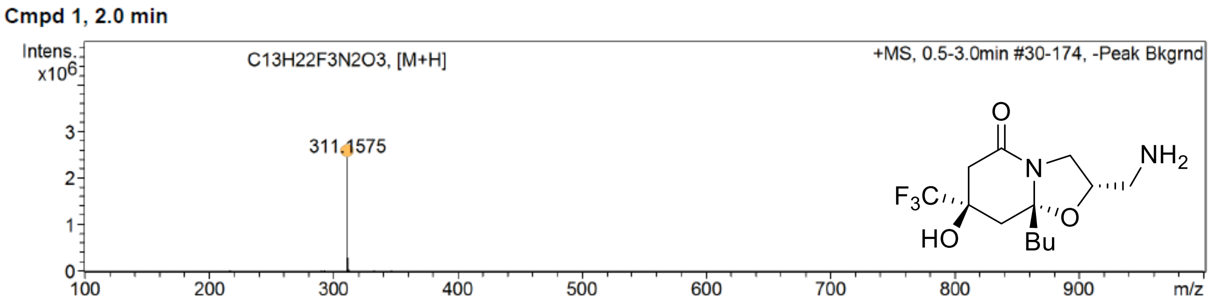

Meas. m/z	#	Ion Formula	m/z	err [ppm]	mSigma	# mSigma	Score	rdb	e⁻ Conf	N-Rule
311.1575	1	C13H22F3N2O3	311.1577	0.8	16.8	2	90.50	2.5	even	ok

Figure S82: HRMS (ESI) spectrum of **5c**^{tt}.
